# Temporal trends in short- and long-term outcomes after carotid interventions for symptomatic or asymptomatic stenosis: a systematic review and meta-analysis

**DOI:** 10.1093/esj/aakaf002

**Published:** 2026-01-01

**Authors:** Carolijn J M de Bresser, Robbert B M Wiggers, Roos A M van Heeswijk, Barend M Mol, Fleur J W Knol, Gert J de Borst, Michiel H F Poorthuis

**Affiliations:** Department of Vascular Surgery, University Medical Center Utrecht, Heidelberglaan 100, 3584 CX Utrecht, The Netherlands; Department of Vascular Surgery, University Medical Center Utrecht, Heidelberglaan 100, 3584 CX Utrecht, The Netherlands; Department of Vascular Surgery, University Medical Center Utrecht, Heidelberglaan 100, 3584 CX Utrecht, The Netherlands; Department of Vascular Surgery, University Medical Center Utrecht, Heidelberglaan 100, 3584 CX Utrecht, The Netherlands; Department of Vascular Surgery, University Medical Center Utrecht, Heidelberglaan 100, 3584 CX Utrecht, The Netherlands; Department of Vascular Surgery, University Medical Center Utrecht, Heidelberglaan 100, 3584 CX Utrecht, The Netherlands; Reinier de Graaf Gasthuis, Reinier de Graafweg 5, 2625 AD Delft, The Netherlands; Department of Neurology, University Medical Center Utrecht, Heidelberglaan 100, 3584 CX Utrecht, The Netherlands

**Keywords:** carotid artery stenosis, carotid endarterectomy, carotid artery stenting, systematic review, meta-analysis

## Abstract

**Introduction:**

In meta-analyses of large cohorts, a decline in procedural risks after carotid endarterectomy (CEA) was found. It remains unclear whether these trends extent to smaller cohorts, carotid artery stenting (CAS), and how long-term outcomes have evolved.

**Patients and methods:**

PubMed and EMBASE were searched until 18 November 2024, for studies reporting on 100 or more adults undergoing CEA or CAS for symptomatic or asymptomatic carotid stenosis. Primary outcomes were 30-day and long-term risk of stroke or death. We performed separate analyses in smaller cohorts of < 500 patients.

**Results:**

291 studies reported 475,266 patients undergoing CEA (214,526 symptomatic, 260,740 asymptomatic) and 209,117 undergoing CAS (77,133 symptomatic, 131,984 asymptomatic). Short-term stroke or death after CEA declined 36% (RR = 0.64, 95% CI, 0.63–0.64) per 5-year later treatment midyear in symptomatic and 41% (RR = 0.59, 95% CI, 0.59–0.59) in asymptomatic patients, with consistent trends in smaller cohorts.For CAS, short-term risks declined 44% (RR = 0.56, 95% CI, 0.53–0.58) in symptomatic, and 27% (RR = 0.73, 95% CI, 0.71–0.74) in asymptomatic patients, with consistent trends in smaller cohorts. Long-term death risk after CEA increased 26% (RR = 1.26, 95% CI, 1.20–1.32) and 11% in smaller cohorts. Long-term stroke risk after CAS increased 30% (RR = 1.30, 95% CI, 1.17–1.43) and 44% in smaller cohorts.

**Conclusions:**

Short-term risks after CEA and CAS have decreased over time, also in smaller cohorts. Long-term death after CEA and stroke after CAS have increased. The increased long-term risk of death after CEA and stroke after CAS limits the durability of carotid interventions and warrants further scrutiny.

## Graphical Abstract

**Graphical Abstract:**
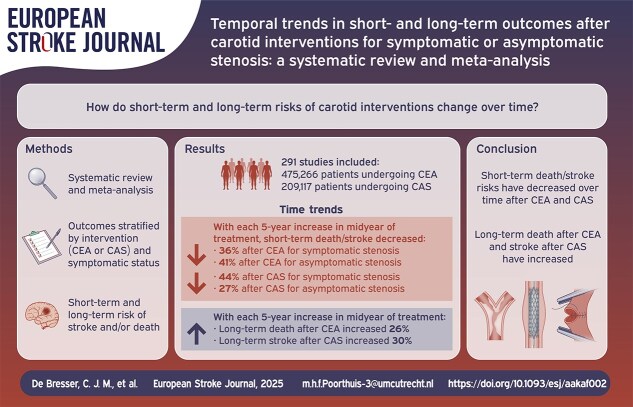

## Introduction

Stroke is a leading global health concern, ranking as the second leading cause of death and the third leading cause of combined death and disability worldwide.^1^^,^^2^ Carotid artery stenosis accounts for 15%-20% of ischemic strokes and is associated with a high risk of early stroke recurrence.^3–6^ As of 2020, the global prevalence of carotid stenosis is 1.5%, 58 million cases, reflecting a 59% increase since 2000.^7–9^

Carotid endarterectomy (CEA) and carotid artery stenting (CAS) aim to reduce long-term stroke, but carry early procedural risks. Carotid endarterectomy remains widely performed, with over 80,000 procedures annually in the United States.^10^ While CEA has been the gold standard treatment, CAS is an alternative for patients unsuitable for CEA and in younger patients.^11^

Procedural risks after CEA have declined over time, but have only been assessed in papers published before May 2016 and in large observational cohorts of over 1000 patients.^12^ Secular temporal trends are unclear, as is whether improvements are generalisable to smaller cohorts.^12^ In addition, no studies to date have assessed temporal trends in patients undergoing CAS. Finally, long-term outcomes have not been assessed previously. Data on long-term outcomes after carotid interventions, especially outside RCTs, are scarce.^13–17^ Long-term risk of stroke determines the efficacy of carotid interventions, and life expectancy, especially crucial in asymptomatic patients who lack the high early recurrence risk of symptomatic patients, must outweigh short-term procedural risks.^18^^,^^19^

We aimed to systematically review the short- and long-term risks of stroke and/or death after CEA and CAS in patients with symptomatic and asymptomatic carotid stenosis and determine temporal trends.

## Patients and methods

This systematic review and meta-analysis followed a predefined protocol registered prospectively in the International Prospective Registry for Systematic Reviews (PROSPERO: CRD42024574340), in accordance with the Preferred Reporting Items for Systematic Reviews and Meta-Analysis (Table S1) and the Meta-analysis Of Observational Studies in Epidemiology guidelines.^18^^,^^19^ Data not published are available upon reasonable request.

### Data sources and searches

PubMed and EMBASE were searched from inception until 18 November 2024, for observational studies and RCTs meeting predefined eligibility criteria (Table S2). Additional backward citation searching was performed for additional studies.

### Eligibility criteria, screening process and data extraction

Articles were included based on: (1) peer-reviewed and reported original data; (2) studies including ≥100 patients undergoing CEA or CAS for ≥50% atherosclerotic carotid artery stenosis confirmed by duplex ultrasound, computed tomography angiography, magnetic resonance angiography and/or digital subtraction angiography; (3) reported short- or long-term stroke and/or death after intervention and (4) provided separate data for symptomatic and asymptomatic patients.

Articles were excluded if they: (1) lacked incidence rates or raw data to calculate them; (2) included ≥ 10% bilateral, re-do, tandem procedures or combined procedures such as coronary artery bypass grafting or (3) classified patients as symptomatic if symptoms occurred > 6 months before carotid intervention.

Two authors (C.B. and R.W.) independently screened titles and abstracts. Full-text screening was performed in duplicate by 5 reviewers (C.B., R.W., R.H., B.M. and F.K.). Discrepancies were resolved in consensus meetings by C.B., R.W. and M.P.

The extracted data included: (1) study characteristics: study years, number of study centres, geographic area (country and continent), sample size (patients and procedures), follow-up duration (patient-years, median and/or mean); (2) patient characteristics: (A) baseline characteristics: sex, age and cardiovascular risk factors (current smoking, diabetes, congestive heart failure); (B) disease characteristics: symptomatic status, type of qualifying event (ischaemic stroke, transient ischaemic attack or ocular symptoms), degree of stenosis, contralateral occlusion and (C) outcomes: incidence and event counts, with timeframes specified.

### Outcome measures

The primary endpoint was the composite of stroke or death (stratified by timing of occurrence in-hospital or within 30 days after intervention) and the long-term (beyond 30 days). Secondary endpoints were the components of death and stroke.

### Overlapping data

We documented for each study the trial or registry name, participating institution(s), country of origin, year of publication and recruitment period to detect potential overlapping patient samples. Studies from the same registry were cross-checked for overlapping recruitment periods and outcome measures, with original reports prioritised over subgroup analyses. All studies were organised by country, and for multicenter studies, participating institutions were cross-referenced against other included studies to detect potential overlap. Studies were considered duplicates only when overlapping outcomes were reported. Studies were included when different outcomes were reported.

### Risk of bias

Three reviewers (C.B., R.W. and R.H.) independently assessed the risk of bias using a modified Methodological Index for Non-Randomised Studies (MINORS) quality assessment tool. Each cohort was rated as low, intermediate or high risk of bias across 9 domains: (1) patient selection (representativeness of the cohort undergoing carotid interventions versus restriction to high-risk patients), (2) method of patient inclusion (consecutiveness enrolment or random sampling), (3) baseline reporting (availability of baseline characteristics on cohort-level (separately provided for patients undergoing CEA and CAS, and for patients with symptomatic and asymptomatic carotid stenosis)), (4) prospective data collection, (5) degree of stenosis classified according to reporting standards (50%-69% for moderate stenosis, 70%-99% for severe stenosis), (6) stroke definition provided, (7) outcome ascertainment by an independent neurologist or endpoint adjudication committee, (8a) specification of the short-term timeframe (30 days versus in-hospital), (8b) long-term follow-up (reported as patient-years or incidence rates versus mean or median duration of follow-up) and (9) reporting and handling loss to follow-up (Table S3).^20^

### Statistical analysis

Continuous variables were summarised as mean (standard deviation), skewed data as median (interquartile range) and absolute numbers with proportions for categorical variables. Cohort characteristics were described using descriptive statistics.

Analyses were stratified by type of intervention (CEA and CAS) and symptomatic status (symptomatic and asymptomatic carotid stenosis). Analyses of short-term outcomes were also stratified by timing of occurrence (in-hospital and 30 days). The restricted maximum likelihood random-effects method with a DerSimonian–Laird estimator and a Freeman–Tukey double arcsine transformation was used to obtain pooled estimates of short-term risks of the composite of death or stroke, and its components, with confidence intervals based on Wilson Score.

We quantified the occurrence of composite of long-term death or stroke (beyond 30 days), and its components, for which we used the annual incidence rates if reported. We used reported total person-years (PY) of follow-up if annual incidence rates were not provided. We multiplied the median or mean follow-up period by the total number of treated patients if total PY were not provided. We calculated the incidence rates of the composite of long-term death or stroke, and its components, with 95% CIs per 100 PY of follow-up using a Poisson regression model.

To assess temporal trends, we used the midyear of treatment (the middle of the time frame of year of patient treatment) as the dependent variable in a linear regression (short-term outcomes) and a Poisson meta-regression analysis (long-term outcomes). Temporal trend analyses were performed if ≥20 cohorts were available. We repeated analyses for smaller cohorts (with 100 or more and less than 500 patients). All statistical analyses were performed using STATA (version 15.1) and figures were created using R.

## Results

**Figure 1:**
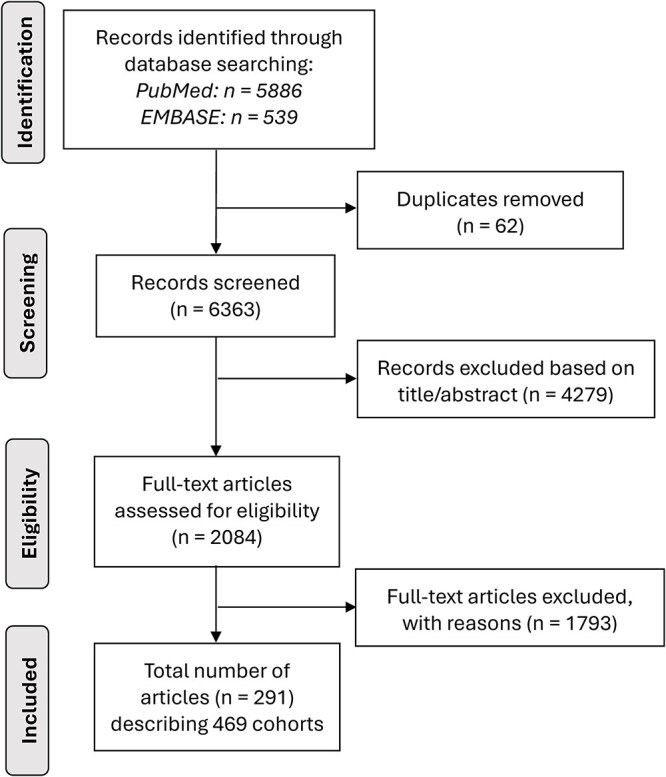
Flowchart of literature review to identify the included studies.

After screening 6425 unique reports and assessing the full text of 2084 for eligibility, we included 291 studies reporting 466 cohorts, of which 239 cohorts described 475,266 patients who underwent CEA and 227 cohorts described 209,117 patients who underwent CAS (Figure 1; Table S4). Reasons for exclusion of 1793 articles during full-text evaluation are provided in Table S5.

**Figure 2:**
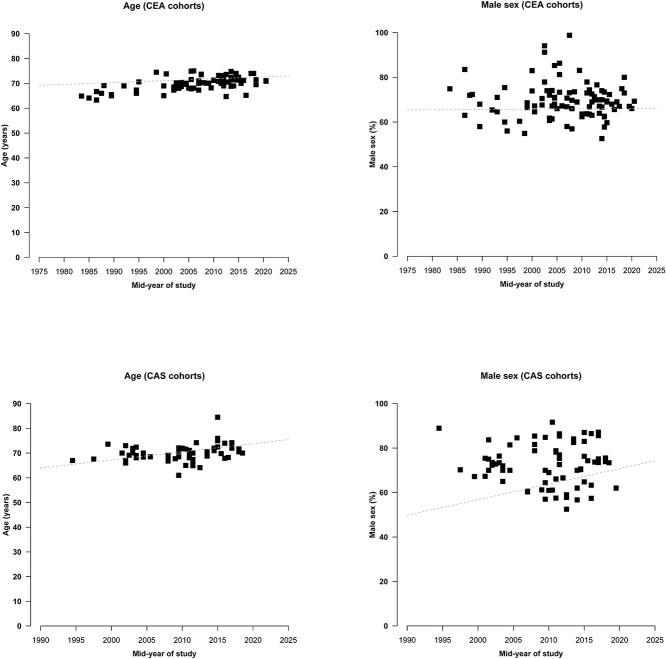
Temporal trends in age and sex, by type of carotid intervention. Graphs showing temporal trends in age and percentage of male sex by midyear of treatment in cohorts of patients undergoing CEA (82 and 97 cohorts, respectively) and CAS (60 and 73 cohorts, respectively). Age increased 0.4 (CEA) and 1.6 (CAS) with each 5-year increase in midyear. The percentage of male patients increased 0.1% (CEA) and 3.5% (CAS) with each 5-year increase in midyear. Abbreviations: CEA = carotid endarterectomy; CAS = carotid artery stenting.

Mean or median age increased by 0.4 years per 5-year increment in midyear in 82 cohorts describing patients undergoing CEA. The percentage of male patients increased by 0.1% per 5-year increment in 97 cohorts (Figure 2). Mean or median age increased by 1.6 years per 5-year increment in midyear in 60 cohorts describing patients undergoing CAS. The percentage of male patients increased 3.5% per 5-year increment in 73 cohorts (Figure 2).

### Short-term outcomes

**Figure 3:**
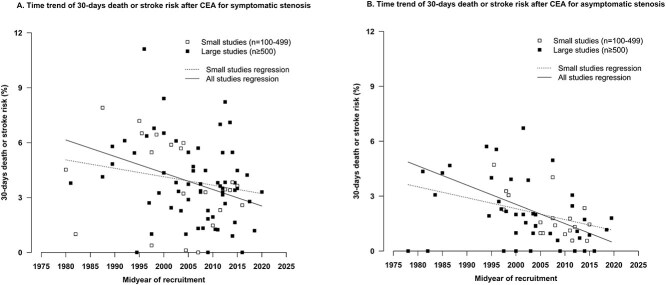
Temporal trends of 30-day death or stroke after CEA for (A) symptomatic stenosis and (B) asymptomatic stenosis. The RR of 30-day death or stroke after CEA in symptomatic patients was 0.64 (95% CI, 0.63-0.64) with each 5-year more recent midyear of treatment in all 85 cohorts and 0.79 (95% CI, 0.78-0.81) in 63 cohorts with fewer than 500 patients. The RR of 30-day death or stroke after CEA in asymptomatic patients was 0.59 (95% CI, 0.59-0.59) with each 5-year more recent midyear of treatment in all 61 cohorts and 0.74 (95% CI, 0.73-0.76) in 42 cohorts with fewer than 500 patients. Abbreviations: CEA = carotid endarterectomy; CAS = carotid artery stenting; CI = confidence interval.

The pooled estimate of the composite short-term death or stroke after CEA in patients with symptomatic stenosis was 3.4% (95% CI, 3.0-3.8; 88 cohorts). A decrease of 36% (RR = 0.64, 95% CI, 0.63-0.64; 85 cohorts) with each 5-year more recent midyear of treatment was found, and a decrease of 21% (RR = 0.79, 95% CI, 0.78-0.81; 63 cohorts) in cohorts with fewer than 500 patients (Figure 3A; Table 1; Table S6).

**Table 1:** Incidence rate of short-term outcomes after CEA and CAS.

	**Total number of cohorts**	**Total number of outcome events**	**Total number of patients**	**Pooled estimate (95% CI)** ^**a**^	***I*** ^**2**^ **(%)**
***CEA—symptomatic patients***					
**Death or stroke**					
*30-day*	88	3342	95,065	3.4 (3.0-3.8)	88
*In-hospital*	10	3648	118,406	2.9 (2.4-3.4)	86
**Death**					
*30-day*	99	826	94,849	0.6 (0.5-0.7)	59
*In-hospital*	12	688	93,994	0.5 (0.3-0.64	65
**Stroke**					
*30-day*	104	2585	95,793	2.7 (2.4-3.0)	84
*In-hospital*	10	1754	76,833	2.0 (1.4-2.7)	76
***CEA—asymptomatic patients***					
**Death or stroke**					
*30-days*	66	1519	96,582	1.5 (1.2-1.9)	84
*In-hospital*	8	2873	195,404	1.3 (1.1-1.5)	71
**Death**					
*30-day*	72	276	64,899	0.1 (0.1-0.2)	62
*In-hospital*	9	593	178,220	0.1 (0.1-0.4)	93
**Stroke**					
*30-day*	74	1134	101,415	1.1 (0.8-1.3)	82
*In-hospital*	*7*	*1195*	105,855	1.2 (0.9-1.6)	59
***CAS—symptomatic patients***					
**Death or stroke**					
*30-day*	74	1373	25,779	4.7 (4.0-5.4)	81
*In-hospital*	8	946	18,808	2.4 (1.0-4.2)	85
**Death**					
*30-day*	85	750	45,256	0.8 (0.6-1.1)	76
*In-hospital*	12	725	15,783	0.8 (0.0-3.1)	98
**Stroke**					
*30-day*	89	1369	35,341	3.9 (3.3-4.6)	83
*In-hospital*	8	567	17,770	2.1 (1.3-3.1)	23
***CAS—asymptomatic patients***					
**Death or stroke**					
*30-day*	66	929	31,738	2.1 (1.7-2.5)	73
*In-hospital*	8	391	23,372	0.7 (0.1-1.7)	81
**Death**					
*30-day*	74	417	56,989	0.2 (0.1-0.3)	60
*In-hospital*	14	387	48,871	0.2 (0.0-0.5)	81
**Stroke**					
*30-day*	77	771	35,720	1.6 (1.3-2.0)	73
*In-hospital*	10	320	23,703	0.6 (0.1-1.4)	75

Abbreviations: CI = confidence interval; CAS = carotid artery stenting; CEA = carotid endarterectomy.

^a^Restricted maximum likelihood random-effects method with Freeman–Tukey double arcsine transformation was used to obtain pooled estimates of procedural complications and confidence intervals are based on Wilson Score.

The pooled estimate of the composite short-term death or stroke after CEA in patients with asymptomatic stenosis was 1.5% (95% CI, 1.2-1.9; 66 cohorts). A decrease of 41% (RR = 0.59, 95% CI, 0.59-0.59; 61 cohorts) with each 5-year more recent midyear of treatment was found, and a decrease of 26% (RR = 0.74, 95% CI, 0.73-0.76; 42 cohorts) in cohorts with fewer than 500 patients (Figure 3B; Table 1; Table S6).

**Figure 4:**
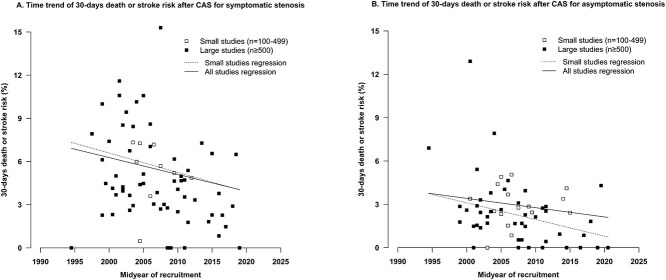
Temporal trends of 30-day death or stroke after CAS for (A) symptomatic stenosis and (B) asymptomatic stenosis. The RR of 30-day death or stroke after CAS in symptomatic patients was 0.56 (95% CI, 0.53-0.58) with each 5-year more recent midyear of treatment in all 71 cohorts, and 0.51 (95% CI, 0.47-0.55) in 62 cohorts with fewer than 500 patients. The RR of 30-day death or stroke after CAS in asymptomatic patients was 0.73 (95% CI, 0.71-0.74) with each 5-year more recent midyear of treatment in all 62 cohorts and 0.56 (95% CI, 0.54-0.58) in 46 cohorts with fewer than 500 patients. Abbreviations: CAS = carotid artery stenting; CI = confidence interval.

The pooled estimate of the composite short-term death or stroke after CAS in patients with symptomatic stenosis was 4.7% (95% CI, 4.0%-5.4%; 47 cohorts). A decrease of 44% (RR = 0.56, 95% CI, 0.53-0.58; 71 cohorts) with each 5-year more recent midyear of treatment was found and a decrease of 49% (RR = 0.51, 95% CI, 0.47-0.55; 62 cohorts) in cohorts with fewer than 500 patients (Figure 4A; Table 2; Table S7).

**Table 2:** Incidence rate of long-term outcomes after CEA and CAS.

	**Cohorts**	**Total number of outcome events/PY**	**Total number of patients**	**Annual incidence rate per 100 PY (95% CI)** ^**a**^
***CEA—symptomatic patients***				
Death or stroke	15	423/9059	3472	4.67 (4.24-5.14)
Death	31	2845/73,562	34,177	3.87 (3.73-4.01)
Stroke	30	1184/91,013	34,422	1.30 (1.23-1.38)
***CEA—asymptomatic patients***				
Death or stroke	7	164/3440	1150	4.77 (4.09-5.56)
Death	21	3344/96,137	55,005	3.48 (3.36-3.60)
Stroke	17	349/69,416	30,411	0.50 (0.45-0.56)
***CAS—symptomatic patients***				
Death or stroke	18	478/10,231	4469	4.67 (4.27-5.11)
Death	26	551/16,309	6903	3.38 (3.11-3.67)
Stroke	28	407/21,106	7363	1.93 (1.75-2.13)
***CAS—asymptomatic patients***				
Death or stroke	11	218/8141	3200	2.68 (2.34-3.06)
Death	18	736/22,864	6801	3.22 (2.99-3.46)
Stroke	19	164/15,663	5488	1.05 (0.90-1.22)

Abbreviations: CAS = carotid artery stenting; CEA = carotid endarterectomy; CI = confidence interval; PY = person-years.

^a^Poisson models were used to obtain annual incidence rates of outcomes during follow-up.

The pooled estimate of the composite short-term death or stroke after CAS in patients with asymptomatic stenosis was 2.1% (95% CI, 1.7-2.5; 66 cohorts). A decrease of 27% (RR = 0.73, 95% CI, 0.71-0.74; 62 cohorts) with each 5-year more recent midyear of treatment was found, and a decrease of 44% (RR = 0.56, 95% CI, 0.54-0.58; 46 cohorts) in cohorts with fewer than 500 patients (Figure 4B; Table 2; Table S7).

The risk of short-term death was higher after CAS compared with CEA (except for the risk of in-hospital stroke in patients with asymptomatic stenosis), and higher in patients with symptomatic compared with asymptomatic stenosis (Tables 1 and 2). Temporal trends of short-term death and short-term stroke were generally less pronounced compared with the composite outcome, except for CAS in patients with symptomatic and asymptomatic stenosis (Tables S6 and S7). The risk of death after CAS in cohorts with fewer than 500 patients did not decrease over time (Table S7).

### Long-term outcomes after CEA

The annual incidence rate of long-term stroke or death after CEA was 4.67 (95% CI, 4.24-5.14; 15 cohorts) per 100 PY in symptomatic and 4.77 (95% CI, 4.09-5.56; 7 cohorts) in asymptomatic patients (Table 2). The annual incidence rates of long-term death and long-term stroke after CEA were 3.87 (95% CI, 3.73-4.01; 31 cohorts) and 1.30 (95% CI, 1.23-1.38; 30 cohorts) in symptomatic, and 3.48 (95% CI, 3.36-3.60; 21 cohorts) and 0.50 (95% CI, 0.45-0.56; 17 cohorts) in asymptomatic patients.

This risk of long-term death after CEA in patients with symptomatic stenosis increased 26% (RR = 1.26, 95% CI, 1.20-1.32; 28 cohorts) with each 5-year more recent midyear of treatment, and increased 11% (RR = 1.11, 95% CI, 1.03-1.19; 24 cohorts) in cohorts with fewer than 500 patients. The risk of long-term stroke after CEA in patients with symptomatic stenosis did not increase significantly (RR = 1.04, 95% CI, 0.99-1.10; 28 cohorts) with each 5-year more recent midyear of treatment and decreased 13% (RR = 0.87, 95% CI, 0.80-0.94; 21 cohorts) in cohorts with fewer than 500 patients. Temporal trends after CEA could not be determined for the composite death or stroke and in patients with asymptomatic stenosis, as fewer than 20 cohorts were available (Table S8, Figure S1).

### Long-term outcomes after CAS

The annual incidence rate of long-term stroke or death after CAS was 4.67 (95% CI, 4.27-5.11; 18 cohorts) per 100 PY in symptomatic and 2.68 (95% CI, 2.34-3.06; 11 cohorts) in asymptomatic patients (Table 2). The annual incidence rates of long-term death and long-term stroke after CAS were 3.38 (95 % CI, 3.11-3.67; 26 cohorts) and 1.93 (95% CI, 1.75-2.13; 28 cohorts) in symptomatic, and 3.22 (95% CI, 2.99-3.46; 18 cohorts) and 1.05 (95% CI, 0.90-1.22; 19 cohorts) in asymptomatic patients. This risk of long-term death after CAS in patients with symptomatic stenosis did not decrease significantly (RR = 0.95, 95% CI, 0.87-1.03; 24 cohorts) with each 5-year more recent midyear of treatment, and decreased 33% (RR = 0.67, 95% CI, 0.59-0.76; 20 cohorts) in cohorts with fewer than 500 patients. The risk of stroke increased 30% (RR = 1.30, 95% CI, 1.17-1.43; 27 cohorts) with each 5-year more recent midyear of treatment and increased 44% (RR = 1.44, 95% CI, 1.28-1.62; 23 cohorts) in cohorts with fewer than 500 patients. Temporal trends after CAS could not be determined for the composite death or stroke and in patients with asymptomatic stenosis with fewer than 20 cohorts available (Table S9, Figure S2).

### Risk of bias

Most studies had low risk of selection bias, with only 10% including selected populations. Consecutive inclusion of patients was common, but not clearly reported in 53 (18%) cohorts. Baseline characteristics were frequently not stratified by intervention and/or symptomatic status in 150 (52%) cohorts. Data were retrospectively collected in 145 (50%) cohorts, 10 (4%) cohorts started with retrospective data collection and switched to prospective collection or did not specify their data collection. Reporting degree of stenosis according to the predefined reporting standard was done in 82 (28% of cohorts). A definition of stroke outcomes was not provided in 108 (37% cohorts) and outcome assessors were not specified in 149 (51%). Short-term follow-up was generally reported (259 cohorts, 89%). Long-term outcome was described in 72 cohorts, but in only 14 (19%) cohorts reported in patient-years or incidence rates. Loss to follow-up was not consistently described in 48 (68%) cohorts (Figure S3; Table S10).

## Discussion

In this meta-analysis of 475,266 patients undergoing CEA and 209,117 undergoing CAS, the 30-day risk of death or stroke after both interventions declined significantly over time for both symptomatic and asymptomatic stenosis. Similar trends were found in smaller studies (between 100 and 500 patients). Long-term death risk after CEA and long-term stroke risk after CAS increased over time.

The observed decrease in 30-day death or stroke risk after CEA and CAS may be attributed to better patient selection, improved medical therapy, stricter postoperative blood pressure control, increased experience and volume of the interventionalist and centralisation to high-volume centres.^21^ The confirmation of trends in smaller sample sizes suggests that the temporal trends are not solely driven by specialised centres or large registries.

We found higher short-term risk after CAS. Four large RCTs with an unbiased comparison of CEA with CAS in symptomatic patients also showed a higher risk of any stroke or death after CAS in the first 120 days after intervention, with an RR of 1.53 (95% CI, 1.20-1.95), especially when intervention was performed within 2 weeks after the index symptom.^22^ In the second asymptomatic carotid surgery trial that compared CEA with CAS in asymptomatic patients, the 30-day risk of non-disabling stroke was higher after CAS (*P* = .03), but no significant difference was found in any procedural stroke.^23^ The Sex, Contralateral Occlusion, Age and Restenosis risk score also helps to identify patients in whom CAS has a similar risk of perioperative death or stroke as CEA.^24^

Current Society for Vascular Surgery (SVS) and European SVS guidelines recommend performing CEA in symptomatic patients with a 30-day stroke or death risk below 6% and in asymptomatic patients with a risk below 3%.^25^^,^^26^ In the more recent 2021 European Stroke Organisation and the 2020 German–Austrian guidelines, an in-hospital stroke or death risk of 4% for symptomatic and 2% for asymptomatic patients is recommended.^27–29^ Whilst lower thresholds might be appropriate in the contemporary clinical setting with improved medical therapy, including more widespread use of antiplatelet agents and statins leading to lower stroke risks, with approximately one-third of 30-day procedural complications occurring after discharge, it means that the earlier thresholds are effectively preserved in newer guidelines.^30–33^ These thresholds might be useful as a benchmark for surgical quality at the centre or national level, ensuring that patients are not exposed to excessive operative risks. However, comparison of outcomes across centres and countries is hampered because case-mix differences that have a substantial impact on operative risks are not taken into account. Physicians might be helped by individualised risk prediction of procedural hazards. Previous risk prediction models have been validated in 26,293 patients of the NSQIP dataset and the OCER model that includes symptomatic status, diabetes, heart failure and contralateral occlusion showed reliable predictions of the 30-day death or stroke risk in symptomatic and asymptomatic patients. This could help focus CEA toward patients who benefit most from it.^34^ Other clinically relevant outcomes beyond death and stroke—including transient ischemic attacks, silent brain infarcts and perioperative myocardial infarction—also warrant consideration in this risk–benefit assessment, particularly in asymptomatic patients with a higher threshold for intervention.

A previous meta-analysis showed that the risk of recurrent stroke in patients with transient ischemic attack and ischaemic stroke did not decrease despite improved risk factor control.^35^ We found increased incidences of long-term death in symptomatic patients after CEA and long-term stroke in symptomatic patients after CAS over time, possibly with older-aged patients and increased comorbidity as contributing factors. A meta-analysis evaluating death rates in asymptomatic patients after CEA aligns with our findings, reporting a 5-year all-cause death rate of 22.7%. In this meta-analysis, most deaths in patients with asymptomatic carotid stenosis (after CEA or best medical treatment) were due to cardiac causes, mainly myocardial infarction and congestive heart failure.^36^ Current guidelines advise considering carotid interventions in asymptomatic patients if patient life expectancy exceeds 5 years.^25^^,^^29^

Our study indicates that patients remain at high risk of stroke and death after a carotid intervention and persistent risk factor control is crucial. A Canadian study reporting on adherence to statin therapy in patients who underwent CEA between 2002 and 2014 found reasonable adherence of 81.9% at 5-year follow-up.^37^ Levels of low-density lipid cholesterol and information on low- and high-intensity regiments were not available in this study and more recent data is lacking. Although our results indicate that CEA is an adequate intervention to prevent long-term stroke, the long-term risk of death is considerable and its increase over time emphasise the need for regular follow-up to maintain adherence in these patients. An individual patient data (IPD) meta-analysis might provide more insight in the observed upward trend in long-term mortality after CEA, as well as the increased risk of long-term stroke after CAS.

### Strengths and limitations

We used a comprehensive, unrestricted search with predefined selection criteria. Dual data extraction and rigorous appraisal minimised bias. However, missing data on variables like smoking status, sex and the presence of congestive heart failure have introduced potential confounding bias, as adjustment for these factors was not possible. Additionally, we could not stratify outcomes in symptomatic patients by symptom type or time from index event to intervention and the actual impact on the pooled effect estimates and regression analyses remains unclear. In several studies, baseline characteristics were reported only in aggregate, without stratification by type of intervention and symptomatic status. This limited our ability to adjust for important confounders such as age, sex, comorbidities, medication use and operator experience. These limitations, while common in aggregate data meta-analyses, underscore the need for an IPD meta-analysis. Second, outcome assessments were often non-standardised and not performed by independent neurologists, introducing variability and potential observer bias.^38^^,^^39^ Third, studies with varied medical regiments, stent types and surgical techniques (eg, longitudinal or eversion CEA) may have influenced outcomes. Fourth, among the 72 cohorts reporting long-term follow-up data, a fixed duration of follow-up was reported in only 19% of cohorts. We used the median or mean duration of follow-up in the other cohorts to calculate the total number of patient-years, which may have affected the incidence rate accuracy. Fifth, although we only included studies with ≥100 patients to minimise the risk of chance findings, this might hamper generalisability of our findings to hospitals and surgeons with even lower volumes. Sixth, even though we included studies from different countries and continents, the extent to which these results are applicable to different healthcare settings remains uncertain because outcomes were not analysed according to hospital type or healthcare system. Seventh, we did not evaluate the role of secondary prevention strategies, which may also have influenced long-term outcomes. Finally, despite efforts to exclude overlapping datasets, some overlap may persist in the meta-analysis, and we might have missed studies in which relevant outcomes were assessed but buried in a few words within the body of the text.

Our findings are important for clinical practice, in which patients and their treating physicians have to make decisions about carotid procedures. We showed that historic rates of procedural complications in pivotal trials have limited applicability in current practice. We have shown that the 30-day risk of death or stroke after CEA and CAS declined significantly over time in both patients with symptomatic and asymptomatic stenosis, with similar trends in smaller cohorts including 500 patients or fewer. The increased long-term risk of death after CEA and of stroke after CAS limits the durability of carotid interventions and warrants further scrutiny.

## Supplementary Material

aakaf002_Supplemental_Material_Final
