# Supplementary material for: Temporal trends in short- and long-term outcomes after carotid interventions for symptomatic or asymptomatic stenosis: a systematic review and meta-analysis
Source: Eur Stroke J. 2026 Jan 1;11(1):aakaf002. doi: 10.1093/esj/aakaf002 (PMC12866278; doi:10.1093/esj/aakaf002)
Supplement: aakaf002_Supplemental_Material_Final [file aakaf002_supplemental_material_final.docx]

**Temporal trends in short- and long-term outcomes after carotid interventions for symptomatic or asymptomatic stenosis: a systematic review and meta-analysis**

# Supplemental Material

## Table S1. PRISMA 2020 Checklist

| **Section and Topic** | **Item #** | **Checklist item** | **Location where item is reported** |
| --- | --- | --- | --- |
| **TITLE** | | |  |
| Title | 1 | Identify the report as a systematic review. | Page 1 |
| **ABSTRACT** | | |  |
| Abstract | 2 | See the PRISMA 2020 for Abstracts checklist. | Page 2 |
| **INTRODUCTION** | | |  |
| Rationale | 3 | Describe the rationale for the review in the context of existing knowledge. | Page 4 |
| Objectives | 4 | Provide an explicit statement of the objective(s) or question(s) the review addresses. | Page 4 |
| **METHODS** | | |  |
| Eligibility criteria | 5 | Specify the inclusion and exclusion criteria for the review and how studies were grouped for the syntheses. | Page 5 – 6 |
| Information sources | 6 | Specify all databases, registers, websites, organisations, reference lists and other sources searched or consulted to identify studies. Specify the date when each source was last searched or consulted. | Page 5 |
| Search strategy | 7 | Present the full search strategies for all databases, registers and websites, including any filters and limits used. | Page 5 – 6, Supplementary table 2 |
| Selection process | 8 | Specify the methods used to decide whether a study met the inclusion criteria of the review, including how many reviewers screened each record and each report retrieved, whether they worked independently, and if applicable, details of automation tools used in the process. | Page 5 |
| Data collection process | 9 | Specify the methods used to collect data from reports, including how many reviewers collected data from each report, whether they worked independently, any processes for obtaining or confirming data from study investigators, and if applicable, details of automation tools used in the process. | Page 5 – 6 |
| Data items | 10a | List and define all outcomes for which data were sought. Specify whether all results that were compatible with each outcome domain in each study were sought (e.g. for all measures, time points, analyses), and if not, the methods used to decide which results to collect. | Page 6 |
|  | 10b | List and define all other variables for which data were sought (e.g. participant and intervention characteristics, funding sources). Describe any assumptions made about any missing or unclear information. | Page 6 – 7 |
| Study risk of bias assessment | 11 | Specify the methods used to assess risk of bias in the included studies, including details of the tool(s) used, how many reviewers assessed each study and whether they worked independently, and if applicable, details of automation tools used in the process. | Page 6 – 7 |
| Effect measures | 12 | Specify for each outcome the effect measure(s) (e.g. risk ratio, mean difference) used in the synthesis or presentation of results. | Page 7 |
| Synthesis methods | 13a | Describe the processes used to decide which studies were eligible for each synthesis (e.g. tabulating the study intervention characteristics and comparing against the planned groups for each synthesis (item #5)). | Page 7 |
|  | 13b | Describe any methods required to prepare the data for presentation or synthesis, such as handling of missing summary statistics, or data conversions. | Page 7 |
|  | 13c | Describe any methods used to tabulate or visually display results of individual studies and syntheses. | Page 7 |
|  | 13d | Describe any methods used to synthesize results and provide a rationale for the choice(s). If meta-analysis was performed, describe the model(s), method(s) to identify the presence and extent of statistical heterogeneity, and software package(s) used. | Page 7 |
|  | 13e | Describe any methods used to explore possible causes of heterogeneity among study results (e.g. subgroup analysis, meta-regression). | NA |
|  | 13f | Describe any sensitivity analyses conducted to assess robustness of the synthesized results. | NA |
| Reporting bias assessment | 14 | Describe any methods used to assess risk of bias due to missing results in a synthesis (arising from reporting biases). | NA |
| Certainty assessment | 15 | Describe any methods used to assess certainty (or confidence) in the body of evidence for an outcome. | Page 7 |
| **RESULTS** | | |  |
| Study selection | 16a | Describe the results of the search and selection process, from the number of records identified in the search to the number of studies included in the review, ideally using a flow diagram. | Page 9 and Figure 1 |
|  | 16b | Cite studies that might appear to meet the inclusion criteria, but which were excluded, and explain why they were excluded. | Figure 1 and Supplementary table 5 |
| Study characteristics | 17 | Cite each included study and present its characteristics. | Page 8 |
| Risk of bias in studies | 18 | Present assessments of risk of bias for each included study. | Pages 10 Supplementary table 10, Supplementary figure 3 |
| Results of individual studies | 19 | For all outcomes, present, for each study: (a) summary statistics for each group (where appropriate) and (b) an effect estimate and its precision (e.g. confidence/credible interval), ideally using structured tables or plots. | Page 8 – 10 |
| Results of syntheses | 20a | For each synthesis, briefly summarise the characteristics and risk of bias among contributing studies. | Page 10 |
|  | 20b | Present results of all statistical syntheses conducted. If meta-analysis was done, present for each the summary estimate and its precision (e.g. confidence/credible interval) and measures of statistical heterogeneity. If comparing groups, describe the direction of the effect. | Page 8 – 10 |
|  | 20c | Present results of all investigations of possible causes of heterogeneity among study results. | NA |
|  | 20d | Present results of all sensitivity analyses conducted to assess the robustness of the synthesized results. | NA |
| Reporting biases | 21 | Present assessments of risk of bias due to missing results (arising from reporting biases) for each synthesis assessed. | NA |
| Certainty of evidence | 22 | Present assessments of certainty (or confidence) in the body of evidence for each outcome assessed. | Page 10 |
| **DISCUSSION** | | |  |
| Discussion | 23a | Provide a general interpretation of the results in the context of other evidence. | Page 11 |
|  | 23b | Discuss any limitations of the evidence included in the review. | Page 13 – 14 |
|  | 23c | Discuss any limitations of the review processes used. | Page 13 – 14 |
|  | 23d | Discuss implications of the results for practice, policy, and future research. | Page 13 – 14 |
| **OTHER INFORMATION** | | |  |
| Registration and protocol | 24a | Provide registration information for the review, including register name and registration number, or state that the review was not registered. | Page 5 |
|  | 24b | Indicate where the review protocol can be accessed, or state that a protocol was not prepared. | Page 5 |
|  | 24c | Describe and explain any amendments to information provided at registration or in the protocol. | Page 5 |
| Support | 25 | Describe sources of financial or non-financial support for the review, and the role of the funders or sponsors in the review. | Page 15 |
| Competing interests | 26 | Declare any competing interests of review authors. | Page 15 |
| Availability of data, code and other materials | 27 | Report which of the following are publicly available and where they can be found: template data collection forms; data extracted from included studies; data used for all analyses; analytic code; any other materials used in the review. | NA |

## Table S2. Search strategy

| *MEDLINE via PubMed (18/11/2024: 5883 results)* |
| --- |
| 1. “Carotid stenosis”[Mesh] OR “Carotid Artery Thrombosis”[Mesh] OR “Carotid Arteries”[Mesh] OR ‘Carotid artery diseases’[Mesh] OR "Carotid stenos*"[Title/Abstract] OR "Carotid Artery Thrombos*"[Title/Abstract] OR "Carotid artery diseas*"[Title/Abstract] |
| 1. "Endarterectomy, Carotid"[Mesh] OR Carotid endart*[Title/Abstract] OR endovascular*[Title/abstract] OR “CEA”[Title/Abstract] OR Carotid stent*[Title/Abstract] OR Carotid artery stent*[Title/Abstract] OR “CAS”[Title/Abstract] OR “Endovascular Procedures”[Mesh] |
| 1. “peri-procedur*”[Title/abstract] OR peri procedur*[Title/abstract] OR periprocedur*[Title/abstract] OR peri-operat*[Title/abstract] OR peri operat*[Title/abstract] OR perioperat*[Title/abstract] OR “postoperat*”[Title/abstract] OR “Long term adverse effects”[Mesh] OR “Long term”[Title/abstract] OR “Long-term”[Title/abstract] OR “month long”[Title/abstract] OR “month-long”[Title/abstract] OR “year-long”[Title/abstract] OR “yearly”[Title/abstract] OR thirty-day*[Title/abstract] OR 30-day*[Title/abstract] OR thirty day*[Title/abstract] OR “over the years”[Title/abstract] OR 1-year*[Title/abstract] OR 1 year*[Title/abstract] OR one-year* [Title/abstract] OR 2-year*[Title/abstract] OR 2 year*[Title/abstract] OR two-year* [Title/abstract] OR 3-year*[Title/abstract] OR 3 year*[Title/abstract] OR three-year* [Title/abstract] OR 4-year* [Title/abstract] OR 4 year*[Title/abstract] OR four-year* [Title/abstract] OR 5-year*[Title/abstract] OR 5 year*[Title/abstract] OR five-year* [Title/abstract] OR 6-year*[Title/abstract] OR 6 year* [Title/abstract] OR six-year*[Title/abstract] OR 7-year*[Title/abstract] OR 7 year*[Title/abstract] OR seven-year*[Title/abstract] OR 8-year*[Title/abstract] OR 8 year*[Title/abstract] OR eight-year* [Title/abstract] OR 9-year*[Title/abstract] OR 9 year*[Title/abstract] OR nine-year* [Title/abstract] OR 10-year*[Title/abstract] OR 10 year*[Title/abstract] OR ten-year* [Title/abstract] |
| 1. “transient ischemic attack*”[Title/abstract] OR “TIA”[Title/abstract] OR “survival” [Title/abstract] OR “Stroke”[Mesh] OR myocard*[Title/abstract] OR “Stroke*”[Title/abstract] OR “Ischemic Attack, Transient”[Mesh] OR “Myocardial infarction”[Mesh] OR myocard* [Title/abstract] OR “MI”[Title/abstract] OR mortalit*[Title/abstract] OR death*[Title/abstract] OR “neurological symptom*”[Title/abstract] OR complication*[Title/abstract] OR adverse event* [Title/abstract] OR adverse effect*[Title/abstract] 2. #1 AND #2 AND #3 AND #4 |
| *EMBASE (18/11/2024: 539 results)* |
| 1. (‘Carotid artery occlusion’/exp OR ‘external carotid artery’/exp OR ‘internal carotid artery’/exp OR ‘right common carotid artery’/exp OR ‘carotid artery thrombos*’:ti,ab,kw OR ‘carotid stenos*’:ti,ab,kw OR ‘carotid artery diseas*’:ti,ab,kw) AND [embase]/lim NOT ([embase]/lim AND [medline]/lim) 2. (‘carotid artery stent’/exp OR ‘carotid artery surgery’/exp OR   ‘CEA’:ti,ab,kw OR ‘carotid endart*’:ti,ab,kw OR ‘carotid stent*’:ti,ab,kw OR ‘carotid artery stent*’:ti,ab,kw OR ‘CAS’:ti,ab,kw OR ‘endovascular’:ti,ab,kw OR ‘endart*’:ti,ab,kw OR ‘carotid surger*’:ti,ab,kw OR ‘carotid revascularization*’:ti,ab,kw OR ‘carotid revascularisation*’:ti,ab,kw) AND [embase]/lim NOT ([embase]/lim AND [medline]/lim) |
| 1. (‘peri-procedur*’:ti,ab,kw OR peri procedur*:ti,ab,kw OR periprocedur*:ti,ab,kw OR peri-operat*:ti,ab,kw OR peri operat*:ti,ab,kw OR perioperat*:ti,ab,kw OR ‘postoperat*’:ti,ab,kw OR ‘Long term’:ti,ab,kw OR ‘Long-term’:ti,ab,kw OR ‘month long’:ti,ab,kw OR ‘month-long’:ti,ab,kw OR ‘year-long’:ti,ab,kw OR ‘yearly’:ti,ab,kw OR thirty-day*:ti,ab,kw OR 30-day*:ti,ab,kw OR thirty day*:ti,ab,kw OR ‘over the years’:ti,ab,kw OR 1-year*:ti,ab,kw OR 1 year*:ti,ab,kw OR one-year*:ti,ab,kw OR 2-year*:ti,ab,kw OR 2 year*:ti,ab,kw OR two-year*:ti,ab,kw OR 3-year*:ti,ab,kw OR 3 year*:ti,ab,kw OR three-year*:ti,ab,kw OR 4-year*:ti,ab,kw OR 4 year*:ti,ab,kw OR four-year*:ti,ab,kw OR 5-year*:ti,ab,kw OR 5 year*:ti,ab,kw OR five-year*:ti,ab,kw OR 6-year*:ti,ab,kw OR 6 year*:ti,ab,kw OR six-year*:ti,ab,kw OR 7-year*:ti,ab,kw OR 7 year*:ti,ab,kw OR seven-year*:ti,ab,kw OR 8-year*:ti,ab,kw OR 8 year*:ti,ab,kw OR eight-year*:ti,ab,kw OR 9-year*:ti,ab,kw OR 9 year*:ti,ab,kw OR nine-year*:ti,ab,kw OR 10-year*:ti,ab,kw OR 10 year*:ti,ab,kw OR ten-year*:ti,ab,kw) AND [embase]/lim NOT ([embase]/lim AND [medline]/lim) |
| 1. (‘Transient ischemic attack’/exp OR ‘TIA’:ti,ab,kw OR ‘transient ischemic attack*’:ti,ab,kw OR ‘stroke*’:ti,ab,kw OR ‘heart infarction’/exp OR ‘heart infarction*’:ti,ab,kw OR ‘myocardial infarction*’:ti,ab,kw OR ‘mortality’:ti,ab,kw OR ‘death*’:ti,ab,kw OR ‘neurological symptom*’:ti,ab,kw OR ‘survival’:ti,ab,kw OR complication*:ti,ab,kw OR adverse event*:ti,ab,kw OR adverse effect*) AND [embase]/lim NOT ([embase]/lim AND [medline]/lim) 2. #1 AND #2 AND #3 AND #4 |

## Table S3. Criteria for the risk of bias assessment

| **Risk of bias items** | **Risk of bias** | | |  |
| --- | --- | --- | --- | --- |
|  | **High** | **Intermediate** | **Low** | **Unclear** |
| 1. Patient selection | Restriction of inclusion to high-risk patients* | Some selection criteria of patients was were applied by age, sex | Representative cohort of patients undergoing carotid revascularization | Inclusion criteria not provided |
| 2. Consecutiveness | No consecutive series or random sampling | - | Consecutive series or random sampling† | Not reported |
| 3. Baseline characteristics | Baseline characteristics (sex, age, type of index event) not provided per cohort included in meta-analysis |  | Baseline characteristics (sex, age, type of index event) provided per cohort included in meta-analysis |  |
| 4. Data collection | Retrospective data collection (at least partly) |  | Prospective data collection‡ |  |
| 5. Degree of stenosis measurement | Patients not classified according to degree of stenosis. | Stenosis degree cut-offs defined, but inconsistent with reporting standards (NASCET & ECST measurement) | Patients classified according to reporting standards (NASCET measurement; 50-69% for moderate stenosis, 70-99% for severe stenosis) |  |
| 6. Definition of outcomes | Stroke outcome definition not provided | - | Stroke outcome definitions provided |  |
| 7. Outcome ascertainment | Outcomes were not ascertained by a neurologist or endpoint adjudication committee | Reference to ‘neurologic examination’ or use of NIHSS scale without explicit mention of ascertainment by a neurologist OR outcome ascertainment by a neurologist for some patients, but not all patients. | All outcomes were ascertained by a neurologist or endpoint adjudication committee |  |
| 8a. Short-term follow-up definition | Timeframe of outcome not clearly described (procedural, periprocedural, postprocedural) | Timeframe of outcome defined but the duration could vary between patients (in-hospital) | Timeframe of outcome clearly described and the same for all patients (30-days) |  |
| 8b. Long-term follow-up definition | Long-term outcomes reported with mean or median duration of follow-up | - | Long-term outcomes reported with specification of patient-years or incidence rates |  |
| 9. Loss to follow-up | Loss to follow-up for long-term outcomes >5% | - | Loss to follow-up for long-term outcomes ≤5% | Not reported |
| * High-risk was defined as octogenarians (patients >80 years), or presence of one or more physiological (e.g. CHF class III/IV, ESRD, left ventricular ejection fraction <30%, unstable angina, recent MI) or anatomical (e.g. contralateral carotid occlusion, prior CEA/CAS with recurrent stenosis**, prior neck radiation**) high-risk characteristics.  ** This characteristic individually must be present in <10% of the patient cohort, else the cohort is excluded.  † Including studies reporting that ‘all patients undergoing CEA’ were included.  ‡ Retrospective analysis of prospectively collected data is also considered ‘low risk of bias’.  CAS: carotid artery stenting; CEA: carotid endarterectomy; ECST: European Carotid Surgery Trial; NASCET: North American Symptomatic Carotid Endarterectomy Trial; NIHSS: National Institute of Health Stroke Scale. | | | | |

## Table S4. Summary of study-level characteristics

|  | **CEA** | | | **CAS** | | |
| --- | --- | --- | --- | --- | --- | --- |
| **Characteristics** | **No. of cohort reported (%)** | **Patients** | **Median across studies (IQR)** | **No. of cohort reported (%)** | **Patients** | **Median across studies (IQR)** |
| Number of patients* | 239 (100) | 475,266 | 288 (128-585) | 227 (100) | 209,117 | 182 (88-460) |
| Midyear of treatment | 227 (95.0) | 472,855 | 2006 (2000-2012 | 218 (96.0) | 208,026 | 2008 (2003-2012) |
| Age | 89 (37.2) | 95,107 | 70 (69-72) | 65 (28.6) | 26,636 | 70 (68-72) |
| Male sex | 102 (42.7) | 105,607 | 69 (65-73) | 76 (33.5) | 96,929 | 73 (65-79) |
| Current smoker | 57 (23.8) | 56,760 | 33 (25-47) | 58 (25.6) | 15,073 | 29 (24-44) |
| Diabetes mellitus | 86 (36.0) | 95,128 | 27 (22-32) | 71 (31.3) | 19,939 | 32 (27-38) |
| Heart failure | 20 (8.4) | 38,823 | 5 (2-9) | 14 (6.2) | 4064 | 9 (4-13) |
| Contralateral occlusion | 37 (15.5) | 37,397 | 6 (4-10) | 31 (13.7) | 12,811 | 9 (6-12) |
| Symptomatic status | 239 (100) | 475,266 | † | 227 (100) | 209,117 | † |
| Asymptomatic stenosis | 101 (42.3) | 260,740 | † | 109 (48.0) | 131,984 | † |
| Symptomatic stenosis | 138 (57.5) | 214,526 | † | 118 (52.0) | 77,133 | † |
| *Qualifying event stroke* | 77 (55.8) | 168,078 | 44 (34-56) | 57 (48.3) | 59,727 | 37 (5-66) |
| *Qualifying event TIA* | 76 (55.1) | 168,369 | 40 (30-50) | 55 (46.6) | 58,182 | 32 (0-47) |
| *Qualifying event ocular* | 77 (55.8) | 173,940 | 7 (0-18) | 53 (44.9) | 57,967 | 2 (0-15) |
| Degree of stenosis |  |  |  |  |  |  |
| *Moderate stenosis ipsilateral* | 71 (29.7) | 85,365 | 9 (0-26) | 46 (20.3) | 23,301 | 0 (0-25) |
| *Severe stenosis ipsilateral* | 68 (28.5) | 85,104 | 89 (73-100) | 43 (18.9) | 22,525 | 97 (70-100) |
| *Near occlusion ipsilateral* | 17 (7.1) | 27,188 | 0 (0-0) | 14 (6.2) | 5023 | 0 (0-8) |
| * We used the largest cohorts to describe the baseline characteristics if different outcomes of the same cohort or the subset of a cohort were reported in multiple publications. This occurred in 35 cohorts. † Cohorts were stratified by symptomatic status (symptomatic vs. asymptomatic stenosis).  CAS: carotid artery stenting; CEA: carotid endarterectomy; IQR: interquartile range; TIA = transient ischemic attack | | | | | | |

## Table S5. Full-text evaluation

| Reason for exclusion of studies (n = 1793) |
| --- |
| 1. Conference abstract (n = 65) |
| 2. Degree of stenosis not provided or >10% degree of stenosis <50% (n = 166) |
| 3. Duplicate study (n = 51) |
| 4. Full-text not available (n = 160) |
| 5. Incidence rate not provided and not possible to calculate with data provided (n = 64) |
| 6. Less than 100 patients with extracranial carotid stenosis included (n = 26) |
| 7. No original treatment data (n = 10) |
| 8. Outcomes not provided for CEA and CAS separately (n = 24) |
| 9. Outcomes not provided for symptomatic and asymptomatic patients separately (n = 809) |
| 10. Overlapping data (n = 106) |
| 10. Predefined outcomes not provided (n = 63) |
| 11. Studies with >10% bilateral interventions (n = 169) |
| 12. Studies with >10% re-do interventions (n = 30) |
| 12. Studies with >10% synchronous interventions (e.g. CABG) (n = 9) |
| 13. Studies with >10% radiation induced carotid stenosis (n = 1) |
| 14. Symptomatic group not defined as symptoms within 6 months prior (n = 9) |
| 15. Timeframe short-term complications not defined (n = 31) |
| **Included studies (N = 291*)** |
| *See Supplemental Table 9 for a full overview of included articles  CABG: coronary artery bypass grafting; CAS: carotid artery stenting; CEA: carotid endarterectomy. |

## Table S6. Time trends in short-term outcomes after carotid endarterectomy

|  | **RR midyear (per 5 years increase)** | **N of cohorts** | **RR midyear (per 5 years increase) in cohorts <500 patients** | **N of cohorts** |
| --- | --- | --- | --- | --- |
| **Symptomatic patients** | | | | |
| **Death or stroke** |  |  |  |  |
| *30-days* | 0.64 (95% CI 0.63-0.64) | 85 | 0.79 (95% CI 0.78-0.81) | 63 |
| *In-hospital** | *NA* | *NA* | *NA* | *NA* |
| **Death** |  |  |  |  |
| *30-days* | 0.91 (95% CI 0.91-0.92) | 95 | 0.90 (95% CI 0.89-0.90) | 74 |
| *In-hospital** | *NA* | *NA* | *NA* | *NA* |
| **Stroke** |  |  |  |  |
| *30-days* | 0.74 (95% CI 0.73-0.74) | 100 | 0.79 (95% CI 0.78-0.80) | 77 |
| *In-hospital** | *NA* | *NA* | *NA* | *NA* |
| **Asymptomatic patients** | | | | |
| **Death or stroke** |  |  |  |  |
| *30-days* | 0.59 (95% CI 0.59-0.59) | 61 | 0.74 (95% CI 0.73-0.76) | 42 |
| *In-hospital** | *NA* | *NA* | *NA* | *NA* |
| **Death** |  |  |  |  |
| *30-days* | 0.82 (95% CI 0.82-0.82) | 67 | 0.82 (95% CI 0.81-0.83) | 46 |
| *In-hospital** | *NA* | *NA* | *NA* | *NA* |
| **Stroke** |  |  |  |  |
| *30-days* | 0.71 (95% CI 0.71-0.71) | 69 | 0.80 (95% CI 0.79-0.81) | 47 |
| *In-hospital** | *NA* | *NA* | *NA* | *NA* |
| *Fewer than 20 cohorts reported this in-hospital outcome. | | | | |
| NA: not applicable; RR: rate ratio. | | | | |

## Table S7. Time trends in short-term outcomes after carotid artery stenting

|  | **RR midyear (per 5 years increase)** | **N of cohorts** | **RR midyear (per 5 years increase) in cohorts <500 patients** | **N of cohorts** |
| --- | --- | --- | --- | --- |
| **Symptomatic patients** | | | | |
| **Death or stroke** |  |  |  |  |
| *30-days* | 0.56 (95% CI 0.53-0.58) | 71 | 0.51 (95% CI 0.47-0.55) | 62 |
| *In-hospital** | *NA* | *NA* | *NA* | *NA* |
| **Death** |  |  |  |  |
| *30-days* | 0.87 (95% CI 0.86-0.88) | 81 | 0.97 (95% CI 0.95-1.00) | 69 |
| *In-hospital** | *NA* | *NA* | *NA* | *NA* |
| **Stroke** |  |  |  |  |
| *30-days* | 0.48 (95% CI 0.47-0.50) | 86 | 1.00 (95% CI 0.92-1.09) | 74 |
| *In-hospital** | *NA* | *NA* | *NA* | *NA* |
| **Asymptomatic patients** | | | | |
| **Death or stroke** |  |  |  |  |
| *30-days* | 0.73 (95% CI 0.71-0.74) | 62 | 0.56 (95% CI 0.54-0.58) | 46 |
| *In-hospital** | *NA* | *NA* | *NA* | *NA* |
| **Death** |  |  |  |  |
| *30-days* | 0.84 (95% CI 0.84-0.85) | 69 | 0.84 (95% CI 0.83-0.86) | 53 |
| *In-hospital** | *NA* | *NA* | *NA* | *NA* |
| **Stroke** |  |  |  |  |
| *30-days* | 0.65 (95% CI 0.64-0.65) | 73 | 0.70 (95% CI 0.68-0.72) | 58 |
| *In-hospital** | *NA* | *NA* | *NA* | *NA* |
| *Fewer than 20 cohorts reported this in-hospital outcome | | | | |
| NA: not applicable; RR: rate ratio. | | | | |

## Table S8. Time trends in long-term outcomes after carotid endarterectomy

|  | **RR midyear (per 5 years increase)** | **N of cohorts** | **RR midyear (per 5 years increase) in cohorts <500 patients** | **N of cohorts** |
| --- | --- | --- | --- | --- |
|  | **Symptomatic patients** | | | |
| **Death or stroke*** | *NA* | *NA* | *NA* | *NA* |
| **Death** | 1.26 (95% CI 1.20-1.32)† | 28 | 1.11 (95% CI 1.03-1.19) | 24 |
| **Stroke** | 1.04 (95% CI 0.99-1.10)‡ | 28 | 0.87 (95% CI 0.80-0.94) | 21 |
|  | **Asymptomatic patients** | | | |
| **Death or stroke*** | *NA* | *NA* | *NA* | *NA* |
| **Death*** | *NA* | *NA* | *NA* | *NA* |
| **Stroke*** | *NA* | *NA* | *NA* | *NA* |
| *Fewer than 20 cohorts reported this outcome. † The rate ratio was 1.15 (95% CI 1.06-1.25) in 20 cohorts after adjustment for cohort-level age and sex. ‡ The rate ratio was 1.25 (95% CI 1.10-1.42) in 20 cohorts after adjustment for cohort-level age and sex. | | | | |
| NA: not applicable; RR: rate ratio. | | | | |

## Table S9. Time trends in long-term outcomes after carotid artery stenting

|  | **RR midyear (per 5 years increase)** | **N of cohorts** | **RR midyear (per 5 years increase) in cohorts <500 patients** | **N of cohorts** |
| --- | --- | --- | --- | --- |
|  | **Symptomatic patients** | | | |
| **Death or stroke*** | *NA* | *NA* | *NA* | *NA* |
| **Death** | 0.95 (95% CI 0.87-1.03)† | 24 | 0.67 (95% CI 0.59-0.76)† | 20 |
| **Stroke** | 1.30 (95% CI 1.17-1.43)† | 27 | 1.44 (95% CI 1.28-1.62)† | 23 |
|  | **Asymptomatic patients** | | | |
| **Death or stroke*** | *NA* | *NA* | *NA* | *NA* |
| **Death*** | *NA* | *NA* | *NA* | *NA* |
| **Stroke*** | *NA* | *NA* | *NA* | *NA* |
| *Fewer than 20 cohorts reported this outcome. † With fewer than 20 cohorts available, we did not adjust for cohort-level age and sex | | | | |
| NA: not applicable; RR: rate ratio. | | | | |

## Table S10. Risk of bias assessment for articles with symptomatic patients undergoing carotid endarterectomy

| **First author** | **Year of publication** | **Symptomatic status** | **Intervention** | **1. Patient selection** | **2. Consecutiveness** | **3. Baseline characteristics** | **4. Prospective data collection** | **5. Degree of stenosis** | **6. Outcome definition** | **7. Outcome ascertainment** | **8a. Short-term FU definition** | **8b. Long-term FU definition** | **9. Loss to FU** |  |
| --- | --- | --- | --- | --- | --- | --- | --- | --- | --- | --- | --- | --- | --- | --- |
| AbuRahma^1^ | 2014 | ASx | CEA | Low | Low | High | Low | High | High | Int | Low | NA | NA |  |
| AbuRahma^1^ | 2014 | Sx | CEA | Low | Low | High | Low | High | High | Int | Low | NA | NA |  |
| AbuRahma^2^ | 2017 | ASx | CAS | Low | Low | High | Low | High | Low | Low | Low | NA | NA |  |
| AbuRahma^2^ | 2017 | Sx | CAS | Low | Low | High | Low | High | Low | Low | Low | NA | NA |  |
| Achim^3^ | 2022 | ASx | CAS | Low | Low | High | Low | High | Low | High | Low | NA | NA |  |
| Adelman^4^ | 1995 | Sx | CEA | High | Unclear | High | Unclear | High | High | High | Low | NA | NA |  |
| Adelman^4^ | 1995 | ASx | CEA | High | Unclear | High | Unclear | High | High | High | Low | NA | NA |  |
| Akkan^7^ | 2018 | Sx | CAS | High | Low | High | High | High | Low | Low | Low | NA | NA |  |
| Akkan^7^ | 2018 | ASx | CAS | High | Low | High | High | High | Low | Low | Low | NA | NA |  |
| Alcalde^6^ | 2018 | Sx | CAS | Low | Low | Low | Low | Low | Low | Low | Low | NA | NA |  |
| Almekhlafi^8^ | 2011 | Sx | CAS | Low | Low | Low | High | Low | Low | Low | Low | NA | NA |  |
| Alvarez^9^ | 2021 | Sx | CEA | Low | Low | High | High | High | High | Int | Int | High | Unclear |  |
| Alvarez^9^ | 2021 | ASx | CEA | Low | Low | High | High | High | High | Int | Int | High | Unclear |  |
| Angle^5^ | 2022 | Sx | CEA | Low | Low | Low | High | Int | Low | Low | Low | NA | NA |  |
| Angle^5^ | 2022 | Sx | CEA | Low | Low | Low | High | Int | Low | Low | Low | NA | NA |  |
| Angle^5^ | 2022 | ASx | CEA | Low | Low | Low | High | Int | Low | Low | Low | NA | NA |  |
| Angle^5^ | 2022 | ASx | CEA | Low | Low | Low | High | Int | Low | Low | Low | NA | NA |  |
| Annambhotla^10^ | 2012 | Sx | CEA | Low | Low | Low | High | High | Low | Int | Low | Low | Unclear |  |
| Ansel^11^ | 2010 | Sx | CAS | High | High | High | Low | High | High | Low | Low | NA | NA |  |
| Ansel^11^ | 2010 | ASx | CAS | High | High | High | Low | High | High | Low | Low | NA | NA |  |
| Appleberg^12^ | 1995 | ASx | CEA | Low | Low | Low | High | Low | High | High | Low | High | High |  |
| Arhuidese^13^ | 2018 | Sx | CEA | Low | Low | High | Low | High | High | High | Low | High | Unclear |  |
| Arhuidese^13^ | 2018 | ASx | CEA | Low | Low | High | Low | High | High | High | Low | High | Unclear |  |
| Arhuidese^14^ | 2017 | Sx | CAS | Low | Low | Low | High | Low | High | High | Low | NA | NA |  |
| Arhuidese^14^ | 2017 | ASx | CAS | Low | Low | Low | High | Low | High | High | Low | NA | NA |  |
| Arslan^15^ | 2014 | Sx | CAS | Low | Unclear | High | Low | High | High | Low | Int | High | Unclear |  |
| Arslan^15^ | 2014 | ASx | CAS | Low | Unclear | High | Low | High | High | Low | Int | High | Unclear |  |
| Ascher^16^ | 2004 | Sx | CEA | Low | Low | High | High | High | High | High | Low | NA | NA |  |
| Ascher^16^ | 2004 | ASx | CEA | Low | Low | High | High | High | High | High | Low | NA | NA |  |
| Avgerinos^17^ | 2015 | Sx | CEA | Low | Low | High | High | High | Low | High | Low | NA | NA |  |
| Avgerinos^17^ | 2015 | ASx | CEA | Low | Low | High | High | High | Low | High | Low | NA | NA |  |
| Baker^18^ | 2000 | ASx | CEA | Low | Unclear | Low | Low | High | High | High | Low | NA | NA |  |
| Baker^19^ | 2023 | Sx | CEA | Low | Low | High | Low | High | High | High | Low | NA | NA |  |
| Ballotta^20^ | 2003 | Sx | CEA | Low | High | High | High | High | Low | Low | Low | NA | NA |  |
| Ballotta^20^ | 2003 | ASx | CEA | Low | High | High | High | High | Low | Low | Low | NA | NA |  |
| Ballotta^21^ | 2008 | Sx | CEA | Low | Low | Low | Low | Int | Low | Low | Low | High | Low |  |
| Banga^22^ | 2018 | Sx | CEA | Low | High | High | Low | High | Low | Low | Int | NA | NA |  |
| Banga^22^ | 2018 | ASx | CEA | Low | High | High | Low | High | Low | Low | Int | NA | NA |  |
| Baram^25^ | 2022 | Sx | CEA | Low | Low | Low | Low | High | High | High | Low | High | Unclear |  |
| Barbetta^27^ | 2014 | Sx | CEA | Low | Low | Low | High | High | Low | Int | Low | NA | NA |  |
| Barnett^23^ | 1991 | Sx | CEA | Low | Low | Low | Low | Low | Low | Low | Low | NA | NA |  |
| Barnett^26^ | 1998 | Sx | CEA | Low | Low | Low | Low | Low | Low | Low | NA | High | Low |  |
| Bazan^24^ | 2014 | Sx | CEA | Low | Low | High | Low | High | Low | High | Low | NA | NA |  |
| Becquemin^28^ | 2003 | Sx | CAS | Low | Low | High | Low | Low | Low | Int | Low | NA | NA |  |
| Becquemin^28^ | 2003 | ASx | CAS | Low | Low | High | Low | Low | Low | Int | Low | NA | NA |  |
| Ben Ahmed^29^ | 2017 | Sx | CEA | Low | Low | High | High | Low | High | High | Low | NA | NA |  |
| Bibl^31^ | 2005 | Sx | CAS | Low | Low | High | High | Low | High | Low | Low | High | High |  |
| Biggs^32^ | 2014 | Sx | CEA | Low | Low | High | Low | High | Low | Low | Low | NA | NA |  |
| Biggs^32^ | 2014 | ASx | CEA | Low | Low | High | Low | High | Low | Low | Low | NA | NA |  |
| Bilas^30^ | 2003 | Sx | CEA | Low | Low | High | High | High | High | Int | Low | NA | NA |  |
| Bilas^30^ | 2003 | ASx | CEA | Low | Low | High | High | High | High | Int | Low | NA | NA |  |
| Binning^33^ | 2017 | Sx | CAS | Low | Low | High | High | High | High | High | Low | NA | NA |  |
| Binning^33^ | 2017 | ASx | CAS | Low | Low | High | High | High | High | High | Low | NA | NA |  |
| Bissacco^34^ | 2018 | ASx | CEA | Low | Low | Low | High | Low | Low | Low | Low | NA | NA |  |
| Blohmé^35^ | 1999 | Sx | CEA | Low | Low | Low | High | Low | Low | Low | Low | NA | NA |  |
| Boitano^36^ | 2020 | Sx | CEA | Low | Unclear | High | High | Int | High | High | Low | NA | NA |  |
| Bonati^37^ | 2015 | Sx | CEA | Low | Low | Low | Low | Low | Low | Low | NA | High | Unclear |  |
| Bonati^37^ | 2015 | Sx | CAS | Low | Low | Low | Low | Low | Low | Low | NA | High | Unclear |  |
| Bosiers^38^ | 2005 | Sx | CEA | Low | Low | High | High | High | High | High | Low | NA | NA |  |
| Bosiers^38^ | 2005 | ASx | CEA | Low | Low | High | High | High | High | High | Low | NA | NA |  |
| Bosiers^38^ | 2005 | Sx | CAS | Low | Low | High | High | High | High | High | Low | NA | NA |  |
| Bosiers^38^ | 2005 | ASx | CAS | Low | Low | High | High | High | High | High | Low | NA | NA |  |
| Bosiers^39^ | 2015 | Sx | CAS | High | High | High | Low | Int | Low | Low | Low | NA | NA |  |
| Bosiers^39^ | 2015 | ASx | CAS | High | High | High | Low | Int | Low | Low | Low | NA | NA |  |
| Bourke^40^ | 2016 | Sx | CEA | Low | Low | Low | Low | High | Low | High | Low | NA | NA |  |
| Bourke^40^ | 2016 | ASx | CEA | Low | Low | Low | Low | High | Low | High | Low | NA | NA |  |
| Bramucci^41^ | 2023 | Sx | CAS | High | Low | High | High | Int | Low | High | Low | High | Unclear |  |
| Bramucci^41^ | 2023 | ASx | CAS | High | Low | High | High | Int | Low | High | Low | High | Unclear |  |
| Branchereau^42^ | 1998 | ASx | CEA | Low | Low | Low | High | High | High | High | Low | Low | Low |  |
| Brott^43^ | 2010 | Sx | CEA | Low | Low | High | Low | High | Low | Low | Low | NA | NA |  |
| Brott^43^ | 2010 | ASx | CEA | Low | Low | High | Low | High | Low | Low | Low | NA | NA |  |
| Brott^43^ | 2010 | Sx | CAS | Low | Low | High | Low | High | Low | Low | Low | NA | NA |  |
| Brott^43^ | 2010 | ASx | CAS | Low | Low | High | Low | High | Low | Low | Low | NA | NA |  |
| Brown^44^ | 2008 | Sx | CEA | Low | Low | High | High | High | Low | Low | Low | NA | NA |  |
| Brown^44^ | 2008 | ASx | CEA | Low | Low | High | High | High | Low | Low | Low | NA | NA |  |
| Brown^44^ | 2008 | Sx | CAS | Low | Low | High | High | High | Low | Low | Low | NA | NA |  |
| Brown^44^ | 2008 | ASx | CAS | Low | Low | High | High | High | Low | Low | Low | NA | NA |  |
| Cacioppa^45^ | 2018 | Sx | CEA | Low | Low | High | High | High | Low | Low | Low | NA | NA |  |
| Calo^278^ | 2023 | Sx | CAS | Low | Low | High | High | Int | Low | Low | Int | NA | NA |  |
| Calo^278^ | 2023 | ASx | CAS | Low | Low | High | High | Int | Low | Low | Int | NA | NA |  |
| Calvillo^46^ | 2010 | ASx | CEA | Low | Low | Low | High | Low | High | Low | Low | NA | NA |  |
| Casana^62^ | 2020 | Sx | CAS | Low | Low | High | High | High | High | High | Low | High | High |  |
| Casana^63^ | 2020 | Sx | CAS | Low | Low | High | High | High | High | High | Low | NA | NA |  |
| Casana^63^ | 2020 | ASx | CAS | Low | Low | High | High | High | High | High | Low | NA | NA |  |
| Castro-Afonso^61^ | 2015 | Sx | CAS | Low | Low | Low | High | Int | Low | Low | Low | High | Low |  |
| Castro-Afonso^61^ | 2015 | ASx | CAS | Low | Low | Low | High | Int | Low | Low | Low | High | Low |  |
| Cernetti^60^ | 2003 | Sx | CAS | Low | Low | High | Low | Low | Low | Low | Low | NA | NA |  |
| Cernetti^60^ | 2003 | Sx | CAS | Low | Low | High | Low | Low | Low | Low | Low | NA | NA |  |
| Cernetti^60^ | 2003 | ASx | CAS | Low | Low | High | Low | Low | Low | Low | Low | NA | NA |  |
| Cernetti^60^ | 2003 | ASx | CAS | Low | Low | High | Low | Low | Low | Low | Low | NA | NA |  |
| Charalampoudis^59^ | 2011 | Sx | CEA | Low | Low | High | High | High | High | High | Low | NA | NA |  |
| Charalampoudis^59^ | 2011 | ASx | CEA | Low | Low | High | High | High | High | High | Low | NA | NA |  |
| Chaturvedi^58^ | 2010 | Sx | CAS | High | Low | High | Low | High | Low | Low | Low | NA | NA |  |
| Chaturvedi^58^ | 2010 | ASx | CAS | High | Low | High | Low | High | Low | Low | Low | NA | NA |  |
| Chiam^57^ | 2008 | Sx | CAS | Low | Low | High | Low | High | Low | Low | Low | NA | NA |  |
| Chiam^57^ | 2008 | Sx | CAS | Low | Low | High | Low | High | Low | Low | Low | NA | NA |  |
| Chiam^57^ | 2008 | ASx | CAS | Low | Low | High | Low | High | Low | Low | Low | NA | NA |  |
| Chiam^57^ | 2008 | ASx | CAS | Low | Low | High | Low | High | Low | Low | Low | NA | NA |  |
| Chisci^56^ | 2015 | Sx | CEA | Low | Low | Low | Low | High | Low | Low | Low | NA | NA |  |
| Chisci^64^ | 2022 | Sx | CEA | Low | Low | Low | Low | Low | Low | High | Low | NA | NA |  |
| Cho^55^ | 2022 | Sx | CEA | Low | Low | High | High | High | High | High | Low | NA | NA |  |
| Cho^55^ | 2022 | Sx | CEA | Low | Low | High | High | High | High | High | Low | NA | NA |  |
| Cho^55^ | 2022 | ASx | CEA | Low | Low | High | High | High | High | High | Low | NA | NA |  |
| Cho^55^ | 2022 | ASx | CEA | Low | Low | High | High | High | High | High | Low | NA | NA |  |
| Cho^55^ | 2022 | Sx | CAS | Low | Low | High | High | High | High | High | Low | NA | NA |  |
| Cho^55^ | 2022 | Sx | CAS | Low | Low | High | High | High | High | High | Low | NA | NA |  |
| Cho^55^ | 2022 | ASx | CAS | Low | Low | High | High | High | High | High | Low | NA | NA |  |
| Cho^55^ | 2022 | ASx | CAS | Low | Low | High | High | High | High | High | Low | NA | NA |  |
| Chung^53^ | 2020 | Sx | CEA | Low | Low | High | High | High | Low | Low | Low | NA | NA |  |
| Claus^52^ | 2010 | Sx | CAS | Low | Low | Low | Low | Low | Low | Low | Low | NA | NA |  |
| Claus^52^ | 2010 | ASx | CAS | Low | Low | Low | Low | Low | Low | Low | Low | NA | NA |  |
| Cohen^51^ | 2015 | Sx | CAS | Low | Low | High | High | High | Low | High | Low | High | High |  |
| Cohen^51^ | 2015 | ASx | CAS | Low | Low | High | High | High | Low | High | Low | High | High |  |
| Coppi^50^ | | 2005 | Sx | CEA | Low | Low | High | High | High | High | High | Low | High | Unclear |
| Coppi^50^ | | 2005 | ASx | CEA | Low | Low | Low | High | High | High | High | Low | High | Unclear |
| Dellagrammaticas^47^ | 2007 | Sx | CEA | Low | Low | High | Low | High | Low | Low | Low | NA | NA |  |
| Dellagrammaticas^47^ | 2007 | ASx | CEA | Low | Low | High | Low | High | Low | Low | Low | NA | NA |  |
| De Donato^48^ | 2008 | Sx | CAS | Int | Low | High | High | High | High | Low | NA | High | Low |  |
| De Donato^48^ | 2008 | ASx | CAS | Int | Low | High | High | High | High | Low | NA | High | Low |  |
| De Blasis^49^ | 2023 | Sx | CEA | Low | Low | Low | Low | Low | Low | High | Low | NA | NA |  |
| De Rango^65^ | 2011 | Sx | CEA | Low | Low | High | Low | High | Low | Low | Low | NA | NA |  |
| De Rango^65^ | 2011 | ASx | CEA | Low | Low | High | Low | High | Low | Low | Low | NA | NA |  |
| De Rango^65^ | 2011 | Sx | CAS | Low | Low | High | Low | High | Low | Low | Low | NA | NA |  |
| De Rango^65^ | 2011 | ASx | CAS | Low | Low | High | Low | High | Low | Low | Low | NA | NA |  |
| De Rango^66^ | 2016 | Sx | CEA | Low | Low | High | Low | High | Low | Low | Low | High | High |  |
| De Rango^66^ | 2016 | ASx | CEA | Low | Low | Low | Low | High | Low | Low | Low | High | High |  |
| De Rango^66^ | 2016 | Sx | CAS | Low | Low | High | Low | High | Low | Low | Low | High | High |  |
| De Rango^66^ | 2016 | ASx | CAS | Low | Low | Low | Low | High | Low | Low | Low | High | High |  |
| Derdeyn^67^ | 2014 | Sx | CAS | Low | Low | Low | Low | Low | Low | Low | Low | High | Unclear |  |
| Dorigo^68^ | 2007 | Sx | CEA | Low | Low | High | Low | Int | High | Low | Low | High | High |  |
| Dumont^69^ | 2013 | Sx | CAS | Low | Low | High | Low | Low | Low | Int | Low | NA | NA |  |
| Dumont^69^ | 2013 | ASx | CAS | Low | Low | Low | Low | Low | Low | Int | Low | NA | NA |  |
| Duschek^70^ | 2011 | ASx | CEA | Low | Low | Low | Low | Low | High | High | Low | High | Unclear |  |
| Eckstein^71^ | 2008 | Sx | CEA | Low | Low | Low | Low | Low | Low | Low | Low | High | High |  |
| Eckstein^71^ | 2008 | Sx | CAS | Low | Low | Low | Low | Low | Low | Low | Low | High | High |  |
| ECSTCG^74^ | 1998 | Sx | CEA | Low | Low | Low | Low | Low | Low | Low | Low | NA | NA |  |
| Ederle^72^ | 2010 | Sx | CEA | Low | Low | Low | Low | Low | Low | Low | Low | High | High |  |
| Ederle^72^ | 2010 | Sx | CAS | Low | Low | Low | Low | Low | Low | Low | Low | High | High |  |
| Elderly^73^ | 2022 | Sx | CEA | Low | Unclear | Low | High | Low | Low | High | Low | NA | NA |  |
| Elshikhawoda^285^ | 2024 | Sx | CEA | Low | Unclear | Low | High | Low | High | High | Low | NA | NA |  |
| Fanous^75^ | 2015 | Sx | CAS | Low | Low | High | High | Low | Low | High | Low | NA | NA |  |
| Fearn^76^ | 1998 | Sx | CEA | Low | High | Low | Low | Low | High | High | Int | High | Low |  |
| Feasby^77^ | 2007 | Sx | CEA | Low | Low | High | High | Low | High | High | Int | NA | NA |  |
| Feasby^77^ | 2007 | ASx | CEA | Low | Low | High | High | Low | High | High | Int | NA | NA |  |
| Ferrero^78^ | 2010 | Sx | CEA | Low | Low | Low | High | Low | Low | Low | Low | NA | NA |  |
| Ferrero^79^ | 2014 | Sx | CEA | Low | Low | Low | High | Low | Low | Low | Low | High | High |  |
| Finocchi^80^ | 1997 | Sx | CEA | Low | Low | High | High | High | Low | High | Low | NA | NA |  |
| Finocchi^80^ | 1997 | ASx | CEA | Low | Low | High | High | High | Low | High | Low | NA | NA |  |
| Fornelli^81^ | 2021 | ASx | CAS | Low | Low | Low | High | Low | Low | High | Int | High | Unclear |  |
| Fortin^82^ | 2020 | Sx | CEA | Low | Low | Low | High | Low | High | High | Low | NA | NA |  |
| Gabrielli^289^ | 2023 | ASx | CEA | Low | Low | Low | Low | Low | High | High | Low | NA | NA |  |
| Gabrielli^289^ | 2023 | ASx | CAS | Low | Low | Low | Low | Low | High | High | Low | NA | NA |  |
| Garcia-Rodriguez^83^ | 2012 | Sx | CAS | High | Low | High | High | High | High | Low | Low | NA | NA |  |
| Garcia-Rodriguez^83^ | 2012 | ASx | CAS | High | Low | High | High | High | High | Low | Low | NA | NA |  |
| Geisbüsch^84^ | 2012 | Sx | CAS | Low | Low | High | Low | High | Low | Low | Low | NA | NA |  |
| Geisbüsch^84^ | 2012 | ASx | CAS | Low | Low | High | Low | High | Low | Low | Low | NA | NA |  |
| Gensicke^85^ | 2015 | Sx | CEA | Low | Low | Low | Low | Low | Low | Low | NA | High | Low |  |
| Gensicke^85^ | 2015 | Sx | CAS | Low | Low | Low | Low | Low | Low | Low | NA | High | Low |  |
| Golledge^86^ | 1996 | Sx | CEA | Low | Low | Low | Low | Int | Low | High | Low | High | Unclear |  |
| Goode^87^ | 2013 | Sx | CEA | Low | Unclear | Low | Low | High | High | High | Low | NA | NA |  |
| Goode^87^ | 2013 | ASx | CEA | Low | Unclear | Low | Low | High | High | High | Low | NA | NA |  |
| Grant^88^ | 2010 | Sx | CAS | High | Low | High | High | High | Low | Low | Low | NA | NA |  |
| Grant^88^ | 2010 | ASx | CAS | High | Low | High | High | High | Low | Low | Low | NA | NA |  |
| Gray^89^ | 2009 | Sx | CAS | High | Low | High | Low | High | Low | Low | Low | NA | NA |  |
| Gray^89^ | 2009 | ASx | CAS | High | Low | High | Low | High | Low | Low | Low | NA | NA |  |
| Gray^90^ | 2006 | Sx | CAS | High | Low | High | Low | High | Low | Low | Low | NA | NA |  |
| Gray^90^ | 2006 | ASx | CAS | High | Low | High | Low | High | Low | Low | Low | NA | NA |  |
| Gray^91^ | 2007 | Sx | CAS | Low | Low | High | Low | Low | High | Low | Low | NA | NA |  |
| Gray^91^ | 2007 | ASx | CAS | Low | Low | High | Low | Low | High | Low | Low | NA | NA |  |
| Grimm^92^ | 2014 | Sx | CEA | Low | Low | High | High | High | High | Low | Low | NA | NA |  |
| Grimm^92^ | 2014 | ASx | CEA | Low | Low | High | High | High | High | Low | Low | NA | NA |  |
| Grimm^92^ | 2014 | Sx | CAS | Low | Low | High | High | High | High | Low | Low | NA | NA |  |
| Grimm^92^ | 2014 | ASx | CAS | Low | Low | High | High | High | High | Low | Low | NA | NA |  |
| Gröschel^93^ | 2008 | Sx | CAS | Low | Unclear | Low | Low | Low | Low | Low | Low | NA | NA |  |
| Hajiyev^279^ | 2024 | Sx | CAS | Low | Unclear | Low | Unclear | Low | Low | Int | Int | High | Unclear |  |
| Hajiyev^279^ | 2024 | ASx | CAS | Low | Unclear | Low | Unclear | Low | Low | Int | Int | High | Unclear |  |
| Halliday^94^ | 2021 | ASx | CEA | Low | Unclear | Low | Low | Low | Low | Low | Low | NA | NA |  |
| Halliday^94^ | 2021 | ASx | CAS | Low | Unclear | Low | Low | Low | Low | Low | Low | NA | NA |  |
| Halliday^95^ | 2010 | ASx | CEA | Low | Unclear | High | Low | High | High | Low | Low | High | Unclear |  |
| Halm^96^ | 2003 | Sx | CEA | Low | Low | High | High | High | High | Low | Low | NA | NA |  |
| Halm^96^ | 2003 | ASx | CEA | Low | Low | High | High | High | High | Low | Low | NA | NA |  |
| Halm^97^ | 2009 | Sx | CEA | Low | Low | High | High | High | High | Low | Low | NA | NA |  |
| Hartmann^98^ | 1999 | Sx | CEA | Low | Low | High | Low | High | Low | High | Low | NA | NA |  |
| Hartmann^98^ | 1999 | ASx | CEA | Low | Low | High | Low | High | Low | High | Low | NA | NA |  |
| Hause^99^ | 2022 | Sx | CEA | Low | Low | Low | Low | Low | High | Low | Low | NA | NA |  |
| Hernandez-Fernandez^100^ | 2014 | Sx | CAS | Low | Low | High | High | High | Low | High | Low | NA | NA |  |
| Hernandez-Fernandez^100^ | 2014 | ASx | CAS | Low | Low | High | High | High | Low | High | Low | NA | NA |  |
| Hicks^101^ | 2018 | ASx | CEA | Low | Unclear | Low | Low | Int | Low | High | Low | NA | NA |  |
| Hobson^102^ | 2003 | Sx | CAS | Low | Low | High | Low | High | Low | High | Low | NA | NA |  |
| Hobson^102^ | 2003 | ASx | CAS | Low | Low | High | Low | High | Low | High | Low | NA | NA |  |
| Hobson^103^ | 1993 | ASx | CEA | Low | Low | Low | Low | High | High | High | Low | NA | NA |  |
| Hoffmann^104^ | 1999 | Sx | CEA | Low | Low | Low | Low | Int | High | Low | Low | NA | NA |  |
| Hong^105^ | 2015 | ASx | CAS | Low | Low | Low | High | Low | High | Int | Low | NA | NA |  |
| Hong^106^ | 2017 | Sx | CAS | Low | Low | High | High | Low | High | Int | Low | NA | NA |  |
| Ielasi^107^ | 2010 | Sx | CAS | Low | Low | High | High | High | Low | Low | Low | High | Low |  |
| Ielasi^107^ | 2010 | ASx | CAS | Low | Low | High | High | High | Low | Low | Low | High | Low |  |
| Illig^108^ | 2003 | Sx | CEA | High | Low | High | High | High | Low | High | Low | NA | NA |  |
| Illig^108^ | 2003 | ASx | CEA | High | Low | High | High | High | Low | High | Low | NA | NA |  |
| Ishiguro^109^ | 2015 | ASx | CEA | Low | Unclear | Low | High | Int | High | High | Low | High | Low |  |
| Iwasaki^110^ | 2011 | Sx | CEA | Low | Unclear | Low | Low | Int | High | High | Low | High | Low |  |
| Iwasaki^110^ | 2011 | ASx | CEA | Low | Unclear | Low | Low | Int | High | High | Low | High | Low |  |
| Jalbert^112^ | 2015 | Sx | CAS | Low | Low | High | High | High | Low | High | Low | NA | NA |  |
| Jalbert^112^ | 2015 | ASx | CAS | Low | Low | High | High | High | Low | High | Low | NA | NA |  |
| Jang^113^ | 2021 | ASx | CAS | Low | Low | Low | High | Low | Low | High | Low | NA | NA |  |
| Johansson^114^ | 2008 | Sx | CEA | Low | Low | Low | High | Low | High | High | Low | NA | NA |  |
| Jonsson^118^ | 2015 | Sx | CAS | Low | Low | Low | High | Low | Low | High | Low | NA | NA |  |
| Jonsson^115^ | 2023 | Sx | CEA | Low | Low | Low | High | Low | Low | High | Low | NA | NA |  |
| Jordan^116^ | 1999 | Sx | CAS | Low | Low | High | High | High | Low | High | Low | NA | NA |  |
| Jordan^116^ | 1999 | ASx | CAS | Low | Low | High | High | High | Low | High | Low | NA | NA |  |
| Jordan^116^ | 1999 | Sx | CEA | Low | Low | High | High | High | Low | High | Low | NA | NA |  |
| Jordan^116^ | 1999 | ASx | CEA | Low | Low | High | High | High | Low | High | Low | NA | NA |  |
| Joviliano^117^ | 2022 | Sx | CAS | Low | Low | Low | Low | High | Low | Low | Low | NA | NA |  |
| Joviliano^117^ | 2022 | Sx | CEA | Low | Low | Low | Low | High | Low | Low | Low | NA | NA |  |
| Kallmayer^282^ | 2024 | Sx | CEA | Low | Low | High | Low | Int | High | High | Int | NA | NA |  |
| Kallmayer^282^ | 2024 | ASx | CEA | Low | Low | High | Low | Int | High | High | Int | NA | NA |  |
| Kallmayer^282^ | 2024 | Sx | CAS | Low | Low | High | Low | Int | High | High | Int | NA | NA |  |
| Kallmayer^282^ | 2024 | ASx | CAS | Low | Low | High | Low | Int | High | High | Int | NA | NA |  |
| Kang^119^ | 2021 | ASx | CEA | Low | Low | Low | High | High | Low | High | Low | High | Unclear |  |
| Kang^119^ | 2021 | ASx | CAS | Low | Low | Low | High | High | Low | High | Low | High | Unclear |  |
| Kang^281^ | 2023 | Sx | CAS | Low | Unclear | Low | High | Int | High | High | Low | NA | NA |  |
| Kang^281^ | 2023 | ASx | CAS | Low | Unclear | Low | High | Int | High | High | Low | NA | NA |  |
| Karkos^120^ | 2010 | Sx | CAS | Low | Low | High | High | High | High | Low | Low | NA | NA |  |
| Karkos^120^ | 2010 | ASx | CAS | Low | Low | High | High | High | High | Low | Low | NA | NA |  |
| Karthaus^121^ | 2018 | Sx | CEA | Low | Low | Low | High | High | Low | High | Low | NA | NA |  |
| Kashyap^122^ | 2019 | Sx | CEA | Low | Low | High | High | High | High | Int | Low | NA | NA |  |
| Kashyap^122^ | 2019 | ASx | CEA | Low | Low | High | High | High | High | Int | Low | NA | NA |  |
| Kastrup^123^ | 2005 | Sx | CAS | Low | Low | Low | Low | High | Low | Low | Low | NA | NA |  |
| Kastrup^123^ | 2005 | ASx | CAS | Low | Low | Low | Low | High | Low | Low | Low | NA | NA |  |
| Kastrup^124^ | 2003 | Sx | CEA | Low | Low | High | High | High | Low | Low | Low | NA | NA |  |
| Kastrup^124^ | 2003 | ASx | CEA | Low | Low | High | High | High | Low | Low | Low | NA | NA |  |
| Katzen^125^ | 2007 | Sx | CAS | High | High | High | Low | High | Low | Low | Low | NA | NA |  |
| Katzen^125^ | 2007 | ASx | CAS | High | High | High | Low | High | Low | Low | Low | NA | NA |  |
| Kawaguchi^126^ | 1994 | Sx | CEA | High | High | High | High | Int | High | High | High | High | Low |  |
| Kazandjian^127^ | 2017 | Sx | CEA | Low | High | Low | Low | High | Low | High | Low | NA | NA |  |
| Keyhani^128^ | 2019 | ASx | CEA | Low | Low | Low | High | High | Low | Low | Low | High | High |  |
| Kharroubi^129^ | 2020 | Sx | CEA | High | Low | High | High | High | High | High | Low | NA | NA |  |
| Kharroubi^129^ | 2020 | ASx | CEA | High | Low | High | High | High | High | High | Low | NA | NA |  |
| Kibrik^130^ | 2022 | Sx | CEA | High | Low | High | High | Int | Low | High | Low | NA | NA |  |
| Kibrik^130^ | 2022 | ASx | CEA | High | Low | High | High | Int | Low | High | Low | NA | NA |  |
| Kim^131^ | 2022 | Sx | CEA | Low | Low | High | High | High | High | Low | Low | NA | NA |  |
| Kim^131^ | 2022 | ASx | CEA | Low | Low | High | High | High | High | Low | Low | NA | NA |  |
| Kim^133^ | 2022 | Sx | CEA | Low | Low | Low | High | High | High | High | Low | NA | NA |  |
| Kimiagar^132^ | 2012 | Sx | CEA | High | Low | Low | Low | High | Low | Low | Low | NA | NA |  |
| King^134^ | 2021 | Sx | CEA | High | Low | Low | High | High | Low | Low | Low | NA | NA |  |
| King^134^ | 2021 | ASx | CEA | High | Low | Low | High | High | Low | Low | Low | NA | NA |  |
| Kirchhoff^135^ | 2023 | Sx | CEA | Low | Low | High | Low | High | Low | Low | Low | NA | NA |  |
| Kirchhoff^135^ | 2023 | ASx | CEA | Low | Low | High | Low | High | Low | Low | Low | NA | NA |  |
| Kjørstad^136^ | 2017 | Sx | CEA | Low | Low | Low | Low | Int | Low | Low | Low | NA | NA |  |
| Knappich^137^ | 2017 | ASx | CAS | Low | Low | Low | High | Low | Low | High | Low | NA | NA |  |
| Knappich^137^ | 2017 | Sx | CAS | Low | Low | Low | High | Low | Low | High | Low | NA | NA |  |
| Kök^283^ | 2024 | Sx | CEA | Low | Low | High | High | Low | Low | High | Low | NA | NA |  |
| Kök^283^ | 2024 | Sx | CAS | Low | Low | High | High | Low | Low | High | Low | NA | NA |  |
| Kök^283^ | 2024 | ASx | CAS | Low | Low | High | High | Low | Low | High | Low | NA | NA |  |
| Köklü^138^ | 2021 | Sx | CAS | Low | Low | Low | High | High | High | High | Low | NA | NA |  |
| Köklü^138^ | 2021 | ASx | CAS | Low | Low | Low | High | High | High | High | Low | NA | NA |  |
| Kouvelos^139^ | 2017 | Sx | CAS | High | Low | Low | High | High | Low | Low | Low | High | Low |  |
| Kouvelos^139^ | 2017 | ASx | CAS | High | Low | Low | High | High | Low | Low | Low | High | Low |  |
| Kretz^140^ | 2015 | Sx | CEA | Low | Low | Low | Low | High | Low | High | Low | NA | NA |  |
| Kucey^141^ | 1998 | ASx | CEA | Low | Low | High | High | High | Low | High | Low | NA | NA |  |
| Kucey^141^ | 1998 | Sx | CEA | Low | Low | High | High | High | Low | High | Low | NA | NA |  |
| Lago^142^ | 2013 | Sx | CAS | Low | Low | Low | Low | High | High | Low | Low | High | High |  |
| Lahlouh^143^ | 2024 | Sx | CAS | High | Unclear | Low | High | Low | Low | High | Low | NA | NA |  |
| Lane^144^ | 2003 | Sx | CEA | Low | Low | Low | Low | High | Low | High | High | High | Low |  |
| Lane^144^ | 2003 | ASx | CEA | Low | Low | Low | Low | High | Low | High | High | High | Low |  |
| Langhoff^145^ | 2014 | Sx | CAS | Low | Unclear | High | Low | High | High | High | Low | High | High |  |
| Langhoff^145^ | 2014 | ASx | CAS | Low | Unclear | High | Low | High | High | High | Low | High | High |  |
| Latacz^146^ | 2021 | Sx | CAS | Low | Low | Low | High | High | Low | High | Low | High | High |  |
| Latz^147^ | 2021 | ASx | CAS | Low | Low | Low | Low | Low | High | High | Low | NA | NA |  |
| Lawaetz^148^ | 2021 | Sx | CEA | Low | Low | Low | Low | Low | Low | Low | Low | Low | Low |  |
| Lee^149^ | 2019 | Sx | CAS | Low | Low | Low | Low | Int | Low | Low | Low | High | Unclear |  |
| Lee^149^ | 2019 | Sx | CEA | Low | Low | Low | High | Int | Low | Low | Low | High | Unclear |  |
| Lepore^150^ | 2001 | Sx | CEA | Low | Low | High | High | Low | Low | High | Low | NA | NA |  |
| Lepore^150^ | 2001 | ASx | CEA | Low | Low | High | High | Low | Low | High | Low | NA | NA |  |
| Leško^151^ | 2021 | Sx | CEA | Low | Low | High | High | High | Low | Low | NA | High | Unclear |  |
| Leško^151^ | 2021 | ASx | CEA | Low | Low | High | High | High | Low | Low | NA | High | Unclear |  |
| Leško^151^ | 2021 | Sx | CAS | Low | Low | High | High | High | Low | Low | NA | High | Unclear |  |
| Leško^151^ | 2021 | ASx | CAS | Low | Low | High | High | High | Low | Low | NA | High | Unclear |  |
| Levy^152^ | 2023 | Sx | CAS | Low | Unclear | Low | Low | High | Low | High | Low | NA | NA |  |
| Levy^152^ | 2023 | ASx | CAS | Low | Unclear | Low | Low | High | Low | High | Low | NA | NA |  |
| Levy^152^ | 2023 | Sx | CEA | Low | Unclear | Low | Low | High | Low | High | Low | NA | NA |  |
| Levy^152^ | 2023 | ASx | CEA | Low | Unclear | Low | Low | High | Low | High | Low | NA | NA |  |
| Libman^153^ | 1994 | ASx | CEA | Low | Low | Low | Low | High | High | Low | Low | NA | NA |  |
| Lim^154^ | 2019 | ASx | CEA | Low | Low | Low | High | Int | Low | High | Low | NA | NA |  |
| Lim^154^ | 2019 | ASx | CAS | Low | Low | Low | High | Int | Low | High | Low | NA | NA |  |
| Lindström^155^ | 2012 | ASx | CAS | Low | Low | High | High | High | Low | High | Low | NA | NA |  |
| Lindström^155^ | 2012 | Sx | CAS | Low | Low | High | High | High | Low | High | Low | NA | NA |  |
| Lindström^155^ | 2012 | ASx | CEA | Low | Low | High | High | High | Low | High | Low | NA | NA |  |
| Lindström^155^ | 2012 | Sx | CEA | Low | Low | High | High | High | Low | High | Low | NA | NA |  |
| Lindström^155^ | 2012 | ASx | CEA | Low | Low | High | High | High | Low | High | Low | NA | NA |  |
| Liu^156^ | 2016 | Sx | CAS | High | Low | Low | High | Low | Low | High | Low | High | High |  |
| Liu^157^ | 2023 | Sx | CAS | Low | Unclear | Low | High | Int | Low | Low | Low | NA | NA |  |
| Liu^157^ | 2023 | ASx | CAS | Low | Unclear | Low | High | Int | Low | Low | Low | NA | NA |  |
| Loftus^158^ | 2016 | Sx | CEA | Low | Low | Low | High | Low | High | High | Low | NA | NA |  |
| Longo^159^ | 2005 | Sx | CAS | Low | Unclear | High | Low | High | Low | High | Low | NA | NA |  |
| Longo^159^ | 2005 | ASx | CAS | Low | Unclear | High | Low | High | Low | High | Low | NA | NA |  |
| Lübke^160^ | 2015 | Sx | CEA | Low | Low | High | Low | High | High | Low | Low | NA | NA |  |
| Lübke^160^ | 2015 | ASx | CEA | Low | Low | High | Low | High | High | Low | Low | NA | NA |  |
| Lutz^161^ | 2008 | Sx | CEA | Low | Low | High | High | High | Low | High | Low | NA | NA |  |
| Lutz^161^ | 2008 | ASx | CEA | Low | Low | High | High | High | Low | High | Low | NA | NA |  |
| Madden^162^ | 2022 | Sx | CEA | Low | Low | High | Low | High | High | High | Low | NA | NA |  |
| Madden^162^ | 2022 | ASx | CEA | Low | Low | High | Low | High | High | High | Low | NA | NA |  |
| Madhani^163^ | 2022 | Sx | CAS | High | High | High | High | Int | High | High | Low | NA | NA |  |
| Madhani^163^ | 2022 | Sx | CEA | High | High | High | High | Int | High | High | Low | NA | NA |  |
| Mallela^164^ | 2022 | ASx | CEA | Low | Low | Low | High | Int | Low | High | Low | NA | NA |  |
| Mallick^165^ | 2020 | ASx | CAS | Low | High | Low | High | Low | High | High | Low | NA | NA |  |
| Mannheim^166^ | 2017 | ASx | CEA | Low | Low | Low | Low | High | High | Low | Low | High | Unclear |  |
| Mannheim^166^ | 2017 | ASx | CAS | Low | Low | Low | Low | High | High | Low | Low | High | Unclear |  |
| Mansour^167^ | 2011 | Sx | CAS | High | Low | High | Low | High | Low | Low | Low | NA | NA |  |
| Mansour^167^ | 2011 | ASx | CAS | High | Low | High | Low | High | Low | Low | Low | NA | NA |  |
| Macharzina^168^ | 2020 | Sx | CEA | Low | Low | High | Low | High | Low | Low | Low | NA | NA |  |
| Macharzina^168^ | 2020 | ASx | CEA | Low | Low | High | Low | High | Low | Low | Low | NA | NA |  |
| Marine^169^ | 2006 | ASx | CEA | Low | Low | Low | High | High | Low | Low | Low | NA | NA |  |
| Martín-Morales^170^ | 2019 | ASx | CEA | Low | Low | High | High | High | High | High | Low | NA | NA |  |
| Mas^171^ | 2014 | Sx | CEA | Low | Low | Low | Low | Low | Low | Low | Low | Low | Low |  |
| Mas^171^ | 2014 | Sx | CAS | Low | Low | Low | Low | Low | Low | Low | Low | Low | Low |  |
| Mastrorilli^172^ | 2023 | ASx | CAS | Low | Low | Low | High | Low | High | High | Low | Low | Low |  |
| Mastrorilli^173^ | 2022 | Sx | CEA | Low | Low | Low | Low | Low | Low | Low | Int | Low | Low |  |
| Mazzaccaro^174^ | 2012 | Sx | CAS | High | Unclear | High | High | High | Low | High | Low | NA | NA |  |
| Mazzaccaro^174^ | 2012 | ASx | CAS | High | Unclear | High | High | High | Low | High | Low | NA | NA |  |
| McCrory^175^ | 1993 | Sx | CEA | Low | High | High | High | High | Low | High | Int | NA | NA |  |
| McCrory^175^ | 1993 | ASx | CEA | Low | High | High | High | High | Low | High | Int | NA | NA |  |
| McKevitt^176^ | 2004 | Sx | CAS | High | Low | High | Low | Low | Low | Low | Low | NA | NA |  |
| Mehta^177^ | 2009 | Sx | CAS | Low | Low | High | High | High | Low | High | Int | NA | NA |  |
| Mehta^177^ | 2009 | ASx | CAS | Low | Low | High | High | High | Low | High | Int | NA | NA |  |
| Meller^178^ | 2016 | Sx | CEA | Low | Low | High | High | High | Low | High | Low | NA | NA |  |
| Meller^178^ | 2016 | ASx | CEA | Low | Low | High | High | High | Low | High | Low | NA | NA |  |
| Meller^178^ | 2016 | Sx | CAS | Low | Low | High | High | High | Low | High | Low | NA | NA |  |
| Meller^178^ | 2016 | ASx | CAS | Low | Low | High | High | High | Low | High | Low | NA | NA |  |
| Merlini^179^ | 2014 | Sx | CEA | Low | Low | Low | Low | Low | Low | High | Low | NA | NA |  |
| Meyer^180^ | 1991 | Sx | CEA | Int | Low | High | High | High | Low | Low | Low | NA | NA |  |
| Micari^181^ | 2010 | Sx | CAS | Int | Unclear | High | Low | High | Low | Low | Low | NA | NA |  |
| Micari^181^ | 2010 | Sx | CAS | Int | Unclear | High | Low | High | Low | Low | Low | NA | NA |  |
| Micari^181^ | 2010 | ASx | CAS | Int | Unclear | High | Low | High | Low | Low | Low | NA | NA |  |
| Micari^181^ | 2010 | ASx | CAS | Int | Unclear | High | Low | High | Low | Low | Low | NA | NA |  |
| Micheel^182^ | 2022 | Sx | CEA | Low | Low | Low | High | Low | Low | High | Low | NA | NA |  |
| Middleton^183^ | 2002 | Sx | CEA | Low | Low | Low | High | Low | High | High | Low | NA | NA |  |
| Middleton^183^ | 2002 | ASx | CEA | Low | Low | Low | High | Low | High | High | Low | NA | NA |  |
| Miyachi^184^ | 2012 | Sx | CAS | High | Low | High | High | High | Low | High | Low | NA | NA |  |
| Miyachi^184^ | 2012 | ASx | CAS | High | Low | High | High | High | Low | High | Low | NA | NA |  |
| Mo^185^ | 2016 | Sx | CAS | Low | Low | Low | Low | High | High | High | High | High | High |  |
| Mo^185^ | 2016 | ASx | CAS | Low | Low | Low | Low | Low | High | High | High | High | High |  |
| Montorsi^186^ | 2010 | Sx | CAS | Low | Low | High | Low | High | Low | Low | Low | NA | NA |  |
| Montorsi^186^ | 2010 | Sx | CAS | Low | Low | High | Low | High | Low | Low | Low | NA | NA |  |
| Montorsi^186^ | 2010 | ASx | CAS | Low | Low | High | Low | High | Low | Low | Low | NA | NA |  |
| Montorsi^186^ | 2010 | ASx | CAS | Low | Low | High | Low | High | Low | Low | Low | NA | NA |  |
| Morales-Gisbert^187^ | 2017 | Sx | CEA | Low | Low | High | High | High | High | High | Low | High | High |  |
| Morales-Gisbert^187^ | 2017 | ASx | CEA | Low | Low | High | High | High | High | High | Low | High | High |  |
| Murtidjaja^188^ | 2021 | Sx | CEA | Low | Low | High | High | High | High | High | Int | NA | NA |  |
| Murtidjaja^188^ | 2021 | ASx | CEA | Low | Low | High | High | High | High | High | Int | NA | NA |  |
| Murtidjaja^188^ | 2021 | Sx | CAS | Low | Low | High | High | High | High | High | Int | NA | NA |  |
| Murtidjaja^188^ | 2021 | ASx | CAS | Low | Low | High | High | High | High | High | Int | NA | NA |  |
| Musialek^189^ | 2016 | Sx | CAS | Low | Low | High | Low | High | Low | Low | Low | NA | NA |  |
| Musialek^189^ | 2016 | ASx | CAS | Low | Low | High | Low | High | Low | Low | Low | NA | NA |  |
| Mutirangura^190^ | 2016 | Sx | CEA | Low | Low | Low | Low | Low | High | High | Low | High | High |  |
| Nakagawa^191^ | 2020 | Sx | CAS | Low | Low | Low | High | Low | Low | Int | Low | NA | NA |  |
| Nakagawa^191^ | 2020 | ASx | CAS | Low | Low | Low | High | Low | Low | Int | Low | NA | NA |  |
| Ngo^192^ | 2020 | Sx | CEA | Low | Low | Low | High | Low | Low | High | Low | Low | Low |  |
| Ngo^192^ | 2020 | Sx | CEA | Low | Low | Low | High | Low | Low | High | Low | Low | Low |  |
| Nikas^193^ | 2012 | Sx | CAS | Low | Low | High | Low | High | Low | Low | Low | NA | NA |  |
| Nikas^193^ | 2012 | ASx | CAS | Low | Low | High | Low | High | Low | Low | Low | NA | NA |  |
| Nishimoto^290^ | 2024 | Sx | CAS | Low | Low | Low | High | Int | Low | High | Low | NA | NA |  |
| Nordanstig^194^ | 2017 | Sx | CEA | Low | Low | Low | Low | Low | High | Low | Low | NA | NA |  |
| Nowakowski^195^ | 2021 | Sx | CAS | Low | Unclear | High | Low | High | High | High | Low | NA | NA |  |
| Nowakowski^195^ | 2021 | ASx | CAS | Low | Unclear | High | Low | High | High | High | Low | NA | NA |  |
| Ogata^196^ | 2014 | Sx | CAS | Low | Low | High | Low | High | Low | High | Low | High | Low |  |
| Ogata^196^ | 2014 | ASx | CAS | Low | Low | High | Low | High | Low | High | Low | High | Low |  |
| Omura^291^ | 2024 | Sx | CAS | Low | Low | High | High | Int | Low | High | Low | NA | NA |  |
| Omura^291^ | 2024 | ASx | CAS | Low | Low | High | High | Int | Low | High | Low | NA | NA |  |
| Ouriel^197^ | 2001 | Sx | CEA | Low | Low | High | Low | High | High | High | Int | NA | NA |  |
| Ouriel^197^ | 2001 | ASx | CEA | Low | Low | High | Low | High | High | High | Int | NA | NA |  |
| Papakostas^198^ | 2014 | Sx | CEA | Low | Low | High | High | Int | High | Low | Low | NA | NA |  |
| Papakostas^198^ | 2014 | ASx | CEA | Low | Low | High | High | Int | High | Low | Low | NA | NA |  |
| Pascot^199^ | 2018 | ASx | CEA | Low | Unclear | Low | High | Low | High | High | Low | NA | NA |  |
| Paukovits^200^ | 2008 | Sx | CEA | Low | Low | High | High | Int | Low | Low | Low | NA | NA |  |
| Paukovits^200^ | 2008 | ASx | CEA | Low | Low | High | High | Int | Low | Low | Low | NA | NA |  |
| Perini^201^ | 2017 | Sx | CEA | Low | Low | Low | Low | Int | High | Low | Low | NA | NA |  |
| Perona^202^ | 2009 | Sx | CAS | Low | Low | Low | High | Int | Low | High | Low | NA | NA |  |
| Perona^202^ | 2009 | ASx | CAS | Low | Low | Low | High | Int | Low | High | Low | NA | NA |  |
| Petkoska^203^ | 2023 | Sx | CAS | Low | Low | High | Low | Int | Low | High | Low | High | Unclear |  |
| Petkoska^203^ | 2023 | ASx | CAS | Low | Low | High | Low | Int | Low | High | Low | High | Unclear |  |
| Piazza^204^ | 2018 | ASx | CEA | Low | Low | Low | High | Low | Low | Low | Low | NA | NA |  |
| Pieniazek^205^ | 2012 | Sx | CAS | Low | Unclear | Low | Unclear | Int | Low | Low | Low | NA | NA |  |
| Pieniazek^205^ | 2012 | ASx | CAS | Low | Unclear | Low | Unclear | Int | Low | Low | Low | NA | NA |  |
| Pini^206^ | 2020 | ASx | CEA | Low | Unclear | Low | Low | Int | High | Low | Low | NA | NA |  |
| Pini^207^ | 2017 | Sx | CAS | Low | Low | High | Low | Int | Low | Low | Low | NA | NA |  |
| Pini^208^ | 2012 | ASx | CAS | Low | Low | High | Low | Int | Low | Low | Low | NA | NA |  |
| Poisson^209^ | 2010 | Sx | CEA | Low | Unclear | Low | High | Int | High | High | Low | Low | Low |  |
| Poorthuis^210^ | 2022 | Sx | CEA | Low | Unclear | High | Low | Int | High | High | Low | NA | NA |  |
| Poulias^211^ | 1994 | Sx | CEA | Low | Low | High | Unclear | Int | High | High | High | NA | NA |  |
| Poulias^211^ | 1994 | ASx | CEA | Low | Low | High | Unclear | Int | High | High | High | NA | NA |  |
| Quispe Orozco^212^ | 2021 | Sx | CAS | Low | Unclear | Low | Low | Int | Low | High | NA | Low | NA |  |
| Qumsiyeh^213^ | 2023 | Sx | CEA | Low | Low | High | High | Int | High | High | Low | NA | NA |  |
| Qumsiyeh^213^ | 2023 | ASx | CEA | Low | Low | High | High | Int | High | High | Low | NA | NA |  |
| Radu^214^ | 2013 | Sx | CAS | Low | Low | Low | Low | Int | Low | High | Low | High | Unclear |  |
| Radu^214^ | 2013 | ASx | CAS | Low | Low | Low | Low | Int | Low | High | Low | High | Unclear |  |
| Randall^215^ | 2010 | Sx | CEA | Low | Low | Low | Low | Int | Low | Low | Low | High | Unclear |  |
| Rantner^216^ | 2006 | Sx | CEA | Low | Unclear | Low | Int | Low | Low | High | Low | NA | NA |  |
| Rantner^217^ | 2015 | Sx | CEA | Low | Low | Low | High | Low | Low | Low | Low | NA | NA |  |
| Rasiova^218^ | 2017 | Sx | CAS | Low | Low | Low | Low | Int | Low | Low | Low | NA | NA |  |
| Rasiova^218^ | 2017 | ASx | CAS | Low | Low | Low | Low | Int | Low | Low | Low | NA | NA |  |
| Rathenborg^219^ | 2013 | Sx | CEA | Low | Low | Low | High | Low | Low | High | Low | NA | NA |  |
| Ratner^220^ | 2023 | Sx | CEA | Low | Unclear | High | High | Int | High | High | Low | NA | NA |  |
| Ratner^220^ | 2023 | ASx | CEA | Low | Unclear | High | High | Int | High | High | Low | NA | NA |  |
| Reiff^221^ | 2022 | ASx | CEA | Low | Low | Low | Low | Low | High | Low | Low | High | Unclear |  |
| Reiff^221^ | 2022 | ASx | CAS | Low | Low | Low | Low | Low | High | Low | Low | High | Unclear |  |
| Reimers^222^ | 2004 | Sx | CAS | Low | Low | High | Low | Int | Low | Low | Low | NA | NA |  |
| Reimers^222^ | 2004 | ASx | CAS | Low | Low | High | Low | Int | Low | Low | Low | NA | NA |  |
| Reinert^223^ | 2012 | Sx | CEA | Low | Low | Low | Low | Low | Low | Low | Low | NA | NA |  |
| Reinert^223^ | 2012 | ASx | CEA | Low | Low | Low | Low | Low | Low | Low | Low | NA | NA |  |
| Reiter^224^ | 2006 | Sx | CAS | Low | Low | High | Low | Int | Low | Low | Low | NA | NA |  |
| Reiter^224^ | 2006 | ASx | CAS | Low | Low | High | Low | Int | Low | Low | Low | NA | NA |  |
| Reuter^225^ | 2004 | Sx | CEA | Low | Low | High | High | Int | Low | High | Low | NA | NA |  |
| Reuter^225^ | 2004 | ASx | CEA | Low | Low | High | High | Int | Low | High | Low | NA | NA |  |
| Rizwan^226^ | 2019 | Sx | CAS | Low | High | High | High | Int | Low | High | Low | NA | NA |  |
| Rizwan^226^ | 2019 | ASx | CAS | Low | High | High | High | Int | Low | High | Low | NA | NA |  |
| Rockman^227^ | 2006 | Sx | CEA | Low | Unclear | Low | Low | Low | Low | High | Low | NA | NA |  |
| Rosenfield^228^ | 2016 | ASx | CAS | Low | Low | Low | Low | Int | Low | Low | Low | NA | NA |  |
| Rothenberg^229^ | 2020 | Sx | CEA | Low | Unclear | Low | High | Int | High | High | High | Low | Unclear |  |
| Rothenberg^229^ | 2020 | ASx | CEA | Low | Unclear | Low | High | Int | High | High | High | Low | Unclear |  |
| Rothenberg^230^ | 2021 | Sx | CEA | Low | Unclear | Low | High | Low | Low | Int | Low | NA | NA |  |
| Rothenberg^230^ | 2021 | ASx | CEA | Low | Unclear | Low | High | Low | Low | Int | Low | NA | NA |  |
| Roussopoulou^231^ | 2019 | Sx | CEA | Low | Low | Low | Low | Low | Low | Low | High | NA | NA |  |
| Sabeti^232^ | 2004 | Sx | CAS | High | Low | Low | Low | Int | Low | Low | Low | NA | NA |  |
| Sakai^233^ | 2014 | Sx | CAS | Low | Low | Low | Low | Int | High | High | NA | Low | Unclear |  |
| Sakai^233^ | 2014 | ASx | CAS | Low | Low | Low | Low | Int | High | High | NA | Low | Unclear |  |
| Salem^234^ | 2011 | Sx | CEA | Low | Low | High | Low | Int | High | High | Low | NA | NA |  |
| Sayeed^235^ | 2008 | Sx | CAS | Low | Unclear | High | Low | Int | Low | Low | Low | NA | NA |  |
| Sayeed^235^ | 2008 | ASx | CAS | Low | Unclear | High | Low | Int | Low | Low | Low | NA | NA |  |
| Schillinger^236^ | 2008 | Sx | CAS | Low | Low | High | Low | Int | Low | Low | Low | NA | NA |  |
| Schillinger^236^ | 2008 | ASx | CAS | Low | Low | High | Low | Int | Low | Low | Low | NA | NA |  |
| Schmid^237^ | 2017 | Sx | CEA | Low | Unclear | High | High | Int | High | Low | Low | NA | NA |  |
| Schmid^237^ | 2017 | ASx | CEA | Low | Unclear | High | High | Int | High | Low | Low | NA | NA |  |
| Schoellhammer^238^ | 2018 | Sx | CEA | Low | Unclear | Low | Low | Int | Low | High | Low | Low | Low |  |
| Sef^239^ | 2018 | Sx | CEA | Low | Low | High | Low | Int | High | High | Low | NA | NA |  |
| Sef^239^ | 2018 | ASx | CEA | Low | Low | High | Low | Int | High | High | Low | NA | NA |  |
| Setacci^240^ | 2007 | Sx | CAS | Low | Low | High | High | Int | Low | High | Low | NA | NA |  |
| Setacci^240^ | 2007 | ASx | CAS | Low | Low | High | High | Int | Low | High | Low | NA | NA |  |
| Setacci^240^ | 2007 | Sx | CEA | Low | Low | High | High | Int | Low | High | Low | NA | NA |  |
| Setacci^240^ | 2007 | ASx | CEA | Low | Low | High | High | Int | Low | High | Low | NA | NA |  |
| Setacci^240^ | 2007 | Sx | CAS | Low | Low | High | High | Int | Low | High | Low | NA | NA |  |
| Setacci^240^ | 2007 | ASx | CAS | Low | Low | High | High | Int | Low | High | Low | NA | NA |  |
| Shahat^284^ | 2024 | Sx | CAS | Low | Low | High | Low | Int | Low | High | Low | NA | NA |  |
| Shahat^284^ | 2024 | ASx | CAS | Low | Low | High | Low | Int | Low | High | Low | NA | NA |  |
| Sharpe^241^ | 2013 | Sx | CEA | Low | Unclear | High | High | Int | Low | High | Low | NA | NA |  |
| Shobha^242^ | 2010 | ASx | CAS | Low | Unclear | Low | High | Int | Low | Low | Low | NA | NA |  |
| Siewiorek^243^ | 2011 | Sx | CAS | Low | High | High | High | Int | High | High | Low | NA | NA |  |
| Siewiorek^243^ | 2011 | ASx | CAS | Low | High | High | High | Int | High | High | Low | NA | NA |  |
| Simonetti^244^ | 2009 | Sx | CAS | Low | High | High | High | Int | High | Low | Low | NA | NA |  |
| Simonetti^244^ | 2009 | ASx | CAS | Low | High | High | High | Int | High | Low | Low | NA | NA |  |
| Sirignano^286^ | 2024 | Sx | CAS | Low | Unclear | Low | High | Int | Low | Low | Low | NA | NA |  |
| Sokol^245^ | 2011 | ASx | CEA | Low | Unclear | Low | High | Low | Low | High | Low | NA | NA |  |
| Spes^246^ | 2007 | Sx | CAS | Low | Low | High | Low | Int | Low | Low | Low | High | Unclear |  |
| Spes^246^ | 2007 | ASx | CAS | Low | Low | High | Low | Int | Low | Low | Low | High | Unclear |  |
| Squizzato^247^ | 2020 | ASx | CEA | Low | Low | Low | High | Int | Low | Int | Int | High | Unclear |  |
| Squizzato^247^ | 2020 | ASx | CEA | Low | Low | Low | High | Int | Low | Int | Int | High | Unclear |  |
| Squizzato^248^ | 2023 | ASx | CAS | Low | Unclear | Low | High | Int | High | Int | Low | NA | NA |  |
| Stabile^249^ | 2010 | Sx | CAS | Low | Low | High | Low | Int | Low | Low | Low | NA | NA |  |
| Stabile^249^ | 2010 | ASx | CAS | Low | Low | High | Low | Int | Low | Low | Low | NA | NA |  |
| Stanziale^250^ | 2006 | Sx | CAS | High | Unclear | High | Low | Int | Low | High | Low | NA | NA |  |
| Stanziale^250^ | 2006 | ASx | CAS | High | Unclear | High | Low | Int | Low | High | Low | NA | NA |  |
| Stelagowksi^251^ | 2017 | Sx | CEA | Low | Low | Low | Unclear | Int | High | Low | Low | NA | NA |  |
| Sternbach^252^ | 2000 | ASx | CEA | Low | Low | Low | High | High | Low | High | Low | NA | NA |  |
| Sztriha^253^ | 2004 | Sx | CAS | Int | Low | High | Unclear | Int | Low | Int | Low | NA | NA |  |
| Sztriha^253^ | 2004 | ASx | CAS | Low | Low | High | Unclear | Int | Low | Int | Low | NA | NA |  |
| Taboada^254^ | 2016 | Sx | CEA | Low | Unclear | High | High | Int | High | Low | Low | NA | NA |  |
| Taboada^254^ | 2016 | ASx | CEA | Low | Unclear | High | High | Int | High | Low | Low | NA | NA |  |
| Tang^255^ | 2008 | ASx | CEA | Low | Unclear | Low | High | Int | Low | Int | Low | NA | NA |  |
| Tang^255^ | 2008 | ASx | CAS | Low | Unclear | Low | High | Int | Low | Int | Low | NA | NA |  |
| Tan^256^ | 2003 | Sx | CAS | Low | Low | High | High | Int | Low | Int | Low | NA | NA |  |
| Tan^256^ | 2003 | ASx | CAS | Low | Low | High | High | Int | Low | Int | Low | NA | NA |  |
| Taurino^257^ | 2022 | Sx | CEA | Low | Unclear | Low | Low | Int | Low | High | Low | NA | NA |  |
| Teixeira^258^ | 2019 | Sx | CEA | Low | Low | High | Low | Int | Low | High | Low | NA | NA |  |
| Teixeira^258^ | 2019 | ASx | CEA | Low | Low | High | Low | Int | Low | High | Low | NA | NA |  |
| Teng^259^ | 2021 | Sx | CEA | Low | High | High | Low | Int | High | High | Int | NA | NA |  |
| Teng^259^ | 2021 | ASx | CEA | Low | High | High | Low | Int | High | High | Int | NA | NA |  |
| Tigkiropoulos^260^ | 2021 | Sx | CAS | Low | Low | High | Low | Int | High | High | Low | NA | NA |  |
| Tigkiropoulos^260^ | 2021 | ASx | CAS | Low | Low | High | Low | Int | High | High | Low | NA | NA |  |
| Till^261^ | 1987 | Sx | CEA | Low | Low | High | Low | High | High | Low | Low | NA | NA |  |
| Till^261^ | 1987 | ASx | CEA | Low | Low | High | Low | High | High | Low | Low | NA | NA |  |
| Timmerman^262^ | 2020 | Sx | CEA | Low | Low | Low | Low | Low | Low | High | Low | Low | Unclear |  |
| Tresson^287^ | 2024 | Sx | CEA | Low | Low | Low | High | Low | High | High | Low | High | High |  |
| Tresson^287^ | 2024 | ASx | CEA | Low | Low | Low | High | Low | High | High | Low | High | High |  |
| Tsivgoulis^263^ | 2014 | Sx | CEA | Low | Low | Low | Low | Int | Low | Low | Low | NA | NA |  |
| Tu^111^ | 2003 | Sx | CEA | Low | Low | High | High | High | Low | Int | Low | NA | NA |  |
| Tu^111^ | 2003 | ASx | CEA | Low | Low | High | High | High | Low | Int | Low | NA | NA |  |
| Ucci^264^ | 2023 | ASx | CEA | Low | High | Low | High | Int | High | High | Low | NA | NA |  |
| Ucci^265^ | 2018 | Sx | CEA | Low | Low | High | Low | Int | High | High | Low | NA | NA |  |
| Ucci^265^ | 2018 | ASx | CEA | Low | Low | High | Low | Int | High | High | Low | NA | NA |  |
| Uchida^280^ | 2024 | Sx | CAS | Low | Low | Low | High | Low | Low | High | Low | NA | NA |  |
| Uchida^280^ | 2024 | ASx | CAS | Low | Low | Low | High | Low | Low | High | Low | NA | NA |  |
| Velez^266^ | 2008 | Sx | CAS | High | Low | High | High | Int | Low | Low | Int | NA | NA |  |
| Velez^266^ | 2008 | ASx | CAS | High | Low | High | High | Int | Low | Low | Int | NA | NA |  |
| Veraldi^267^ | 2021 | Sx | CEA | Low | Low | High | Low | Int | High | Low | Low | NA | NA |  |
| Veraldi^267^ | 2021 | ASx | CEA | Low | Low | High | Low | Int | High | Low | Low | NA | NA |  |
| Viglas^288^ | 2024 | Sx | CAS | Low | Low | Low | High | Int | Low | Int | Low | NA | NA |  |
| Viglas^288^ | 2024 | ASx | CAS | Low | Low | Low | High | Int | Low | Int | Low | NA | NA |  |
| Wach^268^ | 2014 | Sx | CAS | Low | Low | Low | Low | Int | High | High | Low | NA | NA |  |
| Wallaert^269^ | 2013 | ASx | CEA | Low | High | High | High | Low | Low | High | NA | Low | Low |  |
| White^270^ | 2010 | Sx | CAS | Low | Unclear | High | Unclear | High | Low | High | Low | NA | NA |  |
| White^270^ | 2010 | ASx | CAS | Low | Unclear | High | Unclear | High | Low | High | Low | NA | NA |  |
| Winkler^271^ | 2006 | Sx | CEA | Low | Unclear | High | Unclear | Low | High | High | NA | High | Unclear |  |
| Winkler^271^ | 2006 | ASx | CEA | Low | Unclear | High | Unclear | Low | High | High | NA | High | Unclear |  |
| Wittkugel^272^ | 2008 | Sx | CAS | Low | Unclear | Low | High | Int | Low | Low | Low | NA | NA |  |
| Wong^273^ | 1999 | Sx | CEA | Low | Low | High | Low | Int | Low | High | Low | NA | NA |  |
| Wong^273^ | 1999 | ASx | CEA | Low | Low | High | Low | Int | Low | High | Low | NA | NA |  |
| Yoshida^275^ | 2013 | Sx | CEA | Low | Low | Low | High | Int | High | High | Low | NA | NA |  |
| Yoshida^275^ | 2013 | ASx | CEA | Low | Low | Low | High | Int | High | High | Low | NA | NA |  |
| Yoshida^275^ | 2013 | Sx | CAS | Low | Low | Low | High | Int | High | High | Low | NA | NA |  |
| Yoshida^275^ | 2013 | ASx | CAS | Low | Low | Low | High | Int | High | High | Low | NA | NA |  |
| Yu^274^ | 2016 | Sx | CAS | Low | Unclear | Low | High | Int | Low | High | Low | High | Low |  |
| Zahn^276^ | 2005 | Sx | CAS | Low | Unclear | High | Low | Int | Low | Low | Low | NA | NA |  |
| Zahn^276^ | 2005 | ASx | CAS | Low | Unclear | High | Low | Int | Low | Low | Low | NA | NA |  |
| Zarins^54^ | 2009 | Sx | CEA | Low | Unclear | High | Low | High | Low | High | Low | High | Unclear |  |
| Zarins^54^ | 2009 | ASx | CEA | Low | Unclear | High | Low | High | Low | High | Low | High | Unclear |  |
| Zarins^54^ | 2009 | Sx | CAS | Low | Unclear | High | Low | High | Low | High | Low | High | Unclear |  |
| Zarins^54^ | 2009 | ASx | CAS | Low | Unclear | High | Low | High | Low | High | Low | High | Unclear |  |
| Zharova^277^ | 2022 | Sx | CEA | Low | Unclear | Low | Low | High | High | High | Int | NA | NA |  |
| ASx: asymptomatic; CAS: carotid artery stenting; CEA: carotid endarterectomy; Int: intermediate; NA: not applicable; Sx: symptomatic. | | | | | | | | | | | | | |  |

## Figure S1. Time trends in age and sex in cohorts reporting long-term death after CEA

Graphs showing time trends in age and percentage of male sex in the 19 cohorts reporting long-term death after CEA for symptomatic stenosis, by midyear of treatment. Age increased 1.2 with each 5-y increase in midyear. The percentage of male patients decreased 1.3% with each 5-y increase in midyear.


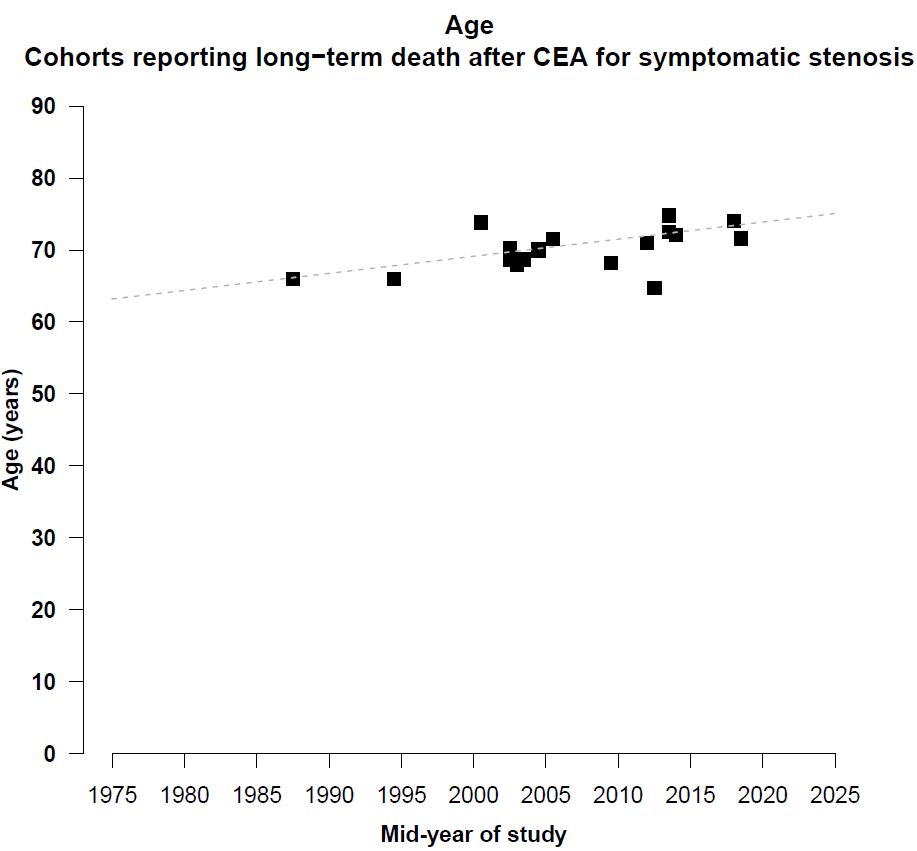

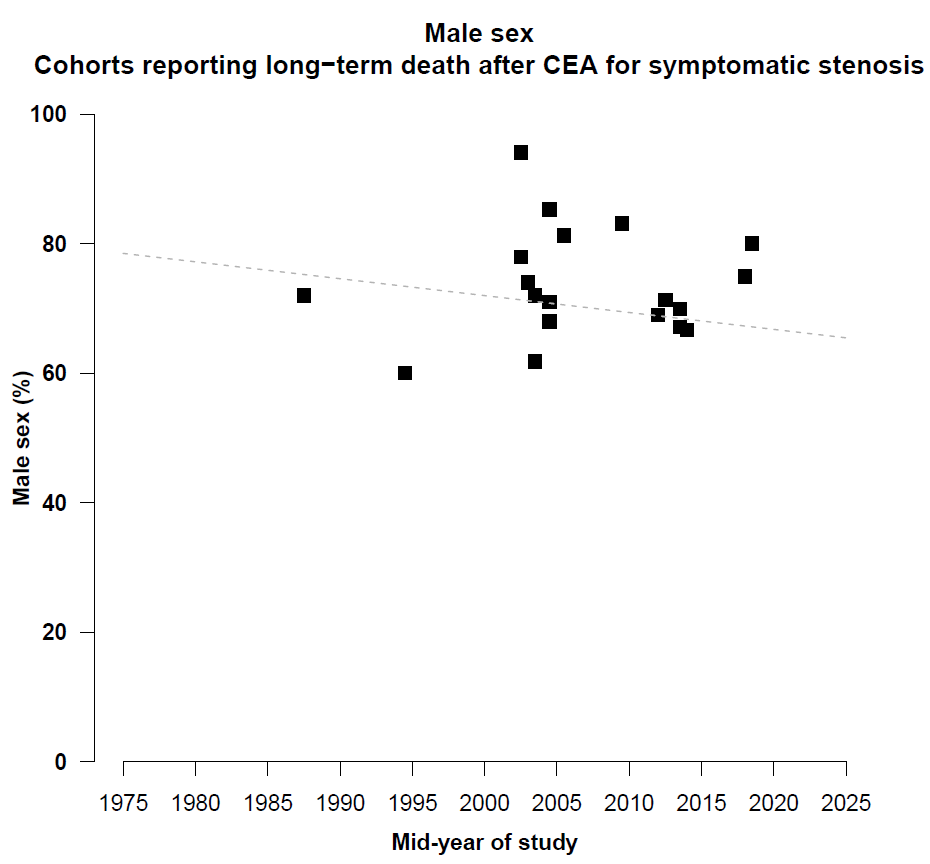


## Figure S2. Time trends in age and sex in cohorts reporting long-term stroke after CAS

Graphs showing time trends in age and percentage of male sex in the 16 cohorts reporting long-term stroke after CAS for symptomatic stenosis, by midyear of treatment. Age decreased 1.3 with each 5-y increase in midyear. The percentage of male patients increased 0.04% with each 5-y increase in midyear.


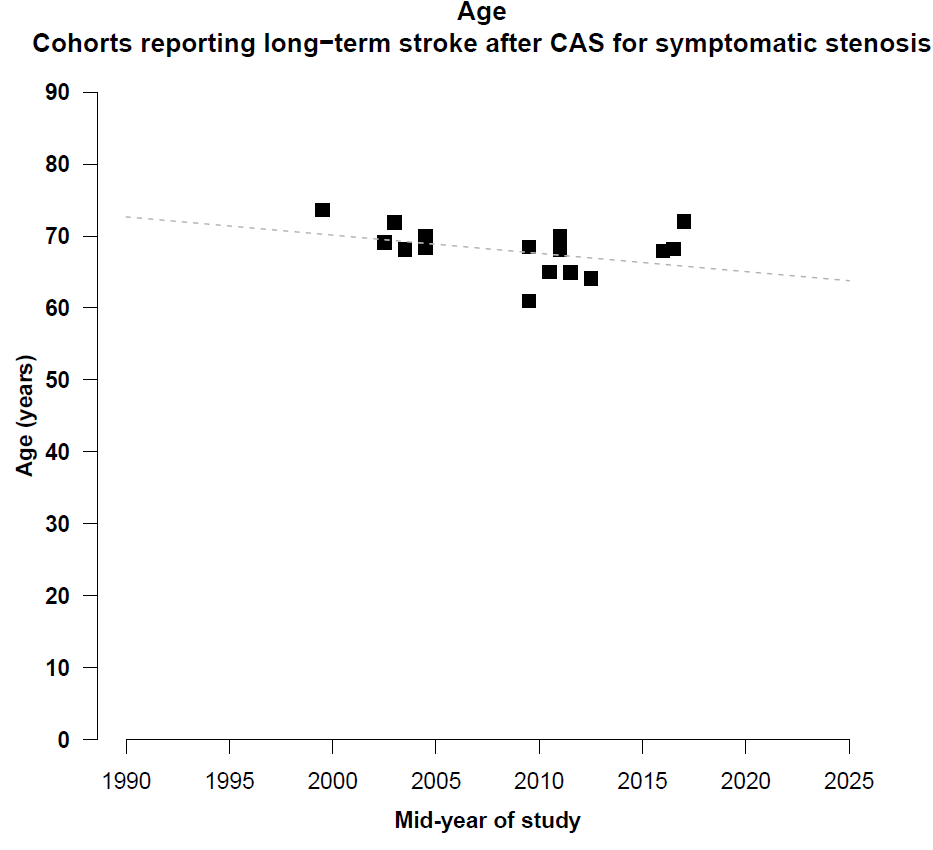

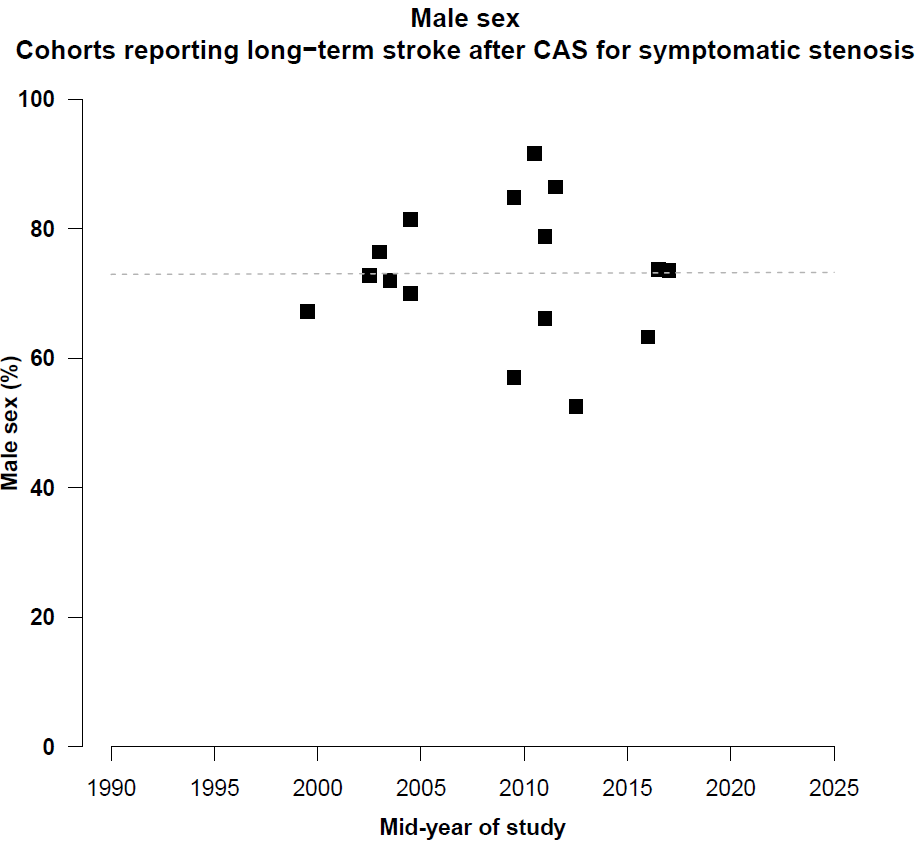


## Figure S3. Risk of bias assessment of included cohorts


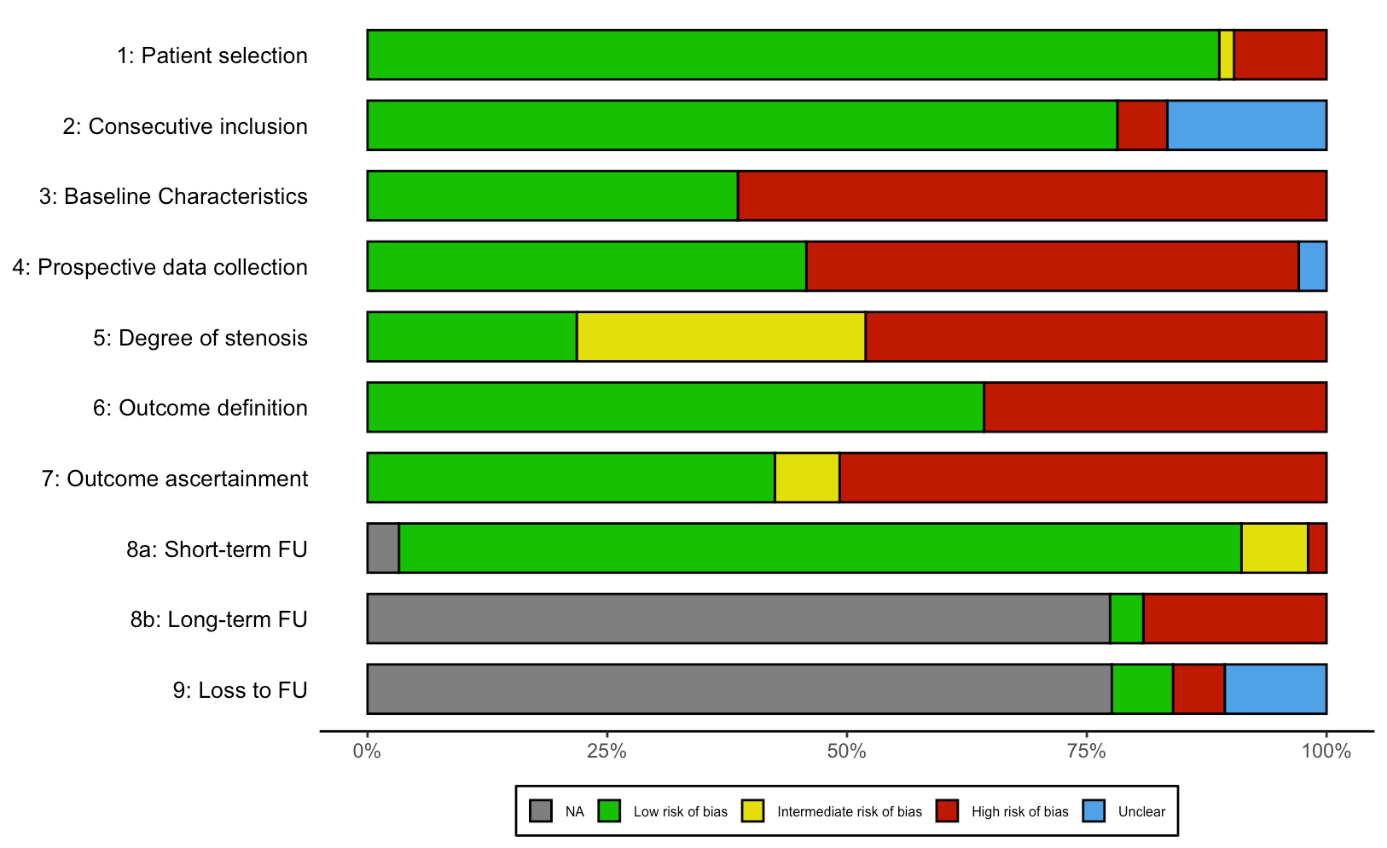


## Reference list of included articles

1. AbuRahma AF, Srivastava M, Hass SM, et al. Practice patterns of carotid endarterectomy as performed by different surgical specialties at a single institution and the effect on perioperative stroke and cost of preoperative imaging. *J Vasc Surg*. 2014;60(5):1232-1237. doi:10.1016/J.JVS.2014.04.068

2. AbuRahma AF, DerDerian T, Hariri N, et al. Anatomical and technical predictors of perioperative clinical outcomes after carotid artery stenting. *J Vasc Surg*. 2017;66(2):423-432. doi:10.1016/J.JVS.2017.02.057

3. Achim A, Lackó D, Hüttl A, et al. Impact of Diabetes Mellitus on Early Clinical Outcome and Stent Restenosis after Carotid Artery Stenting. *J Diabetes Res*. 2022;2022. doi:10.1155/2022/4196195

4. Adelman MA, Jacobowitz GR, Riles TS, et al. Carotid endarterectomy in the presence of a contralateral occlusion: a review of 315 cases over a 27-year experience. *Cardiovasc Surg*. 1995;3(3):307-312. doi:10.1016/0967-2109(95)93881-O

5. Angle N, Loja M, Angle A, Alam M, Gerstch JH. Outcomes of Preferential Early Carotid Endarterectomy following Recent Stroke. *Ann Vasc Surg*. 2022;83:26-34. doi:10.1016/j.avsg.2022.02.015

6. Alcalde-López J, Zapata-Arriaza E, Cayuela A, et al. Safety of Early Carotid Artery Stenting for Symptomatic Stenosis in Daily Practice. *Eur J Vasc Endovasc Surg*. 2018;56(6):776-782. doi:10.1016/j.ejvs.2018.07.026

7. Akkan K, Ilgit E, Onal B, et al. Endovascular Treatment for Near Occlusion of the Internal Carotid Artery : 30-Day Outcome and Long-Term Follow-Up. *Clin Neuroradiol*. 2018;28(2):245-252. doi:10.1007/s00062-016-0546-8

8. Almekhlafi MA, Couillard PL, Pandya A, et al. Outcomes after carotid angioplasty and stenting in symptomatic octogenarians. *Can J Neurol Sci*. 2011;38(3):446-451. doi:10.1017/s0317167100011859

9. Alvarez Gallesio JM, Ruiz PG, David M, Devoto M, Caride A, Borracci RA. Long-term outcomes of symptomatic and asymptomatic patients undergoing carotid endarterectomy in an average-volume community hospital. *Acta Chir Belg*. 2021;121(6):398-404. doi:10.1080/00015458.2020.1798112

10. Annambhotla S, Park MS, Keldahl ML, et al. Early versus delayed carotid endarterectomy in symptomatic patients. *J Vasc Surg*. 2012;56(5):1296-1302. doi:10.1016/J.JVS.2012.05.070

11. Ansel GM, Hopkins LN, Jaff MR, et al. Safety and effectiveness of the INVATEC MO.MA proximal cerebral protection device during carotid artery stenting: results from the ARMOUR pivotal trial. *Catheter Cardiovasc Interv*. 2010;76(1):1-8. doi:10.1002/CCD.22439

12. Appleberg M, Cottier D, Crozier J, Graham J, Lane R. Carotid endarterectomy for asymptomatic carotid artery stenosis: patients with severe bilateral disease a high risk subgroup*.* *Aust. N.Z. J. Surg.* Vol 65.; 1995. doi:10.1016/j.jvs.2014.04.068

13. Arhuidese IJ, Faateh M, Nejim BJ, Locham S, Abularrage CJ, Malas MB. Risks Associated With Primary and Redo Carotid Endarterectomy in the Endovascular Era. *JAMA Surg*. 2018;153(3):252-259. doi:10.1001/jamasurg.2017.4477

14. Arhuidese IJ, Rizwan M, Nejim B, Malas M. Outcomes of primary and secondary carotid artery stenting. *Stroke*. 2017;48(11):3086-3092. doi:10.1161/STROKEAHA.117.016963

15. Arslan S, Köklü E, Yüksel IÖ, et al. Two-year results of carotid artery stenting. *Turk Kardiyol Dern Ars*. 2014;42(5):429-434. doi:10.5543/tkda.2014.36825

16. Ascher E, Markevich N, Kallakuri S, Schutzer RW, Hingorani AP. Intraoperative carotid artery duplex scanning in a modern series of 650 consecutive primary endarterectomy procedures. *J Vasc Surg*. 2004;39(2):416-420. doi:10.1016/j.jvs.2003.09.019

17. Avgerinos ED, Go C, Ling J, Makaroun MS, Chaer RA. Survival and long-term cardiovascular outcomes after carotid endarterectomy in patients with chronic renal insufficiency. *Ann Vasc Surg*. 2015;29(1):15-21. doi:10.1016/j.avsg.2014.07.029

18. Baker WH, Howard VJ, Howard G, Toole JF. Effect of contralateral occlusion on long-term efficacy of endarterectomy in the asymptomatic carotid atherosclerosis study (ACAS). ACAS Investigators. *Stroke*. 2000;31(10):2330-2334. doi:10.1161/01.STR.31.10.2330

19. Baker DM, Jacob L, Busuttil A. A Stepwise Haemostasis Intraoperative Protocol Driven Reduction of Haematoma Rate Following Symptomatic Carotidendarterectomy. *Vasc Endovascular Surg*. 2023;57(2):154-158. doi:10.1177/15385744221132136

20. Ballotta E, Da Giau G. Selective shunting with eversion carotid endarterectomy. *J Vasc Surg*. 2003;38(5):1045-1050. doi:10.1016/s0741-5214(03)00605-0

21. Ballotta E, Meneghetti G, Da Giau G, Manara R, Saladini M, Baracchini C. Carotid endarterectomy within 2 weeks of minor ischemic stroke: a prospective study. *J Vasc Surg*. 2008;48(3):595-600. doi:10.1016/j.jvs.2008.04.044

22. Banga P V, Varga A, Csobay-Novák C, et al. Incomplete circle of Willis is associated with a higher incidence of neurologic events during carotid eversion endarterectomy without shunting. *J Vasc Surg*. 2018;68(6):1764-1771. doi:10.1016/j.jvs.2018.03.429

23. Barnett H, Taylor D, Eliasziw M, et al. Benefit of carotid endarterectomy in patients with symptomatic moderate or severe stenosis. North American Symptomatic Carotid Endarterectomy Trial Collaborators. *N Engl J Med*. 1998;339(20):274-275. doi:10.1056/NEJM199811123392002

24. Bazan HA, Caton G, Talebinejad S, et al. A stroke/vascular neurology service increases the volume of urgent carotid endarterectomies performed in a tertiary referral center. *Ann Vasc Surg*. 2014;28(5):1172-1177. doi:10.1016/j.avsg.2013.10.002

25. Baram A, Mohammed ZA, Al-Bajalan SJ, Falah F. Five-year outcome of non-shunting and primary closure technique during carotid endarterectomy: a longitudinal cohort study. *J Int Med Res*. 2022;50(4):3000605221076925. doi:10.1177/03000605221076925

26. Barnett H, Taylor D, Haynes R, et al. Beneficial effect of carotid endarterectomy in symptomatic patients with high-grade carotid stenosis. *N Engl J Med*. 1991;325(7):445-453. doi:10.1056/NEJM199108153250701

27. Barbetta I, Carmo M, Mercandalli G, et al. Outcomes of urgent carotid endarterectomy for stable and unstable acute neurologic deficits. *J Vasc Surg*. 2014;59(2):440-446. doi:10.1016/j.jvs.2013.08.035

28. Becquemin JP, Kadi HB El, Desgranges P, Kobeiter H. Carotid stenting versus carotid surgery: a prospective cohort study. *J Endovasc Ther*. 2003;10(4):687-694. doi:10.1177/152660280301000402

29. Ben Ahmed S, Daniel G, Benezit M, Ribal JP, Rosset E. Eversion carotid endarterectomy without shunt: concerning 1385 consecutive cases. *J Cardiovasc Surg (Torino)*. 2017;58(4):543-550. doi:10.23736/S0021-9509.16.08495-0

30. Bilas A, Cornette M. Retrospective evaluation of the carotid endarterectomies performed at CHBAH between 1987 and 2001. *Rev Med Liege*. 2003;58(7):493-500. doi:14579613

31. Bibl D, Lampl C, Biberhofer I, et al. Internal carotid artery stent placement without emboli protection: results and long-term outcome. *Neurology*. 2005;65(1):132-134. doi:10.1212/01.WNL.0000167604.56223.70

32. Biggs NG, Rangarajan S, Mcclure DN. Has carotid artery stenting found its place? A 10-year regional centre perspective. *ANZ J Surg*. 2016;86(3):179-183. doi:10.1111/ANS.12517

33. Binning MJ, Maxwell CR, Stofko D, et al. Carotid Artery Angioplasty and Stenting Without Distal Embolic Protection Devices. *Neurosurgery*. 2017;80(1):60-64. doi:10.1227/NEU.0000000000001367

34. Bissacco D, Catanese V, Fossati A, et al. Role of preoperative serum creatinine and estimated glomerular filtration rate values in asymptomatic patients undergoing carotid endarterectomy. *J Cardiovasc Surg (Torino)*. 2018;59(3):412-418. doi:10.23736/S0021-9509.17.08912-1

35. Blohmé L, Sandström V, Hellström G, Swedenborg J, Takolander R. Complications in carotid endarterectomy are predicted by qualifying symptoms and preoperative CT findings. *Eur J Vasc Endovasc Surg*. 1999;17(3):213-218. doi:10.1053/ejvs.1998.0743

36. Boitano LT, DeCarlo C, Schwartz MR, et al. Surgeon specialty significantly affects outcome of asymptomatic patients after carotid endarterectomy. *J Vasc Surg*. 2020;71(4):1242-1252. doi:10.1016/j.jvs.2019.04.489

37. Bonati LH, Dobson J, Featherstone RL, et al. Long-term outcomes after stenting versus endarterectomy for treatment of symptomatic carotid stenosis: The International Carotid Stenting Study (ICSS) randomised trial. *The Lancet*. 2015;385(9967):529-538. doi:10.1016/S0140-6736(14)61184-3

38. Bosiers M, Peeters P, Deloose K, Verbist J, Sprouse LR. Selection of treatment for patients with carotid artery disease: medication, carotid endarterectomy, or carotid artery stenting. *Vascular*. 2005;13(2):92-97. doi:10.1258/RSMVASC.13.2.92

39. Bosiers M, Scheinert D, Mathias K, Langhoff R, Mudra H, Diaz-Cartelle J. Carotid stenting with distal protection in high-surgical-risk patients: one-year results of the ASTI trial. *Cardiovasc Intervent Radiol*. 2015;38(2):295-303. doi:10.1007/S00270-014-1000-4

40. Bourke VC, Bourke BM, Beiles CB. Operative Factors Associated with the Development of New Brain Lesions During Awake Carotid Endarterectomy. *Eur J Vasc Endovasc Surg*. 2016;51(2):167-173. doi:10.1016/j.ejvs.2015.08.022

41. Bramucci A, Fontana A, Massoni CB, Vecchiati E, Freyrie A, Tusini N. Dual- vs single-layer stents for endovascular treatment of symptomatic and asymptomatic internal carotid artery stenosis. *Cardiovasc Revasc Med*. Published online 2023. doi:10.1016/j.carrev.2023.06.016

42. Branchereau A, Ede B, Magnan PE, Rosset E, Mathieu JP. Surgery for asymptomatic carotid stenosis: a study of three patient subgroups. *Ann Vasc Surg*. 1998;12(6):572-578. doi:10.1007/s100169900202

43. Brott TG, Hobson RW, Howard G, et al. Stenting versus Endarterectomy for Treatment of Carotid-Artery Stenosis. *N Eng J Med*. 2010;363(1):11-23. doi:10.1056/nejmoa0912321

44. Brown KE, Fanciullo DJ, Hicks T, et al. Carotid artery stenting compared to carotid endarterectomy performed exclusively in a veteran population: One center’s experience with midterm results. *Ann Surg*. 2008;248(1):110-116. doi:10.1097/SLA.0b013e318176c49d

45. Cacioppa LM, Pini R, Longhi M, et al. The Value of Carotid Endarterectomy as a Learning Tool for Trainees. *Ann Vasc Surg*. 2018;47:195-199. doi:10.1016/j.avsg.2017.08.024

46. Calvillo-King L, Xuan L, Zhang S, Tuhrim S, Halm EA. Predicting risk of perioperative death and stroke after carotid endarterectomy in asymptomatic patients: derivation and validation of a clinical risk score. *Stroke*. 2010;41(12):2786-2794. doi:10.1161/STROKEAHA.110.599019

47. Dellagrammaticas D, Lewis S, Colam B, Rothwell PM, Warlow CP, Gough MJ. Carotid endarterectomy in the UK: acceptable risks but unacceptable delays. *Clin Med (Lond)*. 2007;7(6):589-592. doi:10.7861/clinmedicine.7-6-589

48. de Donato G, Setacci C, Deloose K, Peeters P, Cremonesi A, Bosiers M. Long-term results of carotid artery stenting. *J Vasc Surg*. 2008;48(6):1431-1441. doi:10.1016/J.JVS.2008.07.012

49. De Blasis S, Pulli R, Di Domenico R, et al. Elective or Urgent Carotid Endarterectomy in Symptomatic Patients: Analysis Based on the Type and Timing of Neurological Symptoms. *Ann Vasc Surg*. 2023;90:7-16. doi:10.1016/j.avsg.2022.10.023

50. Coppi G, Moratto R, Ragazzi G, et al. Comparing Outcomes of Carotid Endarterectomy with International Benchmarks: Audit from an Italian Vascular Surgery Department. *Ital Heart J.* 2005;6(11).

51. Cohen JE, Gomori JM, Itshayek E, et al. Ischemic complications after tailored carotid artery stenting in different subpopulations with high-grade stenosis: feared but rare. *J Clin Neurosci*. 2015;22(1):189-194. doi:10.1016/j.jocn.2014.09.005

52. Claus D, Huppert P, Bauersachs R, Diegel H, Hedtmann G. Endovascular therapy of carotid artery stenosis: A prospective case study. *J Neurointerv Surg*. 2010;2(1):59-64. doi:10.1136/jnis.2009.000836

53. Chung BH, Heo SH, Park YJ, Kim YW, Woo SY, Kim DI. Comparative Analysis Using Propensity Score Matching Analysis: Primary Closure versus Patch Angioplasty During Carotid Endarterectomy. *Ann Vasc Surg*. 2020;62:166-172. doi:10.1016/j.avsg.2018.11.011

54. Zarins CK, White RA, Diethrich EB, Shackelton RJ, Siami FS, Sandra Siami F. Carotid Revascularization Using Endarterectomy or Stenting Systems (CaRESS): 4-Year Outcomes. *J. Endovasc Ther.* 2009 16(4): 397-409. www.jevt.org

55. Cho JS, Song S, Huh U, et al. Comparing carotid endarterectomy and carotid artery stenting: retrospective single-center analysis. *Ann Palliat Med*. 2022;11(11):3409-3416. doi:10.21037/apm-22-797

56. Chisci E, Pigozzi C, Troisi N, et al. Thirty-day neurologic improvement associated with early versus delayed carotid endarterectomy in symptomatic patients. *Ann Vasc Surg*. 2015;29(3):435-442. doi:10.1016/j.avsg.2014.08.028

57. Chiam PTL, Roubin GS, Iyer SS, et al. Carotid artery stenting in elderly patients: importance of case selection. *Catheter Cardiovasc Interv*. 2008;72(3):318-324. doi:10.1002/ccd.21620

58. Chaturvedi S, Matsumura JS, Gray W, Xu C, Verta P. Carotid artery stenting in octogenarians: Periprocedural stroke risk predictor analysis from the multicenter carotid acculink/accunet post approval trial to uncover rare events (capture 2) clinical trial. *Stroke*. 2010;41(4):757-764. doi:10.1161/STROKEAHA.109.569426

59. Charalampoudis P, Therasse A, Ferdin F. Carotid endarterectomy in a low volume vascular centre. *Acta Chir Belg*. 2011;111(6):364-365. doi:10.1080/00015458.2011.11680773

60. Cernetti C, Reimers B, Picciolo A, et al. Carotid artery stenting with cerebral protection in 100 consecutive patients: Immediate and two-year follow-up results. *Ital Heart J*. 2003;4(10):695-700.

61. Castro-Afonso LH, Nakiri GS, Monsignore LM, et al. Outcomes of carotid artery stenting at a high-volume Brazilian interventional neuroradiology center. *Clinics (Sao Paulo)*. 2015;70(3):180-184. doi:10.6061/clinics/2015(03)05

62. Casana R, Tolva VS, Jr OA, Malloggi C, Silani V, Parati G. Carotid artery stenting is safe and effective for symptomatic patients with acute coronary syndrome. *Catheter Cardiovasc Interv*. 2020;96(1):129-135. doi:10.1002/ccd.28445

63. Casana R, Bissacco D, Malloggi C, et al. Aortic arch types and postoperative outcomes after carotid artery stenting in asymptomatic and symptomatic patients. *Int Angiol*. 2020;39(6):485-491. doi:10.23736/S0392-9590.20.04494-6

64. Chisci E, Lazzeri E, Masciello F, et al. Timing to Carotid Endarterectomy Affects Early and Long Term Outcomes of Symptomatic Carotid Stenosis. *Ann Vasc Surg*. 2022;82:314-324. doi:10.1016/j.avsg.2021.10.071

65. De Rango P, Parlani G, Verzini F, et al. Long-term prevention of stroke: a modern comparison of current carotid stenting and carotid endarterectomy. *J Am Coll Cardiol*. 2011;57(6):664-671. doi:10.1016/J.JACC.2010.09.041

66. Rango P De, Simonte G, Farchioni L, et al. Safety of Carotid Revascularization in Symptomatic Patients with less than 70 Years. *Ann Vasc Surg*. 2016;32:73-82. doi:10.1016/J.AVSG.2015.10.024

67. Derdeyn CP, Chimowitz MI, Lynn MJ, et al. Aggressive medical treatment with or without stenting in high-risk patients with intracranial artery stenosis (SAMMPRIS): the final results of a randomised trial. *Lancet*. 2014;383(9914):333-341. doi:10.1016/S0140-6736(13)62038-3

68. Dorigo W, Pulli R, Barbanti E, et al. Carotid endarterectomy in patients with acute neurological symptoms: a case-control study. *Interact Cardiovasc Thorac Surg*. 2007;6(3):369-373. doi:10.1510/ICVTS.2006.137547

69. Dumont TM, Wach MM, Mokin M, et al. Perioperative complications after carotid artery stenting: a contemporary experience from the university at buffalo neuroendovascular surgery team. *Neurosurgery*. 2013;73(4):689-693; discussion 693-4. doi:10.1227/NEU.0000000000000077

70. Duschek N, Skrinjar E, Waldhör T, et al. N-terminal pro B-type natriuretic peptide (NT pro-BNP) is a predictor of long-term survival in male patients of 75 years and older with high-grade asymptomatic internal carotid artery stenosis. *J Vasc Surg*. 2011;53(5):1242-1250. doi:10.1016/j.jvs.2010.10.123

71. Eckstein HH, Ringleb P, Allenberg JR, et al. Results of the Stent-Protected Angioplasty versus Carotid Endarterectomy (SPACE) study to treat symptomatic stenoses at 2 years: a multinational, prospective, randomised trial. *Lancet Neurol*. 2008;7(10):893-902. doi:10.1016/S1474-4422(08)70196-0

72. International Carotid Stenting Study investigators, Ederle J, Dobson J, et al. Carotid artery stenting compared with endarterectomy in patients with symptomatic carotid stenosis (International Carotid Stenting Study): an interim analysis of a randomised controlled trial International Carotid Stenting Study investigators. *The Lancet*. 2010;375:985-997. doi:10.1016/S0140

73. Elmously A, Rich N, Lazar AN, et al. Outcomes of early transcarotid artery revascularization versus carotid endarterectomy after acute neurologic events. *J Vasc Surg*. 2022;76(3):760-768. doi:10.1016/j.jvs.2022.04.025

74. European Carotid Surgery Trialists’ Collaborative Group. Randomised trial of endarterectomy for recently symptomatic carotid stenosis: final results of the MRC European Carotid Surgery Trial (ECST). *The Lancet*. 1998;351:1379-1387.

75. Fanous AA, Natarajan SK, Jowdy PK, et al. High-Risk Factors in Symptomatic Patients Undergoing Carotid Artery Stenting With Distal Protection: Buffalo Risk Assessment Scale (BRASS). *Neurosurgery*. 2015;77(4):531-542; discussion 542-3. doi:10.1227/NEU.0000000000000871

76. Fearn SJ, McCollum CN. Shortening and reimplantation for tortuous internal carotid arteries. *J Vasc Surg*. 1998;27(5):936-939. doi:10.1016/s0741-5214(98)70275-7

77. Feasby TE, Kennedy J, Quan H, Girard L, Ghali WA. Real-world replication of randomized controlled trial results for carotid endarterectomy. *Arch Neurol*. 2007;64(10):1496-1500. doi:10.1001/archneur.64.10.1496

78. Ferrero E, Ferri M, Viazzo A, et al. Early carotid surgery in patients after acute ischemic stroke: is it safe? A retrospective analysis in a single center between early and delayed/deferred carotid surgery on 285 patients. *Ann Vasc Surg*. 2010;24(7):890-899. doi:10.1016/j.avsg.2010.03.014

79. Ferrero E, Ferri M, Viazzo A, et al. A retrospective study on early carotid endarterectomy within 48 hours after transient ischemic attack and stroke in evolution. *Ann Vasc Surg*. 2014;28(1):227-238. doi:10.1016/j.avsg.2013.02.015

80. Finocchi C, Gandolfo C, Carissimi T, M DS, Bertoglio C. Role of transcranial Doppler and stump pressure during carotid endarterectomy. *Stroke*. 1997;28(12):2448-2452. doi:10.1161/01.str.28.12.2448

81. Fornelli F, Sirignano P, Mansour W, et al. Could Gender Impact on Immediate and Long-term Carotid Artery Stenting Outcome? Insight from an Italian Single Center Experience. *Ann Vasc Surg*. 2021;76:342-350. doi:10.1016/J.AVSG.2021.04.014

82. Fortin W, Chaput M, Elkouri S, Beaudoin N, Blair JF. Carotid endarterectomy after systemic thrombolysis in a stroke population. *J Vasc Surg*. 2020;71(4):1254-1259. doi:10.1016/j.jvs.2019.05.061

83. García-Rodríguez R, Vega-Valdés P, Calleja-Puerta S, et al. Endovascular treatment of carotid stenosis: An experience in the hospital Universitario Central de Asturias. *Rev Neurol*. 2012;54(2).

84. Geisbüsch P, Katzen BT, Peña C, Benenati JF, Uthoff H. Bivalirudin used as alternative anticoagulant in carotid artery stenting: a single center observational study. *J Interv Cardiol*. 2012;25(2):197-202. doi:10.1111/j.1540-8183.2011.00684.x

85. Gensicke H, Van der Worp B, Nederkoorn P, et al. Ischemic Brain Lesions After Carotid Artery Stenting Increase Future Cerebrovascular Risk. *J Am Coll Cardiol*. 2015;65(6):521-529.

86. Golledge J, Cuming R, Beattie DK, Davies AH, Golledge J. Influence outcome of patient-related variables of carotid endarterectomy. *J Vasc Surg*. 1996;24(1):120-126.

87. Goode SD, Cleveland TJ, Gaines PA. United Kingdom carotid artery stent registry: short- and long-term outcomes. *Cardiovasc Intervent Radiol*. 2013;36(5):1221-1231. doi:10.1007/s00270-013-0573-7

88. Grant A, White C, Ansel G, Bacharach M, Metzger C, Velez C. Safety and efficacy of carotid stenting in the very elderly. *Catheter Cardiovasc Interv*. 2010;75(5):651-655. doi:10.1002/ccd.22345

89. Gray WA, Chaturvedi S, Verta P. Thirty-day outcomes for carotid artery stenting in 6320 patients from 2 prospective, multicenter, high-surgical-risk registries. *Circ Cardiovasc Interv*. 2009;2(3):159-166. doi:10.1161/CIRCINTERVENTIONS.108.823013

90. Gray WA, Hopkins LN, Yadav S, et al. Protected carotid stenting in high-surgical-risk patients: the ARCHeR results. *J Vasc Surg*. 2006;44(2):258-268. doi:10.1016/J.JVS.2006.03.044

91. Gray WA, Yadav JS, Verta P, et al. The CAPTURE registry: results of carotid stenting with embolic protection in the post approval setting. *Catheter Cardiovasc Interv*. 2007;69(3):341-348. doi:10.1002/CCD.21050

92. Grimm JC, Arhuidese I, Beaulieu RJ, et al. Surgeon’s 30-day outcomes supporting the carotid revascularization endarterectomy versus stenting trial. *JAMA Surg*. 2014;149(12):1314-1318. doi:10.1001/JAMASURG.2014.1762

93. Gröschel K, Knauth M, Ernemann U, Pilgram SM, Schnaudigel S, Kastrup A. Early treatment after a symptomatic event is not associated with an increased risk of stroke in patients undergoing carotid stenting. *Eur J Neurol*. 2008;15(1):2-5. doi:10.1111/j.1468-1331.2007.02002.x

94. Halliday A, Bulbulia R, Bonati LH, et al. Second asymptomatic carotid surgery trial (ACST-2): a randomised comparison of carotid artery stenting versus carotid endarterectomy. *The Lancet*. 2021;398(10305):1065-1073. doi:10.1016/S0140-6736(21)01910-3

95. Halliday A, Harrison M, Hayter E, et al. 10-year stroke prevention after successful carotid endarterectomy for asymptomatic stenosis (ACST-1): A multicentre randomised trial. *The Lancet*. 2010;376(9746):1074-1084. doi:10.1016/S0140-6736(10)61197-X

96. Halm EA, Chassin MR, Tuhrim S, et al. Revisiting the appropriateness of carotid endarterectomy. *Stroke*. 2003;34(6):1464-1471. doi:10.1161/01.STR.0000072514.79745.7D

97. Halm EA, Tuhrim S, Wang JJ, Rockman C, Riles TS, Chassin MR. Risk factors for perioperative death and stroke after carotid endarterectomy: results of the new york carotid artery surgery study. *Stroke*. 2009;40(1):221-229. doi:10.1161/STROKEAHA.108.524785

98. Hartmann A, Hupp T, Koch HC, et al. Prospective study on the complication rate of carotid surgery. *Cerebrovasc Dis*. 1999;9(3):152-156. doi:10.1159/000015945

99. Hause S, Schönefuß R, Assmann A, et al. Editor’s Choice - Relevance of Infarct Size, Timing of Surgery, and Peri-operative Management for Non-ischaemic Cerebral Complications After Carotid Endarterectomy. *Eur J Vasc Endovasc Surg*. 2022;63(2):268-274. doi:10.1016/j.ejvs.2021.09.044

100. Hernández-Fernández F, Parrilla G, García-Villalba B, et al. Comparison between proximal versus distal protection devices in 287 cases of carotid revascularization using angioplasty and stenting: periprocedure complications, morbidity, and mortality. *Cardiovasc Intervent Radiol*. 2014;37(3):639-645. doi:10.1007/S00270-013-0714-Z

101. Hicks CW, Nejim B, Aridi HD, Black JH, Malas MB. Transfemoral Carotid Artery Stents Should Be Used with Caution in Patients with Asymptomatic Carotid Artery Stenosis. *Ann Vasc Surg*. 2019;54:1-11. doi:10.1016/j.avsg.2018.10.001

102. Hobson R W, Lal BK, Chakhtoura E, et al. Carotid artery stenting: analysis of data for 105 patients at high risk. *J Vasc Surg*. 2003;37(6):1234-1239. doi:10.1016/s0741-5214(02)75448-7

103. Hobson RW, Weiss DG, Fields WS, et al. Efficacy of carotid endarterectomy for asymptomatic carotid stenosis. The Veterans Affairs Cooperative Study Group. *N Engl J Med*. 1993;328(4):221-227. doi:10.1056/NEJM199301283280401

104. Hoffmann M, Robbs J. Carotid endarterectomy after recent cerebral infarction. *Eur J Vasc Endovasc Surg*. 1999;18(1):6-10. doi:10.1053/EJVS.1999.0817

105. Hong JH, Kang J, Yeo MJ, et al. The 10-year trend of periprocedural complication following carotid artery stenting; single center experience. *Cardiovasc Intervent Radiol*. 2015;38(2):280-287. doi:10.1007/s00270-014-0917-y

106. Hong JH, Sohn SI, Kwak J, et al. Dose-Dependent Effect of Statin Pretreatment on Preventing the Periprocedural Complications of Carotid Artery Stenting. *Stroke*. 2017;48(7):1890-1894. doi:10.1161/STROKEAHA.117.016680

107. Ielasi A, Latib A, Godino C, et al. Clinical outcomes following protected carotid artery stenting in symptomatic and asymptomatic patients. *J Endovasc Ther*. 2010;17(3):298-307. doi:10.1583/09-2997.1

108. Illig KA, Zhang R, Tanski W, Benesch C, Sternbach Y, Green RM. Is the rationale for carotid angioplasty and stenting in patients excluded from NASCET/ACAS or eligible for ARCHeR justified? *J Vasc Surg*. 2003;37(3):575-581. doi:10.1067/mva.2003.79

109. Ishiguro T, Yoneyama T, Ishikawa T, et al. Perioperative and Long-term Outcomes of Carotid Endarterectomy for Japanese Asymptomatic Cervical Carotid Artery Stenosis: A Single Institution Study. *Neurol Med Chir (Tokyo)*. 2015;55(11):830-837. doi:10.2176/nmc.oa.2014-0398

110. Iwasaki M, Kuroda S, Nakayama N, et al. Clinical characteristics and outcomes in carotid endarterectomy for internal carotid artery stenosis in a Japanese population: 10-year microsurgical experience. *J Stroke Cerebrovasc Dis*. 2011;20(1):55-61. doi:10.1016/j.jstrokecerebrovasdis.2009.11.001

111. Tu J V., Wang H, Bowyer B, Green L, Fang J, Kucey D. Risk Factors for Death or Stroke After Carotid Endarterectomy: Observations From the Ontario Carotid Endarterectomy Registry. *Stroke*. 2003;34(11):2568-2573. doi:10.1161/01.STR.0000092491.45227.0F

112. Jalbert JJ, Nguyen LL, Gerhard-Herman MD, et al. Outcomes after carotid artery stenting in Medicare beneficiaries, 2005 to 2009. *JAMA Neurol*. 2015;72(3):276-286. doi:10.1001/jamaneurol.2014.3638

113. Jang SH, Kwon DH, Han MK, et al. Impact of statin pretreatment on the complications of carotid stenting in asymptomatic patients: observational study. *BMC Neurol*. 2021;21(1):75. doi:10.1186/s12883-021-02104-z

114. Johansson EP, Wester P. Delay from symptoms to carotid endarterectomy. *J Intern Med*. 2008;263(4):404-411. doi:10.1111/j.1365-2796.2007.01908.x

115. Jonsson M, Hammar K, Lindberg M, et al. Editor’s Choice – Nationwide Outcome Analysis of Primary Carotid Endarterectomy in Symptomatic Patients Depending on Closure Technique and Patch Type. *Eu J Vasc Endovasc Surg*. 2023;65(4):467-473. doi:10.1016/j.ejvs.2022.12.033

116. Jordan Jr WD, Voellinger DC, Doblar DD, Plyushcheva NP, Fisher WS, McDowell HA. Microemboli detected by transcranial Doppler monitoring in patients during carotid angioplasty versus carotid endarterectomy. *Cardiovasc Surg*. 1999;7(1):33-38. doi:10.1016/s0967-2109(98)00097-0

117. Joviliano EE, Ribeiro MS, Sobreira ML, et al. Short-Term Outcomes of Transfemoral Carotid Artery Stenting and Carotid Endarterectomy in Symptomatic Patients: Data from a Multicentric Prospective Registry in Brazil. *Ann Vasc Surg*. 2022;85:41-48. doi:10.1016/j.avsg.2022.04.053

118. Jonsson M, Gillgren P, Wanhainen A, Acosta S, Lindström D. Peri-procedural Risk with Urgent Carotid Artery Stenting: A Population based Swedvasc Study. *Eur J Vasc Endovasc Surg*. 2015;49(5):506-512. doi:10.1016/j.ejvs.2015.01.007

119. Kang J, Kim YW, Kim DI, Woo SY, Park YJ. Outcomes of Carotid Revascularization versus Optimal Medical Treatment Alone for Asymptomatic Carotid Stenosis: Inverse-Probability-of-Treatment Weighting Using Propensity Scores. *World Neurosurg*. 2021;146:e419-e430. doi:10.1016/j.wneu.2020.10.104

120. Karkos CD, Karamanos DG, Papazoglou KO, Demiropoulos FP, Papadimitriou DN, Gerassimidis TS. Thirty-day outcome following carotid artery stenting: A 10-year experience from a single center. *Cardiovasc Intervent Radiol*. 2010;33(1):34-40. doi:10.1007/s00270-009-9746-9

121. Karthaus EG, Vahl A, Kuhrij LS, et al. The Dutch Audit of Carotid Interventions: Transparency in Quality of Carotid Endarterectomy in Symptomatic Patients in the Netherlands. *Eur J Vasc Endovasc Surg*. 2018;56(4):476-485. doi:10.1016/j.ejvs.2018.05.030

122. Kashyap VS, King AH, Foteh MI, et al. A multi-institutional analysis of transcarotid artery revascularization compared to carotid endarterectomy. *J Vasc Surg*. 2019;70(1):123-129. doi:10.1016/j.jvs.2018.09.060

123. Kastrup A, Gröschel K, Schulz JB, Nägele T, Ernemann U. Clinical predictors of transient ischemic attack, stroke, or death within 30 days of carotid angioplasty and stenting. *Stroke*. 2005;36(4):787-791. doi:10.1161/01.STR.0000157585.01437.1f

124. Kastrup A, Skalej M, Krapf H, Nägele T, Dichgans J, Schulz JB. Early outcome of carotid angioplasty and stenting versus carotid endarterectomy in a single academic center. *Cerebrovasc Dis*. 2003;15(1-2):84-89. doi:10.1159/000067134

125. Katzen BT, Criado FJ, Ramee SR, et al. Carotid artery stenting with emboli protection surveillance study: Thirty-day results of the CASES-PMS study. *Catheter. Cardiovasc. Interv*. 2007;70(2):316-323. doi:10.1002/ccd.21222

126. Kawaguchi S, Sakaki T, Tsunoda S, et al. Strategies to improve the outcome of carotid endarterectomy. *Neurol Med Chir (Tokyo)*. 1994;34(2):86-90. doi:10.2176/NMC.34.86

127. Kazandjian C, Settembre N, Lareyre F, et al. Cerebral Infarct Topography and Early Outcome after Surgery for Symptomatic Carotid Stenosis: A Multicentre Study. *Cerebrovasc. Dis*. 2017;44(5-6):291-296. doi:10.1159/000479934

128. Keyhani S, Madden E, Cheng EM, et al. Risk Prediction Tools to Improve Patient Selection for Carotid Endarterectomy Among Patients With Asymptomatic Carotid Stenosis. *JAMA Surg*. 2019;154(4):336-344. doi:10.1001/jamasurg.2018.5119

129. Kharroubi A, Petit-Colau MD, Jouhannet C, et al. Endarterectomy with “systematized” resection anastomosis of carotid bulb, about 240 cases. *J Med Vasc*. 2020;45(5):260-267. doi:10.1016/j.jdmv.2020.07.005

130. Kibrik P, Stonko DP, Alsheekh A, et al. Association of carotid revascularization approach with perioperative outcomes based on symptom status and degree of stenosis among octogenarians. *J Vasc Surg*. 2022;76(3):769-777.e2. doi:10.1016/j.jvs.2022.04.027

131. Kim C, Lee J, Lee SJ, Yun WS. Intraoperative microembolic signals during carotid endarterectomy. *Ann Vasc Surg*. 2022;81:196-201. doi:10.1016/j.avsg.2021.09.028

132. Kimiagar I, Gur AY, Auriel E, Peer A, Sacagiu T, Bass A. Long-term follow-up of patients after carotid stenting with or without distal protective device in a single tertiary medical center. *Vasc Endovascular Surg*. 2012;46(7):536-541. doi:10.1177/1538574412457471

133. Kim Y, Lee S, Tanious A, et al. The Weekend Effect in Carotid Endarterectomy for Symptomatic Carotid Stenosis. *Vasc Endovascular Surg*. 2022;56(3):284-289. doi:10.1177/15385744211061901

134. King AH, Kim AH, Kwan S, et al. Elevated Neutrophil to Lymphocyte Ratio is Associated with Worse Outcomes after Carotid Endarterectomy in Asymptomatic Patients. *J Stroke Cerebrovasc Dis*. 2021;30(12):106120. doi:10.1016/j.jstrokecerebrovasdis.2021.106120

135. Kirchhoff F, Eckstein HH. Locoregional Anaesthesia and Intra-Operative Angiography in Carotid Endarterectomy: 16 Year Results of a Consecutive Single Centre Series. *Eur J Vasc Endovasc Surg*. 2023;65(2):223-232. doi:10.1016/j.ejvs.2022.10.002

136. Kjørstad KE, Baksaas ST, Bundgaard D, et al. Editor’s Choice - The National Norwegian Carotid Study: Time from Symptom Onset to Surgery is too Long, Resulting in Additional Neurological Events. *Eur J Vasc Endovasc Surg*. 2017;54(4):415-422. doi:10.1016/j.ejvs.2017.07.013

137. Knappich C, Kuehnl A, Tsantilas P, et al. The Use of Embolic Protection Devices Is Associated With a Lower Stroke and Death Rate After Carotid Stenting. *JACC Cardiovasc Interv*. 2017;10(12):1257-1265. doi:10.1016/j.jcin.2017.03.032

138. Köklü E, Arslan Ş, E SG, Bayar N, Avcı R, Özgünoğlu EC. Six-year outcomes of carotid artery stenting performed with multidisciplinary management in a single center. *Anatol J Cardiol*. 2021;25(6):385-394. doi:10.14744/AnatolJCardiol.2020.20420

139. Kouvelos G, Koutsoumpelis A, Arnaoutoglou E, et al. The effect of increasing operator experience on procedure-related characteristics in patients undergoing carotid artery stenting. *Vascular*. 2017;25(5):488-496. doi:10.1177/1708538117691431

140. Kretz B, Kazandjian C, Bejot Y, et al. Delay between symptoms and surgery for carotid artery stenosis: Modification of our practice. *Ann Vasc Surg*. 2015;29(3):426-434. doi:10.1016/j.avsg.2014.07.035

141. Kucey DS, Bowyer B, Iron K, Austin P, Anderson G, Tu J V. Determinants of outcome after carotid endarterectomy. *J Vasc Surg*. 1998;28(6):1051-1058. doi:10.1016/s0741-5214(98)70031-x

142. Lago A, Parkhutik V, Tembl JI, et al. Diabetes does not affect outcome of symptomatic carotid stenosis treated with endovascular techniques. *Eur Neurol*. 2013;69(5):263-269. doi:10.1159/000346000

143. Lahlouh A, Kiwan R, Mandzia J, Pandey S. A Simplified One-Size-Fits-All Approach to Carotid Stenting. *Can J Neurol Sci*. 2024; 51(2). doi:10.1017/cjn.2023.48

144. Lane JS, Shekherdimian S, Moore WS. Does female gender or hormone replacement therapy affect early or late outcome after carotid endarterectomy? *J Vasc Surg*. 2003;37(3):568-574. doi:10.1067/mva.2003.96

145. Langhoff R, Mudra H, Waliszewski M, et al. Outcome of carotid angioplasty with a novel open-cell carotid stent system. *Vasc Endovascular Surg*. 2014;48(4):317-324. doi:10.1177/1538574413520517

146. Latacz P, Simka M, Krzanowski M, et al. Mid-term and late results of endovascular treatment for symptomatic carotid artery stenosis under proximal protection. *Wideochirurgia I Inne Techniki Maloinwazyjne*. 2021;16(1):175-182. doi:10.5114/WIITM.2020.94519

147. Latz CA, Boitano LT, Wang LJ, et al. Perioperative outcomes for carotid revascularization on asymptomatic dialysis-dependent patients meet Society for Vascular Society guidelines. *J Vasc Surg*. 2021;74(1):195-202. doi:10.1016/j.jvs.2020.11.044

148. Lawaetz M, Sandholt B, Eilersen EN, et al. Low Risk of Neurological Recurrence while Awaiting Carotid Endarterectomy: Results From a Danish Multicentre Study. *Eur J Vasc Endovasc Surg*. 2021;62(2):160-166. doi:10.1016/j.ejvs.2021.04.016

149. Lee J, You JH, Oh SH, et al. Outcomes of Stenting versus Endarterectomy for Symptomatic Extracranial Carotid Stenosis: A Retrospective Multicenter Study in Korea. *Ann Vasc Surg*. 2019;54:185-192.e1. doi:10.1016/j.avsg.2018.04.044

150. Lepore MR, Sternbergh WC, Salartash K, Tonnessen B, Money SR. Influence of NASCET/ACAS trial eligibility on outcome after carotid endarterectomy. *J Vasc Surg*. 2001;34(4):581-586. doi:10.1067/mva.2001.118079

151. Leško N, Maretta M, Škorvánek M, et al. Predictors of outcome events and 6-year mortality after carotid endarterectomy and carotid stenting in patients with carotid artery stenosis. *Neurol Neurochir Pol*. 2021;55(1):67-73. doi:10.5603/PJNNS.a2020.0089

152. Levy BR, Waqas M, Monteiro A, et al. Not a trifecta: complementary use of carotid artery revascularization techniques in the era of hybrid neurosurgery. *J Neurosurg*. 2023;138(1):199-204. doi:10.3171/2022.4.JNS22420

153. Libman RB, Sacco RL, Shi T, Correll JW, Mohr JP. Outcome after carotid endarterectomy for asymptomatic carotid stenosis. *Surg Neurol*. 1994;41(6):443-449. doi:10.1016/0090-3019(94)90005-1

154. Lim S, Mora-Pinzon M, Park T, Yoon W, Crisostomo PR, Cho JS. Medical therapy does not confer stroke prevention for all patients: identification of high-risk patients with asymptomatic carotid stenosis is still needed. *Int Angiol*. 2019;38(5):372-380. doi:10.23736/S0392-9590.19.04143-9

155. Lindström D, Jonsson M, Formgren J, Delle M, Rosfors S, Gillgren P. Outcome after 7 years of carotid artery stenting and endarterectomy in Sweden - Single centre and national results. Eur J Vasc Endovasc Surg. 2012;43(5):499-503. doi:10.1016/j.ejvs.2012.01.024

156. Liu H, Chu J, Zhang L, Liu C, Yan Z, Zhou S. Clinical comparison of outcomes of early versus delayed carotid artery stenting for symptomatic cerebral watershed infarction due to stenosis of the proximal internal carotid artery. *Biomed Res Int*. 2016. ;2016. doi:10.1155/2016/6241546

157. Liu YE, Zhu X, Ma Y, Tang H, Jin M. Age and Five-Year Outcomes After Carotid Artery Stenting in Symptomatic Carotid Stenosis: A Retrospective Cohort Study. *Vasc Endovascular Surg*. 2023;57(4):317-323. doi:10.1177/15385744221145147

158. Loftus IM, Paraskevas KI, Johal A, et al. Editor’s Choice – Delays to Surgery and Procedural Risks Following Carotid Endarterectomy in the UK National Vascular Registry. *European Journal of Vascular and Endovascular Surgery*. 2016;52(4):438-443. doi:10.1016/j.ejvs.2016.05.031

159. Longo GM, Icibbe MR, Eskandari MI. Carotid Artery Stenting in Octogenarians: Is It Too Risky? 2005. 19(6) doi:10.1007/~10016-005-7977

160. Lübke T, Ahmad W, B KJ, Brunkwall J. Gender-based 30-day and long-term outcomes after carotid endarterectomy. *Vasa*. 2015;44(4):289-295. doi:10.1024/0301-1526/a000444

161. Lutz HJ, Michael R, Gahl B, Savolainen H. Local versus General Anaesthesia for Carotid Endarterectomy - Improving the Gold Standard ? *European Journal of Vascular and Endovascular Surgery*. 2008;36(2):145-149. doi:10.1016/j.ejvs.2008.03.009

162. Madden NJ, Calligaro KD, Dougherty MJ, Maloni K, Troutman DA. Completion Arteriogram Following Carotid Endarterectomy Yields Lower Perioperative Stroke Rate. *Vasc Endovascular Surg*. 2022;56(1):29-32. doi:10.1177/15385744211048310

163. Madhani SI, Alvi MA, Pando A, et al. Thirty-Day Stroke and Mortality After Carotid Revascularization Among Octogenarians with Symptomatic Carotid Stenosis: Real-World Evidence from a National Surgical Quality Registry. *World Neurosurg*. 2022;167:e40-e52. doi:10.1016/j.wneu.2022.07.056

164. Mallela DP, Canner JK, Zarkowsky DS, Haut ER, Abularrage CJ, Hicks CW. Association between Race and Perioperative Outcomes after Carotid Endarterectomy for Asymptomatic Carotid Artery Stenosis in NSQIP. *J Am Coll Surg*. 2022;234(1):65-73. doi:10.1097/XCS.0000000000000016

165. Mallick D, Holscher CM, Canner JK, Zarkowsky DS, Abularrage CJ, Hicks CW. Sex does not have an impact on perioperative transfemoral carotid artery stenting outcomes among octogenarians. *J. Vasc Surg.* 2020. 72(4):1405-1412. doi:10.1016/j.jvs.2019.12.034

166. Mannheim D, Karmeli R. A prospective randomized trial comparing endarterectomy to stenting in severe asymptomatic carotid stenosis. *J Card Surg*. 2017;58(6):814-817. doi:10.23736/S0021-9509.16.09513-6

167. Mansour OY, Weber J, Niesen W, Schumacher M, Berlis A. Carotid angioplasty and stenting without protection devices: safety and efficacy concerns--single center experience. *Clin Neuroradiol*. 2011;21(2):65-73. doi:10.1007/s00062-011-0057-6

168. Macharzina RR, Müller C, Vogt M, et al. The SAPPHIRE criteria, history of myocardial infarction and diabetes predict adverse outcomes following carotid endarterectomy similar to stenting. *Clin Res Cardiol*. 2020;109(5):589-598. doi:10.1007/s00392-019-01546-3

169. Marine LA, Rubin BG, Reddy R, Sanchez LA, Parodi JC, Sicard GA. Treatment of asymptomatic carotid artery disease: Similar early outcomes after carotid stenting for high-risk patients and endarterectomy for standard-risk patients. *J Vasc Surg*. 2006;43(5):953-958. doi:10.1016/j.jvs.2006.01.008

170. Martín-Morales E, Jiménez-Román R, Paluso-Montero A, Hernández-Ruiz T, Mendieta-Azcona C, Moral LFR. Results and complications of carotid endarterectomy in a hospital from Madrid, Spain. *Cir Cir*. 2019;87(5):501-507. doi:10.24875/CIRU.19000529

171. Mas JL, Arquizan C, Calvet D, et al. Long-term follow-up study of endarterectomy versus angioplasty in patients with symptomatic severe carotid stenosis trial. *Stroke*. 2014;45(9):2750-2756. doi:10.1161/STROKEAHA.114.005671

172. Mastrorilli D, D’Oria M, Lepidi S, et al. Prediction of long-term mortality for patients with severe asymptomatic de novo carotid stenosis undergoing carotid endarterectomy (PREMY(2)SE-CEA): Derivation and validation of a novel risk score. *J Vasc Surg*. 2023;77(3):804-810.e3. doi:10.1016/j.jvs.2022.10.011

173. Mastrorilli D, Mezzetto L, D’Oria M, et al. National Institutes of Health stroke scale score at admission can predict functional outcomes in patients with ischemic stroke undergoing carotid endarterectomy. *J Vasc Surg*. 2022;75(5):1661-1669.e2. doi:10.1016/j.jvs.2021.11.079

174. Mazzaccaro D, Occhiuto MT, Stegher S, et al. Long-term results of carotid artery stenting in patients 80 years and older. *Perspect Vasc Surg Endovasc Ther*. 2012;24(2):49-54. doi:10.1177/1531003512459913

175. McCrory DC, Goldstein LB, Samsa GP, et al. Predicting complications of carotid endarterectomy. *Stroke*. 1993;24(9):1285-1291. doi:10.1161/01.str.24.9.1285

176. McKevitt FM, Macdonald S, Venables GS, Cleveland TJ, Gaines PA. Complications following carotid angioplasty and carotid stenting in patients with symptomatic carotid artery disease. *Cerebrovasc Dis*. 2004;17(1):28-34. doi:10.1159/000073895

177. Mehta RH, Zahn R, Hochadel M, et al. Effectiveness and safety of carotid artery stenting for significant carotid stenosis in patients with contralateral occlusion (from the German ALKK-CAS Registry experience). *Am J Cardiol*. 2009;104(5):725-731. doi:10.1016/j.amjcard.2009.04.038

178. Meller SM, M SAD, Gutierrez A, Stilp E, Mena-Hurtado C. Carotid stenting versus endarterectomy for the treatment of carotid artery stenosis: Contemporary results from a large single center study. *Catheter Cardiovasc Interv*. 2016;88(5):822-830. doi:10.1002/ccd.26593

179. Merlini T, Péret M, Lhommet P, et al. Is early surgical revascularization of symptomatic carotid stenoses safe? *Ann Vasc Surg*. 2014;28(6):1539-1547. doi:10.1016/j.avsg.2014.01.025

180. Meyer FB, Meissner I, Fode NC, Losasso TJ. Carotid endarterectomy in elderly patients. *Mayo Clin Proc*. 1991;66(5):464-469. doi:10.1016/s0025-6196(12)62385-4

181. Micari A, Stabile E, Cremonesi A, et al. Carotid artery stenting in octogenarians using a proximal endovascular occlusion cerebral protection device: a multicenter registry. *Catheter Cardiovasc Interv*. 2010;76(1):9-15. doi:10.1002/ccd.22503

182. Micheel A, Konietschke F, Hinterseher I, et al. Perioperative risk prediction using the POSSUM and V-POSSUM models in symptomatic carotid stenosis. *Vasa*. 2022;51(3):150-157. doi:10.1024/0301-1526/a000997

183. Middleton S, Donnelly N, Ward J. Outcomes of carotid endarterectomy: how does the Australian state of New South Wales compare with international benchmarks? *J Vasc Surg*. 2002;36(1):62-69. doi:10.1067/MVA.2002.123088

184. Miyachi S, Taki W, Sakai N, Nakahara I. Historical perspective of carotid artery stenting in Japan: analysis of 8,092 cases in The Japanese CAS survey. *Acta Neurochir (Wien)*. 2012;154(12):2127-2137. doi:10.1007/s00701-012-1508-9

185. Mo D, Wang B, Ma N, Gao F, Miao Z. Comparative outcomes of carotid artery stenting for asymptomatic and symptomatic carotid artery stenosis: a single-center prospective study. *J Neurointerv Surg*. 2016;8(2):126-129. doi:10.1136/NEURINTSURG-2014-011437

186. Montorsi P, Galli S, Ravagnani P, et al. Randomized trial of predilation versus direct stenting for treatment of carotid artery stenosis. *Int J Cardiol*. 2010;138(3):233-238. doi:10.1016/j.ijcard.2008.08.012

187. Morales-Gisbert SM, Zaragozá García JM, Plaza Martínez A, Gómez Palonés FJ, Ortiz-Monzón E. Development of an individualized scoring system to predict mid-term survival after carotid endarterectomy. *J Cardiovasc Surg (Torino)*. 2017;58(4):535-542. doi:10.23736/S0021-9509.16.08198-2

188. Murtidjaja M, Stathis AO, Thomas SD, et al. Trends and outcomes in Australian carotid artery revascularization surgery: 2010-2017. *ANZ J Surg*. 2021;91(6):1203-1210. doi:10.1111/ans.16757

189. Musialek P, Mazurek A, Trystula M, et al. Novel PARADIGM in carotid revascularisation: Prospective evaluation of All-comer peRcutaneous cArotiD revascularisation in symptomatic and Increased-risk asymptomatic carotid artery stenosis using CGuard^TM^ MicroNet-covered embolic prevention stent system. *EuroIntervention*. 2016;12(5):e658-70. doi:10.4244/EIJY16M05_02

190. Mutirangura P, Ruengsethakit C, Wongwanit C, et al. Carotid Endarterectomy in Symptomatic Extracranial Internal Carotid Artery Stenosis: A Result of the First 100 Consecutive Cases in a University Hospital. *J Med Assoc Thai*. 2016;99(7):785-793.

191. Nakagawa I, Park HS, Kotsugi M, et al. Elective carotid stenting after urgent best medical treatment suppresses recurrent stroke in patients with symptomatic carotid artery severe stenosis. *Clin Neurol Neurosurg*. 2020;195. doi:10.1016/J.CLINEURO.2020.105855

192. Ngo HTN, Nemeth B, Wever JJ, et al. Clinical outcomes of postcarotid endarterectomy hypertension. *J Vasc Surg*. 2020;71(2):553-559. doi:10.1016/j.jvs.2019.04.477

193. Nikas D, Reith W, Schmidt A, et al. Prospective, multicenter European study of the GORE flow reversal system for providing neuroprotection during carotid artery stenting. *Catheter Cardiovasc Interv*. 2012;80(7):1060-1068. doi:10.1002/ccd.24402

194. Nordanstig A, Rosengren L, Strömberg S, et al. Editor’s Choice - Very Urgent Carotid Endarterectomy is Associated with an Increased Procedural Risk: The Carotid Alarm Study. *Eur J Vasc Endovasc Surg*. 2017;54(3):278-286. doi:10.1016/j.ejvs.2017.06.017

195. Nowakowski P, Uchto W, Ziaja D, Nowakowska I, Kobayashi A, Pieniążek P. OCEANUS (PrOspective multiCentEr Study of cArotid Artery steNting Using mer Stent) Study: 30 Day and Two Year Results. *Eur J Vasc Endovasc Surg*. 2021;61(1):167-168. doi:10.1016/J.EJVS.2020.08.039

196. Ogata A, Sonobe M, Kato N, et al. Carotid artery stenting without post-stenting balloon dilatation. *J Neurointerv Surg*. 2014;6(7):517-520. doi:10.1136/neurintsurg-2013-010873

197. Ouriel K, Hertzer NR, Beven EG, et al. Preprocedural risk stratification: identifying an appropriate population for carotid stenting. *J Vasc Surg*. 2001;33(4):728-732. doi:10.1067/mva.2001.111981

198. Papakostas JC, Avgos S, Arnaoutoglou E, et al. Use of the vascu-guard bovine pericardium patch for arteriotomy closure in carotid endarterectomy. Early and long-term results. *Ann Vasc Surg*. 2014;28(5):1213-1218. doi:10.1016/j.avsg.2013.10.021

199. Pascot R, Parat B, Y LT, et al. Predictive Factors of Silent Brain Infarcts after Asymptomatic Carotid Endarterectomy. *Ann Vasc Surg*. 2018;51:225-233. doi:10.1016/j.avsg.2018.02.037

200. Paukovits TM, Haász J, Molnár A, et al. Transfemoral endovascular treatment of proximal common carotid artery lesions: a single-center experience on 153 lesions. *J Vasc Surg*. 2008;48(1):80-87. doi:10.1016/j.jvs.2008.03.008

201. Perini P, Bonifati DM, Tasselli S, Sogaro F. Routine Shunting During Carotid Endarterectomy in Patients With Acute Watershed Stroke. *Vasc Endovascular Surg*. 2017;51(5):288-294. doi:10.1177/1538574417708130

202. Perona F, Castellazzi G, Valvassori L, et al. Safety of unprotected carotid artery stent placement in symptomatic and asymptomatic patients: a retrospective analysis of 30-day combined adverse outcomes. *Radiology*. 2009;250(1):178-183. doi:10.1148/radiol.2493080057

203. Petkoska D, Zafirovska B, Vasilev I, Novotni G, Bertrand OF, Kedev S. Radial and ulnar approach for carotid artery stenting with Roadsaver^TM^ double layer micromesh stent: Early and long-term follow-up. *Catheter Cardiovasc Interv*. 2023;101(1):154-163. doi:10.1002/ccd.30514

204. Piazza M, Squizzato F, Chincarini C, et al. Quantitative analysis and predictors of embolic filter debris load during carotid artery stenting in asymptomatic patients. *J Vasc Surg*. 2018;68(1):109-117. doi:10.1016/j.jvs.2017.09.055

205. Pieniążek P, Tekieli Ł, Musiałek P, et al. Carotid artery stenting according to the “tailored−CAS” algorithm is associated with a low complication rate at 30 days: data from the TARGET−CAS study. *Kardiol Pol*. 2012;70:378-386.

206. Pini R, Faggioli G, Indelicato G, et al. Predictors and Consequences of Silent Brain Infarction in Patients with Asymptomatic Carotid Stenosis. *J Stroke Cerebrovasc Dis*. 2020;29(10):105108. doi:10.1016/j.jstrokecerebrovasdis.2020.105108

207. Pini R, Faggioli G, Longhi M, et al. Impact of acute cerebral ischemic lesions and their volume on the revascularization outcome of symptomatic carotid stenosis. *J Vasc Surg*. 2017;65(2):390-397. doi:10.1016/j.jvs.2016.08.077

208. Pini R, Faggioli G, Mauro R, et al. Chronic oral anticoagulant therapy in carotid artery stenting: the un-necessity of perioperative bridging heparin therapy. *Thromb Res*. 2012;130(1):12-15. doi:10.1016/j.thromres.2011.09.031

209. Poisson SN, Johnston SC, Sidney S, Klingman JG, Nguyen-Huynh MN. Gender differences in treatment of severe carotid stenosis after transient ischemic attack. *Stroke*. 2010;41(9):1891-1895. doi:10.1161/STROKEAHA.110.580977

210. Poorthuis MHF, Herings RAR, Dansey K, et al. External Validation of Risk Prediction Models to Improve Selection of Patients for Carotid Endarterectomy. *Stroke*. 2022;53(1):87-99. doi:10.1161/STROKEAHA.120.032527

211. Poulias GE, Doundoulakis N, Skoutas B, Haddad H, Karkanias G, Papadakis E. *Carotid Artery Surgery and the Principle of Prophylaxis: Recurrence in Operated and Non-Operated Patients*.; 1994.

212. Quispe-Orozco D, Limaye K, Zevallos CB, et al. Safety and efficacy of symptomatic carotid artery stenting performed in an emergency setting. *Interv Neuroradiol*. 2021;27(3):411-418. doi:10.1177/1591019920977552

213. Qumsiyeh Y, Siada S, Yan Y, et al. Carotid endarterectomy is safe for octogenarians. *J Vasc Surg*. 2023;77(1):176-181. doi:10.1016/j.jvs.2022.07.169

214. Radu H, Bertog SC, Robertson G, et al. Long-term results after carotid stent implantation. *J Interv Cardiol*. 2013;26(6):613-622. doi:10.1111/joic.12077

215. Randall MS, McKevitt FM, Kumar S, et al. Long-term results of carotid artery stents to manage symptomatic carotid artery stenosis and factors that affect outcome. *Circ Cardiovasc Interv*. 2010;3(1):50-56. doi:10.1161/CIRCINTERVENTIONS.108.828335

216. Rantner B, Eckstein HH, Ringleb P, et al. American Society of Anesthesiology and Rankin as Predictive Parameters for the Outcome of Carotid Endarterectomy Within 28 Days After an Ischemic Stroke. *J Stroke Cerebrovasc Dis*. 2006;15(3):114-120. doi:10.1016/j.jstrokecerebrovasdis.2006.03.004

217. Rantner B, Schmidauer C, Knoflach M, Fraedrich G. Very urgent carotid endarterectomy does not increase the procedural risk. *Eur J Vasc Endovasc Surg*. 2015;49(2):129-136. doi:10.1016/J.EJVS.2014.09.006

218. Rašiová M, Špak Ľ, Farkašová Ľ, et al. Remote pre-procedural ischemic stroke as the greatest risk in carotid‑stenting‑associated stroke and death: a single center’s experience. *Int Angiol*. 2017;36(4):306-315. doi:10.23736/S0392-9590.16.03737-8

219. Rathenborg LK, Jensen LP, Baekgaard N, Schroeder T V. Carotid endarterectomy after intravenous thrombolysis for acute cerebral ischaemic attack: is it safe? *Eur J Vasc Endovasc Surg*. 2013;45(6):573-577. doi:10.1016/j.ejvs.2013.02.020

220. Ratner M, Garg K, Chang H, et al. Young patients undergoing carotid endarterectomy have increased rates of recurrent disease and late neurological events. *J Vasc Surg*. 2023;78(1):123-130. doi:10.1016/j.jvs.2023.02.011

221. Reiff T, Eckstein HH, Mansmann U, et al. Carotid endarterectomy or stenting or best medical treatment alone for moderate-to-severe asymptomatic carotid artery stenosis: 5-year results of a multicentre, randomised controlled trial. *Lancet Neurol*. 2022;21(10):877-888. doi:10.1016/S1474-4422(22)00290-3

222. Reimers B, Schlüter M, Castriota F, et al. Routine use of cerebral protection during carotid artery stenting: results of a multicenter registry of 753 patients. *Am J Med*. 2004;116(4):217-222. doi:10.1016/j.amjmed.2003.09.043

223. Reinert M, Mono ML, Kuhlen D, et al. Restenosis after microsurgical non-patch carotid endarterectomy in 586 patients. *Acta Neurochir (Wien)*. 2012;154(3):423-431; discussion 431. doi:10.1007/s00701-011-1233-9

224. Reiter M, Bucek RA, Effenberger I, et al. Plaque echolucency is not associated with the risk of stroke in carotid stenting. *Stroke*. 2006;37(9):2378-2380. doi:10.1161/01.STR.0000237087.86583.C8

225. Reuter NP, Charette SD, Sticca RP. Cerebral protection during carotid endarterectomy. *Am J Surg*. 2004;188(6):772-777. doi:10.1016/J.AMJSURG.2004.08.037

226. Rizwan M, Faateh M, Dakour-Aridi H, Nejim B, Alshwaily W, Malas MB. Statins reduce mortality and failure to rescue after carotid artery stenting. *J Vasc Surg*. 2019;69(1):112-119. doi:10.1016/j.jvs.2018.03.424

227. Rockman CB, Maldonado TS, Jacobowitz GR, Cayne NS, Gagne PJ, Riles TS. Early carotid endarterectomy in symptomatic patients is associated with poorer perioperative outcomes. *J Vasc Surg*. 2006;44(3):480-487. doi:10.1016/j.jvs.2006.05.022

228. Rosenfield K, Matsumura JS, Chaturvedi S, et al. Randomized Trial of Stent versus Surgery for Asymptomatic Carotid Stenosis. *N Engl J Med*. 2016;374(11):1011-1020. doi:10.1056/nejmoa1515706

229. Rothenberg KA, George EL, Barreto N, et al. Frailty as measured by the Risk Analysis Index is associated with long-term death after carotid endarterectomy. *J Vasc Surg*. 2020;72(5):1735-1742.e3. doi:10.1016/j.jvs.2020.01.043

230. Rothenberg KA, Tucker LY, Gologorsky RC, et al. Long-term stroke risk with carotid endarterectomy in patients with severe carotid stenosis. *J Vasc Surg*. 2021;73(3):983-991. doi:10.1016/j.jvs.2020.06.124

231. Roussopoulou A, Tsivgoulis G, Krogias C, et al. Safety of urgent endarterectomy in acute non-disabling stroke patients with symptomatic carotid artery stenosis: an international multicenter study. *Eur J Neurol*. 2019;26(4):673-679. doi:10.1111/ene.13876

232. Sabeti S, Schillinger M, Mlekusch W, et al. Contralateral high-grade carotid artery stenosis or occlusion is not associated with increased risk for poor neurologic outcome after elective carotid stent placement. *Radiology*. 2004;230(1):70-76. doi:10.1148/RADIOL.2301021371

233. Sakai N, Yamagami H, Matsubara Y, et al. Prospective registry of carotid artery stenting in Japan--investigation on device and antiplatelet for carotid artery stenting. *J Stroke Cerebrovasc Dis*. 2014;23(6):1374-1384. doi:10.1016/j.jstrokecerebrovasdis.2013.11.018

234. Salem MK, Sayers RD, Bown MJ, Eveson DJ, Robinson TG, Naylor AR. Rapid access carotid endarterectomy can be performed in the hyperacute period without a significant increase in procedural risks. *Eur J Vasc Endovasc Surg*. 2011;41(2):222-228. doi:10.1016/j.ejvs.2010.10.017

235. Sayeed S, Stanziale SF, Wholey MH, Makaroun MS. Angiographic lesion characteristics can predict adverse outcomes after carotid artery stenting. *J Vasc Surg*. 2008;47(1):81-87. doi:10.1016/j.jvs.2007.09.047

236. Schillinger M, Gschwendtner M, Reimers B, et al. Does carotid stent cell design matter? *Stroke*. 2008;39(3):905-909. doi:10.1161/STROKEAHA.107.499145

237. Schmid S, Tsantilas P, Knappich C, et al. Risk of Inhospital Stroke or Death Is Associated With Age But Not Sex in Patients Treated With Carotid Endarterectomy for Asymptomatic or Symptomatic Stenosis in Routine Practice: Secondary Data Analysis of the Nationwide German Statutory Quality Assurance Database From 2009 to 2014. *J Am Heart Assoc*. 2017;6(3). doi:10.1161/JAHA.116.004764

238. Schoellhammer L, Owen-Falkenberg A, Gottschalksen B, Shahidi S. Midt-Term Results after Fast-Track Prophylactic Carotid Surgery Program: The Risk of Overlooking Occult Cancer. *J Stroke Cerebrovasc Dis*. 2018;27(3):531-538. doi:10.1016/j.jstrokecerebrovasdis.2017.10.001

239. Sef D, Skopljanac-Macina A, Milosevic M, Skrtic A, Vidjak V. Cerebral Neuromonitoring during Carotid Endarterectomy and Impact of Contralateral Internal Carotid Occlusion. *J Stroke Cerebrovasc Dis*. 2018;27(5):1395-1402. doi:10.1016/j.jstrokecerebrovasdis.2017.12.030

240. Setacci C, Chisci E, G de D, Setacci F, Sirignano P, Galzerano G. Carotid artery stenting in a single center: are six years of experience enough to achieve the standard of care? *Eur J Vasc Endovasc Surg*. 2007;34(6):655-662. doi:10.1016/j.ejvs.2007.07.008

241. Sharpe R, Sayers RD, London NJM, et al. Procedural risk following carotid endarterectomy in the hyperacute period after onset of symptoms. *Eur J Vasc Endovasc Surg*. 2013;46(5):519-524. doi:10.1016/J.EJVS.2013.08.014

242. Shobha N, Almekhlafi MA, Pandya A, et al. Carotid stenting in asymptomatic carotid stenosis: the Calgary experience. *Can J Neurol Sci*. 2010;37(5):568-573. doi:10.1017/s0317167100010714

243. Siewiorek GM, Krafty RT, Wholey MH, Finol EA. The association of clinical variables and filter design with carotid artery stenting thirty-day outcome. *Eur J Vasc Endovasc Surg*. 2011;42(3):282-291. doi:10.1016/j.ejvs.2011.04.006

244. Simonetti G, Gandini R, Versaci F, et al. Carotid artery stenting: findings based on 8 years’ experience. *Radiol Med*. 2009;114(1):95-110. doi:10.1007/S11547-008-0326-X

245. Sokol D, Fiedler J, Chlouba V, Bombic M, Priban V. Endarterectomy for asymptomatic carotid artery stenosis under local anaesthesia. *Acta Neurochir (Wien)*. 2011;153(2):363-369. doi:10.1007/S00701-010-0806-3

246. Spes CH, Schwende A, Beier F, et al. Short- and long-term outcome after carotid artery stenting with neuroprotection: single-center experience within a prospective registry. *Clin Res Cardiol*. 2007;96(11):812-821. doi:10.1007/S00392-007-0561-5

247. Squizzato F, Antonello M, Taglialavoro J, et al. Clinical Impact of Routine Cardiology Consultation Prior to Elective Carotid Endarterectomy in Neurologically Asymptomatic Patients. *Eur J Vasc Endovasc Surg*. 2020;59(4):536-544. doi:10.1016/j.ejvs.2019.11.007

248. Squizzato F, Piazza M, Forcella E, et al. Impact of Carotid Stent Design on Embolic Filter Debris Load During Carotid Artery Stenting. *Stroke*. 2023;54(10):2534-2541. doi:10.1161/STROKEAHA.123.043117

249. Stabile E, Biamino G, Sorropago G, Rubino P. Proximal endovascular occlusion for carotid artery stenting: results from a prospective registry of 1,300 patients. *J Cardiovasc Surg*. 2010;55(1):41-45. doi:10.1016/j.jacc.2009.11.079

250. Stanziale SF, Marone LK, Boules TN, et al. Carotid artery stenting in octogenarians is associated with increased adverse outcomes. *J Vasc Surg*. 2006;43(2):297-304. doi:10.1016/J.JVS.2005.10.062

251. Stelągowski M, Kasielska-Trojan A, Bogusiak K, et al. Gender-related risk factors for perioperative stroke after carotid endarterectomy in symptomatic patients. *Adv Clin Exp Med*. 2017;26(8):1225-1231. doi:10.17219/acem/68270

252. Sternbach Y, Perler BA. The influence of female gender on the outcome of carotid endarterectomy: a challenge to the ACAS findings. *Surgery*. 2000;127(3):272-275. doi:10.1067/msy.2000.104120

253. Sztriha LK, Vörös E, Sas K, et al. Favorable early outcome of carotid artery stenting without protection devices. *Stroke*. 2004;35(12):2862-2866. doi:10.1161/01.STR.0000147714.19871.45

254. Taboada CR, L DMJ, M GCJ, J PH, L MGE. Clinical Outcomes after Carotid Endarterectomy in Patients with Contralateral Carotid Occlusion. *Ann Vasc Surg*. 2016;32:83-87. doi:10.1016/j.avsg.2015.10.035

255. Tang GL, Matsumura JS, Morasch MD, et al. Carotid angioplasty and stenting vs carotid endarterectomy for treatment of asymptomatic disease: single-center experience. *Arch Surg*. 2008;143(7):653-658. doi:10.1001/ARCHSURG.143.7.653

256. Tan KT, Cleveland TJ, Berczi V, McKevitt FM, Venables GS, Gaines PA. Timing and frequency of complications after carotid artery stenting: what is the optimal period of observation? *J Vasc Surg*. 2003;38(2):236-243. doi:10.1016/s0741-5214(03)00316-1

257. Taurino M, Dezi T, Aloisi F, et al. Factors Affecting the Outcome of Symptomatic Carotid Stenosis Surgical Treatment in a Single Center Series. *Ann Vasc Surg*. 2022;83:258-264. doi:10.1016/j.avsg.2021.12.007

258. Teixeira G, Pinto PS, Silva I, et al. Carotid endarterectomy: Guidelines versus real-world practice. *Angiol Vasc Surg.* 2019;15(2):49-57.

259. Teng L, Fang J, Zhang Y, Liu X, Qu C, Shen C. Perioperative baseline β-blockers: An independent protective factor for post-carotid endarterectomy hypertension. *Vascular*. 2021;29(2):270-279. doi:10.1177/1708538120946538

260. Tigkiropoulos K, Papoutsis I, Abatzis-Papadopoulos M, et al. Thirty-Day Results of the Novel CGuard-Covered Stent in Patients Undergoing Carotid Artery Stenting. *J Endovasc Ther*. 2021;28(4):542-548. doi:10.1177/15266028211007466

261. Till JS, Toole JF, Howard VJ, Ford CS, Williams D. Declining morbidity and mortality of carotid endarterectomy. The Wake Forest University Medical Center experience. *Stroke*. 1987;18(5):823-829. doi:10.1161/01.str.18.5.823

262. Timmerman N, Rots ML, van Koeverden ID, et al. Cerebral Small Vessel Disease in Standard Pre-operative Imaging Reports Is Independently Associated with Increased Risk of Cardiovascular Death Following Carotid Endarterectomy. *European Journal of Vascular and Endovascular Surgery*. 2020;59(6):872-880. doi:10.1016/j.ejvs.2020.02.004

263. Tsivgoulis G, Krogias C, Georgiadis GS, et al. Safety of early endarterectomy in patients with symptomatic carotid artery stenosis: an international multicenter study. *Eur J Neurol*. 2014;21(10):1251-1257, e75-6. doi:10.1111/ene.12461

264. Ucci A, A de T, D’Ospina RM, et al. Carotid endarterectomy in asymptomatic octogenarians: Outcomes at 30 days and 5 years. *Vascular*. 2023;31(1):98-106. doi:10.1177/17085381211056434

265. Ucci A, D’Ospina RM, Fanelli M, et al. One-year experience in carotid endarterectomy combining general anaesthesia with preserved consciousness and sequential carotid cross-clamping. *Acta Biomed*. 2018;89(1):61-66. doi:10.23750/abm.v89i1.6814

266. Velez CA, White CJ, Reilly JP, et al. Carotid artery stent placement is safe in the very elderly (> or =80 years). *Catheter Cardiovasc Interv*. 2008;72(3):303-308. doi:10.1002/ccd.21635

267. Veraldi GF, Scorsone L, Mastrorilli D, et al. Carotid Endarterectomy with Modified Eversion Technique: Results of a Single Center. *Ann Vasc Surg*. 2021;72:627-636. doi:10.1016/j.avsg.2020.09.047

268. Wach MM, Dumont TM, Mokin M, et al. Early carotid angioplasty and stenting may offer non-inferior treatment for symptomatic cases of carotid artery stenosis. *J Neurointerv Surg*. 2014;6(4):276-280. doi:10.1136/neurintsurg-2013-010744

269. Wallaert JB, Cronenwett JL, Bertges DJ, et al. Optimal selection of asymptomatic patients for carotid endarterectomy based on predicted 5-year survival. *J Vasc Surg*. 2013;58(1):112-118. doi:10.1016/j.jvs.2012.12.056

270. White RA, Sicard GA, Zwolak RM, et al. Society of vascular surgery vascular registry comparison of carotid artery stenting outcomes for atherosclerotic vs nonatherosclerotic carotid artery disease. *J Vasc Surg*. 2010;51(5):1116-1123. doi:10.1016/J.JVS.2009.11.082

271. Winkler GA, Calligaro KD, Kolakowski S, et al. Comparison of intraoperative completion flowmeter versus duplex ultrasonography and contrast arteriography for carotid endarterectomy. *Vasc Endovascular Surg*. 2006;40(6):482-486. doi:10.1177/1538574406290846

272. Wittkugel O, Gbadamosi J, Rosenkranz M, Fiehler J, Zeumer H, Grzyska U. Long-term outcome after angioplasty of symptomatic internal carotid artery stenosis with and without stent. *Neuroradiology*. 2008;50(3):243-249. doi:10.1007/s00234-007-0326-x

273. Wong JH, Lubkey TB, Suarez-Almazor ME, Findlay JM. Improving the appropriateness of carotid endarterectomy: results of a prospective city-wide study. *Stroke*. 1999;30(1):12-15. doi:10.1161/01.str.30.1.12

274. Yu C, Han X, Zhang XL, Yu B, Dong Q. Long-term effects of white matter changes on the risk of stroke recurrence after carotid artery stenting in patients with symptomatic carotid artery stenosis. *J Neurol Sci*. 2016;369:11-14. doi:10.1016/j.jns.2016.07.062

275. Yoshida S, Bensley RP, Glaser JD, et al. The current national criteria for carotid artery stenting overestimate its efficacy in patients who are symptomatic and at high risk. *J Vasc Surg*. 2013;58(1):120-127. doi:10.1016/J.JVS.2012.12.075

276. Zahn R, Ischinger T, Mark B, et al. Embolic protection devices for carotid artery stenting: is there a difference between filter and distal occlusive devices? *J Am Coll Cardiol*. 2005;45(11):1769-1774. doi:10.1016/J.JACC.2005.02.067

277. Zharova AS, Abramov OO, Golets KO, et al. [Results of carotid endarterectomy in the acutest period of ischemic stroke]. *Zh Nevrol Psikhiatr Im S S Korsakova*. 2022;122(12. Vyp. 2):55-59. doi:10.17116/JNEVRO202212212255

278. Calo P, Oberhuber A, Görtz H. Patient Selection Criteria and Procedural Standardization for Carotid Artery Stenting—A Single Center Experience. *J Clin Med*. 2023;12(10). doi:10.3390/jcm12103534

279. Hajiyev K, Cimpoca A, Ernemann U, Bäzner H, Henkes H, von Gottberg P. Long-term outcomes of carotid stenting in a single neurovascular center: up to 12-year retrospective analysis with a focus on the influence of comorbidities. *Neuroradiology*. 2024;66(1):117-127. doi:10.1007/S00234-023-03248-0

280. Uchida K, Sakakibara F, Sakai N, et al. Real-World Outcomes of Carotid Artery Stenting in Symptomatic and Asymptomatic Patients With Carotid Artery Stenosis. *JACC Cardiovasc Interv*. 2024;17(9):1148-1159. doi:10.1016/J.JCIN.2024.03.014

281. Kang BM, Yoon SM, Oh JS, Oh HJ, Ahn JM, Yun GY. Long-term outcomes of carotid artery stenting in patients with carotid artery stenosis: A single-center 14-year retrospective analysis. *J Cerebrovasc Endovasc Neurosurg*. 2023;25(2):160-174. doi:10.7461/JCEN.2023.E2022.07.007

282. Kallmayer M, Knappich C, Kirchhoff F, et al. Determinants of Pre- and Post-Procedural Neurological Assessment, and Outcome of Carotid Endarterectomy or Stenting. *J Clin Med*. 2024;13(14). doi:10.3390/JCM13144177

283. Kök M, de Heide EJ, Hellegering J, et al. Optimizing Treatment of Significant Carotid Artery Stenosis in Times of Logistic Restraints as a Result of COVID-19 Pandemic. *Ann Vasc Surg*. 2024;108:498-507. doi:10.1016/J.AVSG.2024.05.020

284. Shahat M, Cieri E, Rocha-Neves J, Sa K. Carotid stenting: Does stent design matter? *Vascular*. 2024. 32(4) 2023:17085381231160956. doi:10.1177/17085381231160957

285. Elshikhawoda MSM, Jararaa S, Tan SHS, et al. Indications and Outcome of Carotid Endarterectomy (CEA): A Single Centre Experience. *Cureus*. 2023;15(12). doi:10.7759/CUREUS.50930

286. Sirignano P, Margheritini C, Mansour W, et al. Sex as a Predictor of Outcomes for Symptomatic Carotid Stenosis: A Comparative Analysis between CAS and CEA. *J Pers Med*. 2024;14(8). doi:10.3390/JPM14080830

287. Tresson P, Lo S, Rivoire E, Cho TH, Millon A, Long A. Long-Term Mortality and Morbidity after Carotid Endarterectomy for Symptomatic and Asymptomatic Carotid Stenosis. *Ann Vasc Surg*. 2025;110(Pt B). doi:10.1016/J.AVSG.2024.07.085

288. Vigláš P, Smolka V, Raupach J, Hejčl A, Černík D, Cihlář F. Outcome of tailored antiplatelet therapy in carotid stenting: a retrospective comparative study. *CVIR Endovasc*. 2024;7(1):18-26. doi:10.1186/S42155-024-00482-2

289. Gabrielli R, Siani A, Smedile G, Rizzo AR, Accrocca F, Bartoli S. Carotid Artery Stenting versus Carotid Endarterectomy in Terms of Neuroprotection DW-MRI Detected and Neuropsychological Assessment Impairment. *Ann Vasc Surg*. 2024;98:68-74. doi:10.1016/J.AVSG.2023.05.046

290. Nishimoto T, Oka F, Okazaki K, Sadahiro H, Oku T, Ishihara H. Safety of Tailored Transfemoral Carotid Artery Stenting for Symptomatic Elderly Patients: A Single Center Observational Study. *World Neurosurg*. 2023. 181:e1038-e1046. doi:10.1016/j.wneu.2023.11.031

291. Omura Y, Imamura H, Tani S, et al. Treatment Results of Carotid Artery Stenting with an Open-Cell Stent: Analysis of 734 Consecutive Cases at a Single Center. *World Neurosurg*. 2024;187:e453-e459. doi:10.1016/J.WNEU.2024.04.108
